# Prostatic fibroblast reprogramming by Interleukin-30 activates prostate cancer metastasis programs

**DOI:** 10.1186/s43556-026-00482-9

**Published:** 2026-06-24

**Authors:** Stefania Livia Ciummo, Carlo Sorrentino, Simona Marchetti, Cristiano Fieni, Paola Lanuti, Emma Di Carlo

**Affiliations:** 1 Department of Medicine and Sciences of Aging, “G. d’Annunzio” University of Chieti-Pescara, Via dei Vestini, Chieti, 66100 Italy; 2 Anatomic Pathology and Immuno-Oncology Unit, Center for Advanced Studies and Technology (CAST), “G. d’Annunzio” University of Chieti-Pescara, Via L. Polacchi 11, Chieti, 66100 Italy

**Keywords:** Cancer associated fibroblasts, Prostate cancer, Organ-on-chip, Tumor microenvironment, Metastasis, Cytokines

## Abstract

Prostate cancer (PC) progression and mortality are largely driven by therapeutic resistance, increasingly linked to dynamic tumor–stroma interactions. However, the molecular basis of cancer–fibroblast crosstalk remains incompletely understood, particularly the contribution of Interleukin (IL)-30, an emerging regulator of PC aggressiveness. Here, we demonstrate that IL30 produced by PC cells reprograms stromal fibroblasts via IL6Rα/gp130 signaling, activating AKT and TGF-β/BMP pathways to drive their proliferation and differentiation into pro-angiogenic cancer-associated fibroblasts (CAFs). Reciprocally, these fibroblasts amplify IL30-mediated tumor aggressiveness by enhancing oncogenic transcriptional programs and by promoting cancer cell migration. Notably, while fibroblast co-culture suppresses epithelial-mesenchymal transition (EMT)-associated genes in PC cells, including NOTCH1, SNAI1/2 and ZEB1, IL30 overexpression overrides this effect, inducing EMT regulators alongside key PC-associated genes, such as IL6, LGALS4, HAL, and SHBG, whose expression correlates with IL30 levels in clinical bone metastasis datasets. Using a two-organ-on-chip platform linking PC–fibroblast spheroids to a bone marrow–like niche, we show that fibroblasts enhance PC cell migration and colonization of the bone marrow microenvironment, effects potentiated by IL30 overexpression and largely abrogated by its genetic depletion. Collectively, these findings identify an IL30–driven tumor–stroma signaling axis that promotes microenvironmental remodeling and metastatic progression, highlighting a potential therapeutic target to counteract PC progression and treatment resistance.

**Supplementary Information:**

The online version contains supplementary material available at 10.1186/s43556-026-00482-9.

## Introduction

Prostate cancer (PC) is the second most frequently diagnosed malignancy and a leading cause of cancer-related death in men worldwide. According to GLOBOCAN 2022 estimates, approximately 1.5 million new cases and 397,000 deaths occurred globally, with higher incidence rates in developed countries [1], partly driven by population aging [2]. Despite advances in detection and treatment, preventing postoperative recurrence in localized disease and curbing progression in advanced stages remain major clinical challenges. Achieving these goals requires deeper insight into the tumor microenvironment (TME), a key driver of tumor progression and therapeutic resistance.

Stromal fibroblasts are a major component of the prostate microenvironment, essential for organ development, tissue homeostasis, and shaping the PC microenvironment. Through crosstalk with cancer cells, vasculature, nerves, immune cells, and microbiota, they influence tumor behavior and therapeutic response [3]. In the TME, fibroblasts are reprogrammed into cancer-associated fibroblasts (CAFs), a myofibroblast-like population that supports tumor growth [3]. CAFs drive tumor progression by secreting growth factors such as TGFβ1, FGF, EGF, IGF1, and VEGF, which stimulate cancer and endothelial cell proliferation. They remodel the extracellular matrix through matrix metalloproteinases [4], and recruit immunosuppressive cells, including tumor-associated macrophages and regulatory T cells [5]. In PC, CAFs also promote therapy resistance by sustaining androgen receptor signaling [6] and by modifying drug response within the TME. In clinical settings, elevated CAF levels correlate with higher Gleason scores, poor prognosis, and metastatic disease [7, 8]. Accordingly, CAF-related gene signatures are being evaluated as prognostic biomarkers, and CAF-targeted therapies are under evaluation in clinical trials [9].

Fibroblast activation is regulated by a complex network of cytokines and chemokines, including TGFβ, IL6, and CXCL12 [5]. Targeting these signaling pathways has shown promise in suppressing tumor-promoting stromal functions [10, 11]. Among TME-associated cytokines, Interleukin-30 (IL30) has recently emerged as a key mediator of tumor progression and immune regulation [12–14]. Originally characterized as a component of the IL-27 heterodimer through its association with EBI3, IL30 can also function as an autonomous cytokine [15, 16]. It is primarily produced by activated antigen-presenting cells and signals through the IL6Rα/gp130 receptor complex, activating intracellular pathways involved in cell proliferation, survival, and immune modulation [17].

In the healthy prostate, IL30 expression is low to absent, whereas in PC it is detectable in both tumor cells and infiltrating myeloid cells [13, 14, 18, 19]. Experimental studies have shown that IL30 targeting reduces tumor growth, enhances anti-tumor immunity, and inhibits metastasis in vivo [14]. From a clinical standpoint, patients with IL30–negative tumors demonstrate more favorable immune profiles and experience lower rates of biochemical recurrence after prostatectomy than those with IL30-positive tumors [14]. Although the effects of IL30 on PC cells and endothelial cells have been characterized [14, 18, 19], its role in modulating stromal fibroblasts and their impact on prostate carcinogenesis remain unexplored.

To address this gap, we investigated the impact of IL30 on human prostatic fibroblasts using co-culture systems with PC cells engineered to overexpress or lack IL30. We demonstrate that IL30 engages the IL6Rα/gp130 receptor complex in fibroblasts, activating downstream signaling pathways, including AKT, BMP, and TGFβ/Activin cascades. This signaling promotes fibroblast proliferation and resistance to apoptosis and drives their conversion into CAFs with pro-angiogenic and pro-metastatic properties. Functionally, IL30-activated fibroblasts enhance endothelial cell proliferation and vascular network formation and facilitate cancer cell migration and invasion. Moreover, IL30 regulates the bidirectional crosstalk between cancer cells and fibroblasts, modulating the expression of genes associated with tumor progression, including IL6, LGALS4, HAL, and SHBG. These findings are supported by analyses of xenograft models and transcriptomic data from metastatic PC cohorts. In a biomimetic two-organ-on-chip (2-OC) model recapitulating interactions between PC cells, fibroblasts, and the bone marrow (BM) niche, fibroblasts significantly increased cancer cell viability, migration, and BM colonization, effects that were amplified by IL30 and attenuated by its suppression. Collectively, these findings identify IL30 as a critical regulator of stromal–tumor interactions in PC. Targeting IL30 and its downstream signaling pathways may represent a promising strategy to restore TME homeostasis and overcome therapeutic resistance.

## Results

### IL30 drives prostatic fibroblast proliferation and survival

To investigate whether IL30 affects fibroblasts, we assessed IL30 receptor expression by flow cytometry in the immortalized human stromal fibroblast line WPMY-1. Although the study relied on a single cell line, WPMY-1 provides stability, uniformity, reproducibility, and suitability for investigating microenvironmental interactions.

**Fig. 1:**
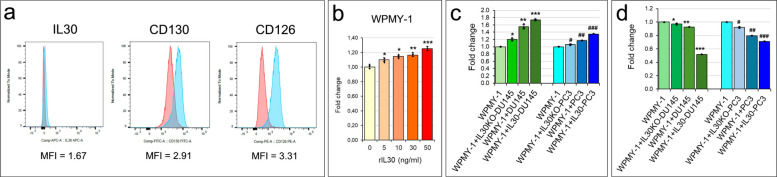
Expression of IL30 and IL6Rα/gp130 in prostatic fibroblasts and IL30-mediated regulation of prostatic fibroblast viability. **a** Cytofluorimetric analysis of IL30 expression, CD130/IL6Rβ and CD126/IL6Rα in WPMY-1 prostatic fibroblasts. Blue areas: specific Ab. Red areas: isotype control. **b** MTT assay of WPMY-1 untreated (0) or treated with rIL30 (5–50 ng/ml). ANOVA: *p* < 0.001. Tukey HSD test: *p < 0.05, *versus* 0 ng/ml; **p < 0.05, *versus* 0 and 5 ng/ml; ***p < 0.05, *versus* 0, 5 and 10 ng/ml. **c** Cytofluorimetric analysis of the proliferation (Ki67^+^ cells/µl) of WPMY-1 cells cultured alone, or co-cultured with PC cells (DU145 or PC3), IL30-overexpressing or IL30KO-PC cells. ANOVA: *p* < 0.0001. Tukey HSD test: *^,#^*p* < 0.01, *versus* WPMY-1 cells alone; **^,##^*p* < 0.01, *versus* WPMY-1 cells cultured with or without IL30KO-PC cells; ***^,###^*p* < 0.01, *versus* WPMY-1 cells cultured with or without wild type PC or IL30KO-PC cells. **d** Cytofluorimetric analysis of apoptotic events (Annexin V⁺ cells/µl) in WPMY-1 cells cultured alone or co-cultured with PC cells (DU145 or PC3), IL30-overexpressing or IL30KO-PC cells. ANOVA: *p* < 0.0001. Tukey HSD test: *^,#^*p* < 0.01, *versus* WPMY-1 cells alone; **^,##^*p* < 0.01, *versus* WPMY-1 cells cultured with or without IL30KO-PC cells; ***^,###^*p* < 0.01, *versus* WPMY-1 cells cultured with or without wild type PC or IL30KO-PC cells

WPMY-1 fibroblasts neither expressed nor secreted IL30 (IL-27/p28), but they expressed CD130 and CD126 (Fig. 1a), recognized as the functional IL30 receptor chains [17]. Recombinant (r) IL30 treatment promoted fibroblast expansion and proliferation (Fig. 1b), while reducing apoptotic cell death (Fig. S1). Moreover, blockade of CD126 and CD130 with specific antibodies (Abs) significantly impaired the proliferative response induced by IL30 (Fig. S2), supporting the involvement of the IL6Rα/gp130 receptor complex in mediating IL30 signaling and biological activity in fibroblasts.

Co-culture with wild type (WT) PC cells, DU145 and PC3, increased (ANOVA: *p* < 0.0001) the proliferation of WPMY-1 cells, which was decreased by co-culture with IL30 knockout (KO)DU145 or PC3 cells (ANOVA: *p* < 0.0001), whereas it was further enhanced by co-culture with IL30 overexpressing PC cells, IL30-DU145 and IL30-PC3 (ANOVA: *p* < 0.0001), as demonstrated by flow cytometric analysis of Ki67 staining (Fig. 1c).

Prostatic fibroblasts also acquired resistance to programmed cell death when co-cultured with PC cells, and especially with IL30-overexpressing PC cells. Conversely, co-culture with IL30KO-DU145 or IL30KO-PC3 cells blunted this anti-apoptotic effect and resulted in increased fibroblast apoptosis, as evidenced by flow cytometric analysis of Annexin V staining (Fig. 1d).

### IL30 activates AKT and TGF-β/BMP signaling pathways and promotes fibroblast reprogramming into CAFs

**Fig. 2:**
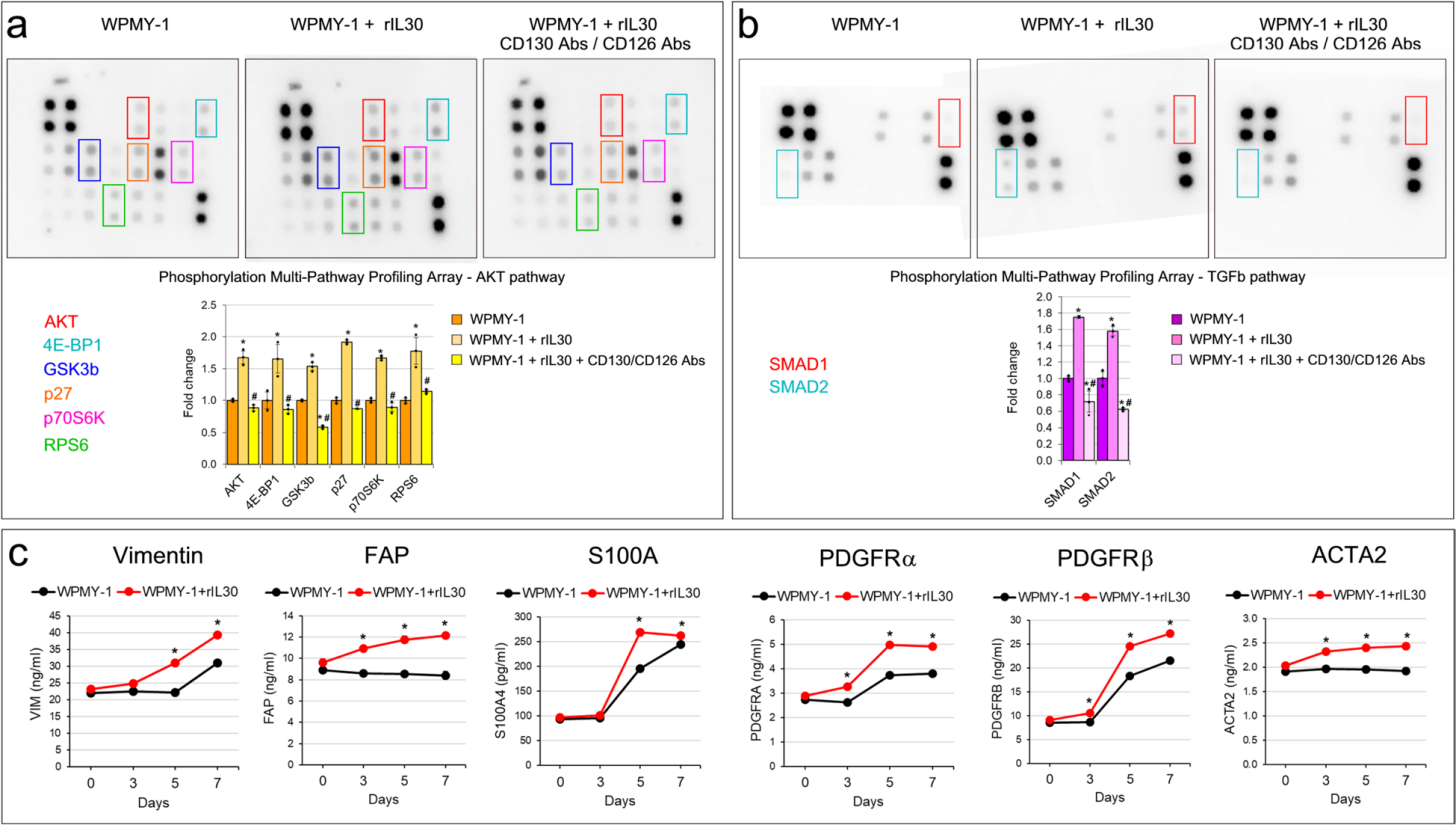
IL30 induces phosphorylation of proteins involved in IL6Rα/gp130-dependent signaling pathways and modulates the phenotype of prostatic fibroblasts. **a**, **b** Representative images of Human Phosphorylation Multi-Pathway Profiling Array. Panels show phosphorylation of proteins in the AKT (**a**) and TGFβ (**b**) pathways. Phosphorylated proteins appear as duplicate spots at the top of each panel, with spot intensity reflecting phosphorylation level. Histograms (bottom) show mean pixel density of each pair of spots, quantified using ImageJ’s Protein Analyzer. ANOVA: *p* < 0.001. Tukey HSD test: *p < 0.01, *versus* untreated WPMY-1 cells; ^#^*p* < 0.01, *versus* WPMY-1 cells + rIL30. **c** ELISA assays showing Vimentin, FAP, S100A4, PDGFRα, PDGFRβ and ACTA2 production by WPMY-1 fibroblasts untreated or treated with rIL30 (50 ng/ml) over time (0, 3, 5 and 7 days). For all markers, treatment, time, and treatment × time interaction were significant (two-way ANOVA, *p* < 0.001). *p < 0.05 for IL30-treated *versus* untreated cells at matched time points (Šidák-corrected post-hoc comparisons). Error bars and individual data points are present, but may not be visible in some cases due to minimal variability

To confirm that the effects of IL30 on prostatic fibroblasts are primarily mediated through engagement of the IL6Rα/gp130 receptor complex, a phosphorylation-based, multi-pathway profiling array was performed using lysates from rIL30–treated or untreated WPMY-1 cells. This array interrogates well-characterized signaling pathways associated with the IL6R complex, including JAK/STAT, MAPK/ERK, PI3K–AKT, AKT, and NF-κB, as well as TGF-β signaling, which can be modulated in fibroblasts downstream of gp130 activation [20]. Treatment with rIL30 enhanced the phosphorylation of multiple proteins within the AKT signaling pathway, including AKT, 4E-BP1, GSK3β, p27, p70S6K, and RPS6. This effect was abrogated by the addition of anti-CD126 and anti-gp130 Abs to the rIL30-containing culture medium (Fig. 2a), supporting a causative role for the interaction between IL30 and the IL6Rα/gp130 receptor complex. IL30 treatment also induced phosphorylation of SMAD1 and SMAD2, which belong to the BMP and TGF-β/Activin pathways, respectively, both branches of the TGFβ superfamily (Fig. 2b). The abolition of IL30’s effects by anti-CD126 and anti-CD130 blocking Abs suggests the involvement of the IL6Rα/gp130 receptor complex in IL30–mediated activation of the TGFβ pathway.

**Fig. 3:**
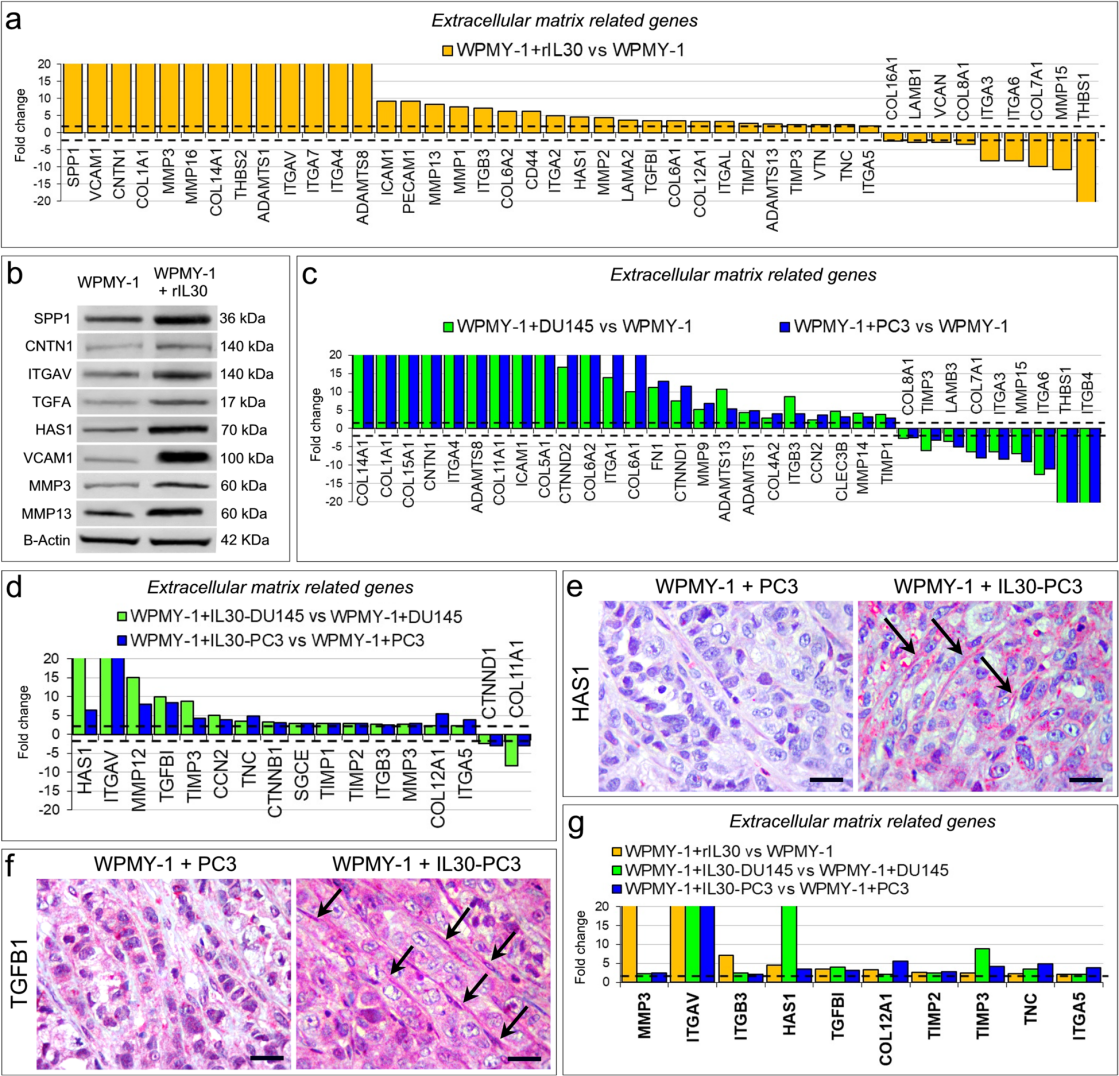
Regulation of ECM production in prostatic fibroblasts after rIL30 treatment or co-culture with IL30–overexpressing PC cells. **a** Fold changes in mRNA expression of ECM-related genes in WPMY-1 cells treated with rIL30 (50 ng/ml) compared with untreated cells. Only genes with a fold change > 2 are shown. **b** Western blot analyses of SPP1, VCAM1, CNTN1, ITGAV, TGFA, MMP3, MMP13, and HAS1 protein expression in WPMY-1 cells untreated or treated with 50 ng/ml rIL30. β-actin was used as loading control. **c** Fold changes in mRNA expression of ECM-related genes in WPMY-1 cells co-cultured with wild-type DU145 (green bars) or PC3 (blue bars) cells, compared with WPMY-1 cells cultured alone. Only genes with a fold change > 2 in both co-culture conditions are shown. **d** Fold changes in mRNA expression of ECM-related genes in WPMY-1 cells co-cultured with IL30-expressing DU145 or PC3 cells, compared with WPMY-1 cells co-cultured with WT- or EV-DU145 or PC3 cells. Only genes with a fold change > 2 in both co-culture conditions are shown. **e**, **f** Immunohistochemical staining of tumor xenografts revealed increased expression of HAS1 (**e**) and TGFB1 (**f**), primarily in prostatic WPMY-1 fibroblasts (arrows), when co-injected subcutaneously with IL30-overexpressing PC3 cells, compared with WT (or EV) PC3 cells, in NSG mice. Magnification: × 630. Scale bars: 20 µm. **g** Fold changes in mRNA expression of ECM-related genes under the following conditions: WPMY-1 cells untreated or treated with rIL30 (yellow bars); WPMY-1 cells co-cultured with WT, EV, or IL30-overexpressing DU145 (green bars) or PC3 (blue bars) cells. Only genes regulated in all three conditions are shown

The phenotype and functional status of WPMY-1 cells were also regulated by IL30, since treatment with the cytokine induced their expression and production of Vimentin, Fibroblast Activation Protein (FAP), S100A, PDGFRα, PDGFRβ, and ACTA2, which encodes for α-SMA (Fig. 2c), (as detected by Elisa assay), and upregulated the expression and production of SPP1, TGFB1, MMP2, MMP3 and ADAMTS1 (as detected by PCR array and WB) in fibroblasts (Fig. 3a, b), substantiating their switch into CAF-like cells. Furthermore, treatment with rIL30 reshaped the transcriptional profile of fibroblasts upregulating a broad range of genes coding for ECM components and adhesion molecules, including *SPP1*, *VCAM1, CNTN1, COL1A1, COL14A1, COL6A2, MMP3, MMP16, MMP13, MMP2, THBS2, ITGAV, ITGA7, ITGA4, ITGB3, ITGA2, ICAM1, PECAM1, CD44* and *HAS1*, whereas a few ECM components coding genes, primarily *ITGA3, ITGA6, COL7A1, MMP15,* and *THBS1* were downregulated (Fig. 3a).

### Crosstalk with PC cells remodels ECM programs in prostatic fibroblasts, an effect potentiated by IL30 overexpression

The fundamental role of fibroblasts in releasing ECM components and angiogenic factors within the TME has led us to investigate the ability of PC cells, and of the IL30 they produce, to regulate these functions.

Contact of fibroblasts with PC cells, both DU145 and PC3 (Fig. 3c), substantially upregulated their expression of genes coding for different types of collagens, such as *COL14A1, COL1A1, COL15A1, COL11A1, COL5A1, COL6A1* and *A2,* and *COL4A2,* along with *CCN2* (also known as CTGF) and of *CLEC3B,* known as tetranectin, which are proteins associated with tumor invasion and metastasis [21].

Integrins and adhesion molecules, such as *ITGA4, ITGA1, ITGB3, ICAM1, CNTN1*, and *CTNND1* and *CTNND2* were also upregulated in prostatic fibroblasts following co-culture with PC cells, along with members of *ADAMTS* family of metalloproteinases, especially *ADAMTS8, ADAMTS13,* and *ADAMTS1* (Fig. 3c), which are involved in tissue remodelling, angiogenesis, and cancer progression [22].

A narrow range of genes was downregulated in fibroblasts co-cultured with PC cells, including *COL8A1, COL7A1, LAMB3*, *MMP15, ITGA6, ITGB4,* and *THBS1*, which exerts multiple and context dependent roles in the TME, since it may function as a regulator of immunity (immunosuppression), angiogenesis (inhibition), tumor growth and progression (promotion or suppression) [23].

When fibroblasts were co-cultured with IL30-overexpressing DU145 or PC3 cells *versus* WT cells (Fig. 3d), their expression of integrins, enzymes and ECM components were further and substantially upregulated***,*** while together with *COLL11A1,* which promotes tumor cell aggressiveness [24], *CTNND1* was downregulated, as typically occurs in CAFs [25]. Increased production of enzymes and ECM components, including HAS1 and TGFB1, was also detected in tumor xenografts generated after subcutaneous co-injection of WPMY-1 fibroblasts with IL30-overexpressing PC cells, compared with xenografts obtained by co-injection of WPMY-1 fibroblasts with control WT or EV-PC cells in NSG mice (Fig. 3e, f; Fig. S3), as assessed by morphometric analyses of immunostained tissue sections (Table S1). Masson’s trichrome staining revealed increased ECM deposition in xenografts arising from subcutaneous co-injection of fibroblasts and IL30-overexpressing PC cells compared with xenografts derived from co-injection of fibroblasts with WT or EV PC cells (Fig. S4).

Notably, when the effects of rIL30 on fibroblasts was compared to those of the co-culture with IL30-overexpressing PC cells (Fig. 3g), they shared the upregulation of different genes coding for MMPs, TIMPs, integrins, and collagens, such as *MMP3, ITGAV, ITGB3, ITGA5, COL12A1*, along with the upregulation of cancer promoting genes, such as *HAS1, TGFB1,* and *TNC*, or genes involved in inflammation and vascular remodelling, such as *TIMP2* and *TIMP3* [26] demonstrating IL30’s ability to strengthen the pro-tumoral armamentarium of prostatic fibroblasts.

### PC cell–derived IL30 promotes a pro-angiogenic phenotype of prostatic fibroblasts

**Fig. 4:**
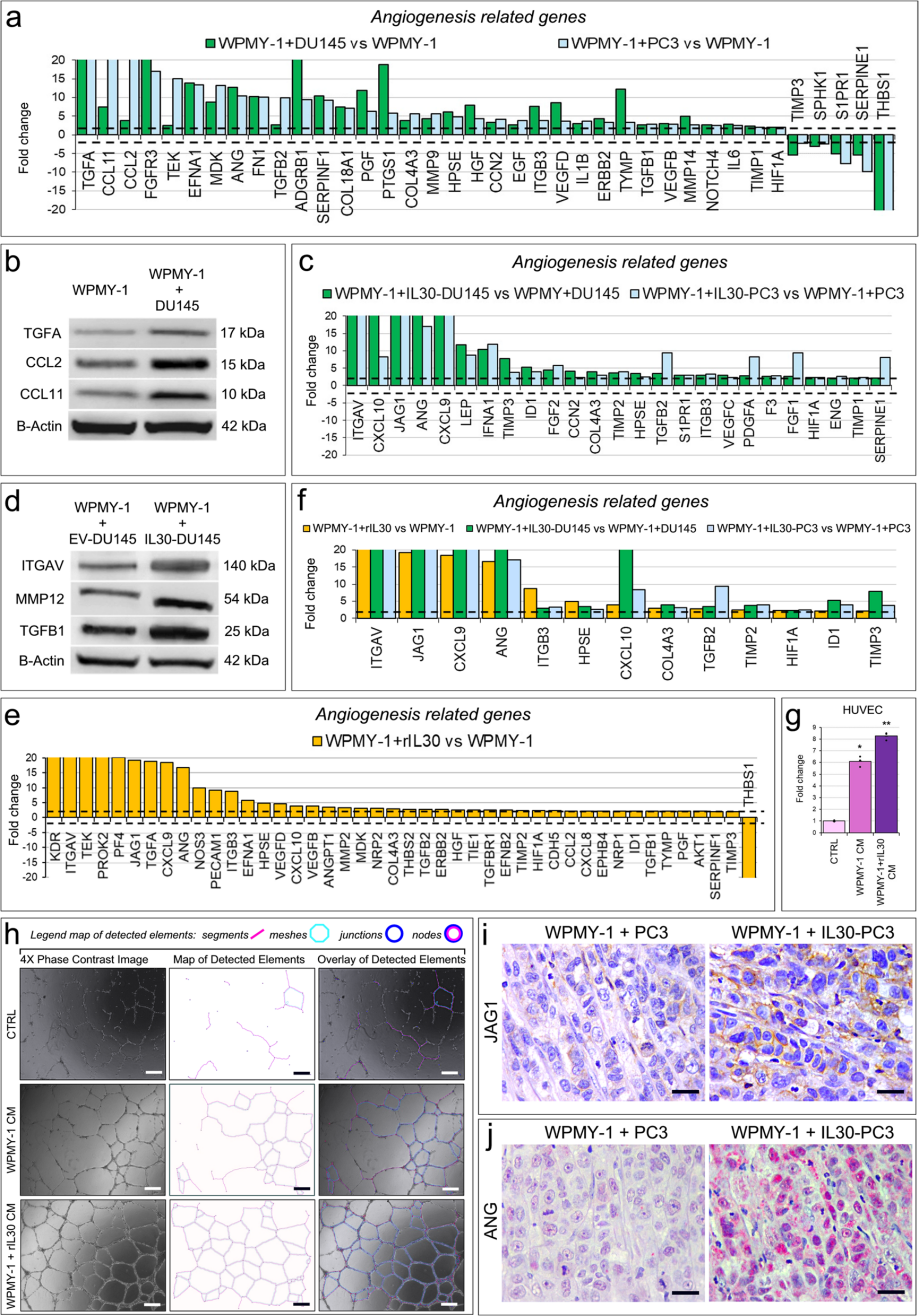
Angiogenic factor expression in fibroblasts after IL30–overexpressing PC cell co-culture and in xenografts from co-injected IL30-overexpressing PC cells. **a** Fold changes in mRNA expression of angiogenesis-related genes in WPMY-1 cells co-cultured with wild-type DU145 (green bars) or PC3 (blue bars) cells, compared with WPMY-1 cells cultured alone. Only genes with a fold change > 2 in both co-culture conditions are shown. **b** Western blot analyses of TGFA, CCL2 and CCL11 protein expression in WPMY-1 cells co-cultured or not with DU145 cells. β-actin was used as loading control. **c** Fold changes in mRNA expression of angiogenesis-related genes in WPMY-1 cells co-cultured with IL30-expressing DU145 or PC3 cells, compared with WPMY-1 cells co-cultured with WT- or EV-DU145 or PC3 cells. Only genes with a fold change > 2 in both co-culture conditions are shown. **d** Western blot analyses of ITGAV, MMP12 and TGFB1 protein expression in WPMY-1 cells co-cultured with EV- or IL30-overexpressing DU145 cells. β-actin was used as loading control. **e** Fold changes in mRNA expression levels of angiogenesis-related genes in rIL30 treated *versus* untreated WPMY-1 cells. Only genes with a fold change > 2 are shown. **f** Fold changes in mRNA expression of angiogenesis-related genes under the following conditions: WPMY-1 cells untreated or treated with rIL30 (yellow bars); WPMY-1 cells co-cultured with WT, EV, or IL30-overexpressing DU145 (green bars) or PC3 (light blue bars) cells. Only genes regulated in all three conditions are shown. **g** MTT assay of HUVEC untreated (CTRL) or treated with conditioned medium from untreated (WPMY-1 CM) or rIL30 (30 ng/ml) treated WPMY-1 (WPMY-1 + rIL30 CM) cells. One-way ANOVA, *p* < 0.001; *p < 0.01 and **p < 0.01 by Tukey’s HSD test *versus* CTRL or *versus* both CTRL and WPMY-1 CM, respectively. **h** Analyses of the tube-forming capabilities of HUVECs, treated with conditioned medium (CM) from WPMY-1 cells (WPMY-1 CM) or from rIL30–treated WPMY-1 cells (WPMY-1 + rIL30 CM), were performed using the Angiogenesis Analyzer plug-in in ImageJ. Magnification: × 4. Scale bars: 100 µm. **i**, **j** Immunohistochemical staining shows increased expression of JAG1 (**i**) and ANG (**j**) in tumor xenografts generated after subcutaneous co-injection of fibroblasts with IL30-PC3 cells, compared with co-injection with WT (or EV) PC3 cells, in NSG mice. Magnification: × 630. Scale bars: 20 µm

Beyond ECM remodeling, co-culture of fibroblasts with PC cells profoundly enhances their angiogenic potential by inducing the expression and production of a broad range of angiogenesis-related genes, most prominently *TGFA, CCL11, CCL2, FGFR3, TEK, EFNA1, MDK, ANG, FN1, TGFB2, SERPINF1, PGF, HGF* and *PTGS1*, whereas *TIMP3, SPHK1*, *S1PR1*, *SERPINE1*, and *THBS1* were downmodulated (Fig. 4a, b; Fig. S5). Overall, contact with PC cells strongly impacts the transcriptional profile of prostatic fibroblasts and shapes a multifaceted, but primarily pro-angiogenic, phenotype.

When compared to co-culture with wild type PC cells, co-culture with IL30-overexpressing PC cells led to a further increase in fibroblast expression of angiogenesis driving genes (Fig. 4c, d; Fig. S6). Several angiogenesis drivers were upregulated in fibroblasts also following rIL30 treatment, mostly the expression *of KDR, ITGAV, TEK, PROK2, PF4, TGFA, ANG, NOS3, PECAM1, ITGB3, EFNA1, HPSE, VEGFD* and *JAG1* (Figs. 3b and 4e). Several angiogenesis drivers, in particular *ITGAV, CXCL9, CXCL10, ANG, HPSE, JAG1, COL4A3, TIMP2-3, HIF1A* and *ID1*, were upregulated in both rIL30 treated fibroblasts and in fibroblasts co-cultured with IL30-overexpressing PC cells (Fig. 4f). Consistent with these findings, conditioned medium (CM) from rIL30–stimulated WPMY-1 cells significantly increased the proliferation of ECs (HUVECs) compared with CM from untreated WPMY-1 cells (*p* < 0.01). In turn, CM from untreated WPMY-1 cells also increased EC proliferation compared with control (CTRL) cells (Fig. 4g). The functional significance of the IL30–induced pro-angiogenic program in fibroblasts was further assessed by examining the effects of CM from untreated and rIL30–stimulated WPMY-1 cells using an endothelial tube formation assay. Quantitative analysis of capillary-like network parameters, using ImageJ Angiogenesis Analyzer plug-in, revealed that ECs exposed to CM from untreated fibroblasts exhibited a significant (ANOVA: *p* < 0.001) increase in the number of tubes, meshes, junctions, and nodes per field compared with untreated CTRL, whereas ECs exposed to CM from rIL30-stimulated fibroblasts (30 ng/ml or 50 ng/ml) showed a further significant increase in these parameters (ANOVA: *p* < 0.001) compared to both ECs cultured with CM from untreated fibroblasts and control ECs (Fig. 4h; Fig. S7). Collectively, these data indicate that IL30 stimulation enhances the pro-angiogenic activity of fibroblasts, thereby promoting microvascular network formation.

Moreover, immunopathological analyses revealed a distinct in vivo expression pattern of angiogenic mediators, including JAG1 and ANG, along with more robust stromal architecture and increased ECM content, in tumour xenografts grown in NSG mice after subcutaneous co-injection of WPMY-1 and IL30-overexpressing PC cells, when compared to control tumours (Fig. 4i, j; Fig. S8 and Table S1), strongly suggesting a key role for IL30 in regulating angiogenesis and ECM remodelling programs in prostatic fibroblasts.

### IL30 modulates PC–fibroblast crosstalk and reprograms cancer driver gene expression in PC cells

Since contact with stromal fibroblasts can affect different, and mostly unknown, aspects of cancer biology, the expression of cancer driver and regulatory genes, was investigated in DU145 and PC3 cells after co-culture with prostatic WPMY-1 fibroblasts.

**Fig. 5:**
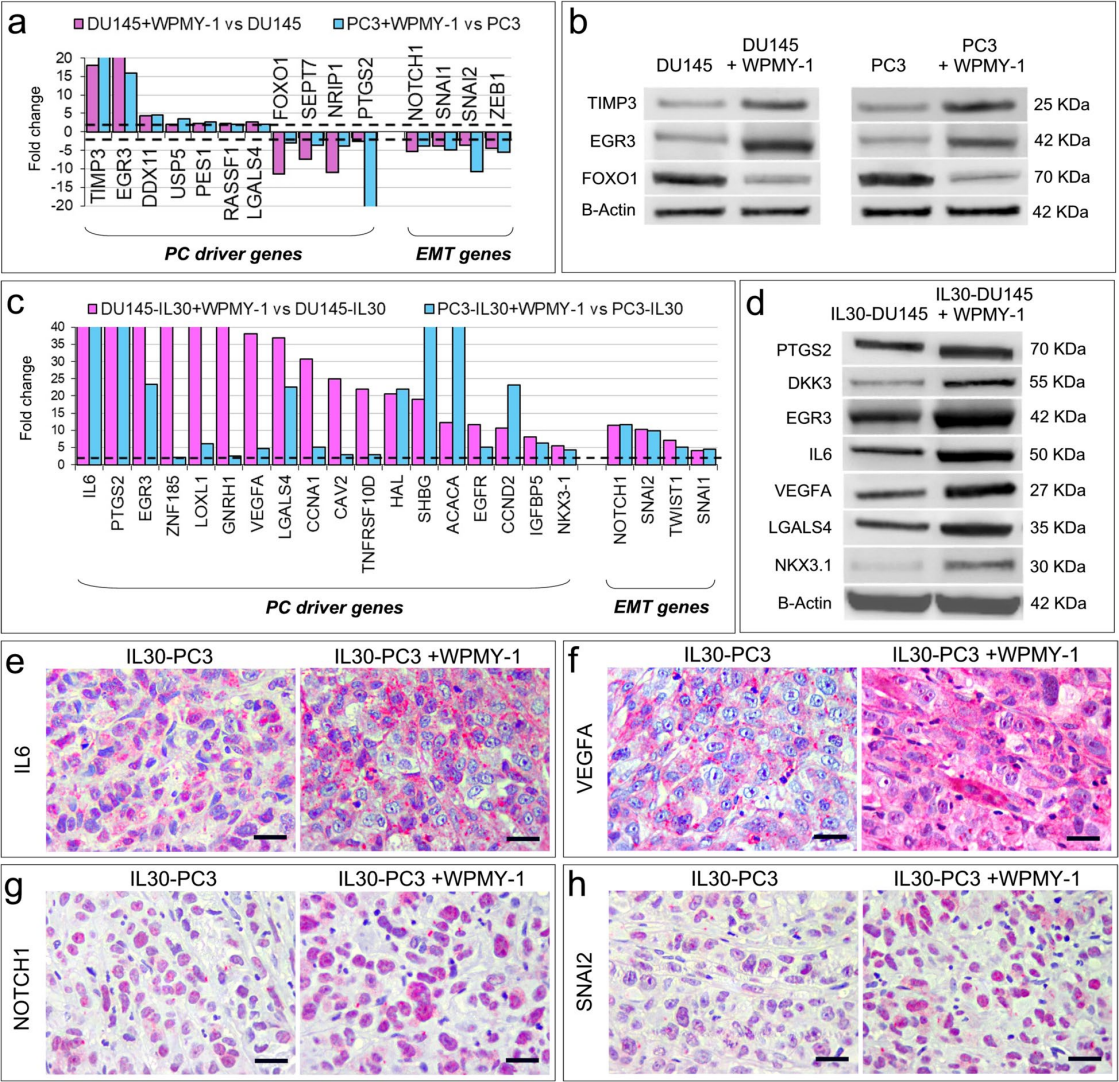
Prostate cancer driver and regulatory gene expression in IL30–overexpressing PC cells after fibroblast co-culture and in xenografts from co-injected fibroblasts. **a** Fold changes in mRNA expression of PC driver and EMT genes in wild-type DU145 (fuchsia bars) or PC3 (blue bars) cells cultured alone or co-cultured with WPMY-1 cells. Only genes with a fold change > 2 in both co-culture conditions are shown. **b** Western blot analyses of TIMP3, EGR3 and FOXO1 protein expression in DU145 cells co-cultured or not with WPMY-1 cells. β-actin was used as loading control. **c** Fold changes in mRNA expression of PC driver and EMT genes in IL30-overexpressing DU145 (fuchsia bars) or PC3 (blue bars) cells cultured alone or co-cultured with WPMY-1 cells. Only genes with a fold change > 2 in both co-culture conditions (IL30-DU145 and IL30-PC3) are shown. **d** Western blot analyses of PTGS2, DKK3, EGR3, IL6, VEGFA, LGALS4, and NKX3.1 protein expression in IL30-DU145 cells co-cultured or not with WPMY-1 cells. β-actin was used as loading control.** e**, **f**, **g**, **h** Immunohistochemical analysis reveals increased expression of IL6 (**e**), VEGFA (**f**), NOTCH1 (**g**), and SNAI2 (**h**) in tumor xenografts generated by subcutaneous injection of IL30-PC3 cells, either alone or in combination with prostatic WPMY-1 fibroblasts, in NSG mice. Magnification: × 630. Scale bars: 20 µm

In both PC cell lines, contact with fibroblasts activated cancer progression programs, by upregulating *DDX11* [27], *USP5* [28], *PES1* [29] and *LGALS4* [30] and downregulating tumor suppressors like *FOXO1* [31] (Fig. 5a, b). However, the concurrent upregulation of tumor suppressors, such as *TIMP3* [32] (Fig. 5a, b), and *RASSF1* [33], along with the downregulation of cancer driver genes, such as *PTGS2* [34] (Fig. 5a), suggested that quiescent prostatic fibroblasts may actively restrain tumor growth and progression. Indeed, co-culture with prostatic fibroblasts repressed PC cell expression of key epithelial–mesenchymal transition (EMT) genes, such as *NOTCH1, SNAI1, SNAI2* and *ZEB1* (Fig. 5a).

To determine whether IL30 over-expression in PC cells interferes with the effects caused by co-culture with fibroblasts on cancer cells’ transcriptional profiles, the expression of PC driver and regulatory genes was assessed in IL30-overexpressing PC cells co-cultured or not with prostatic fibroblasts.

In IL30-overexpressing PC cells, contact with fibroblasts (Fig. 5c, d; Fig. S9) substantially enhanced the expression levels of PC regulatory genes, including *EGR3*, *SHBG, NRIP1,* and *GNRH1*, as well as cancer drivers, such as *IL6, PTGS2, ZNF185, SOX4, CCND2, VEGFA, LOXL1, LGALS4*, *HAL, ACACA.* This interaction also enhanced the expression of EMT-related genes, including *NOTCH1* and *SNAI2*, as confirmed by immunohistochemical analysis of tumor xenografts generated by subcutaneous co-injection of WPMY-1 fibroblasts and IL30-overexpressing PC cells (Fig. 5e-h; Table S2; Fig. S10). Intriguingly, contact with fibroblasts also upregulated in IL30-overexpressing PC cells tumour-suppressor genes, such as *NKX3-1* [35] (Fig. 5c, d). These findings underscore the tumor-promoting role of IL30 and highlight the dual potential of prostatic fibroblasts, which can either restrain or facilitate PC progression.

### IL30-activated fibroblasts promote prostate cancer cell motility and directional migration

**Fig. 6:**
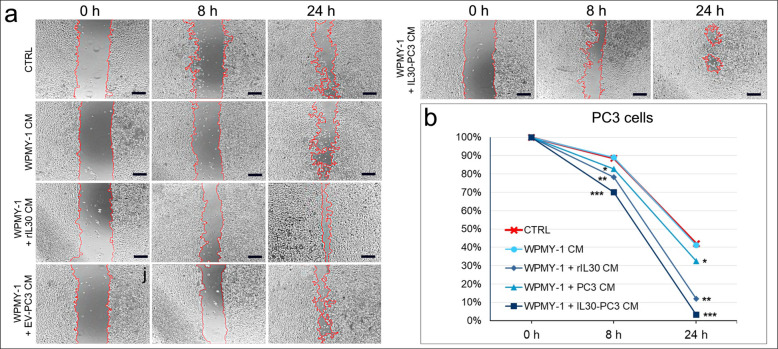
Migratory capability of PC cells promoted by IL30–conditioned fibroblasts. **a**, **b** Representative images (**a**) and corresponding statistical analyses (**b**) of PC cell migratory activity across different experimental conditions, evaluated using the wound-healing assay. **a** Representative field images from an in vitro wound healing assay performed on PC3 cell monolayers either untreated (CTRL) or treated with conditioned media (CM) derived from: WPMY-1 cells; rIL30–treated WPMY-1 cells; EV-PC3–WPMY-1 co-cultures; or IL30-PC3–WPMY-1 co-cultures. Red lines indicate the wound margins. Magnification: × 4. Scale bars: 100 µm. **b** Statistical analysis was performed using two-way ANOVA, revealing significant effects of treatment (*p* < 0.0001), time (*p* < 0.0001), and their interaction (*p* < 0.0001). Post hoc Welch’s *t*-tests with Šidák correction: *p < 10⁻^3^, *versus* CTRL and WPMY-1 CM; **p < 10⁻^3^, *versus* CTRL, WPMY-1 CM, and WPMY-1 + PC3 CM; ***p < 10⁻^3^, *versus* CTRL, WPMY-1 CM, WPMY-1 + rIL30 CM, and WPMY-1 + PC3 CM. Individual data points are included; however, in some cases both the data points and error bars may not be clearly visible due to the high consistency among replicates

To assess whether the transcriptional reprogramming induced by fibroblast co-culture in wild type (or empty vector) and IL30-overexpressing PC cells was associated with functional changes in cancer cell migration and invasion, a wound-healing assay was performed (Fig. 6a, b; Fig. S11). While treatment with CM from quiescent WPMY-1 cells (WPMY-1 CM) had no effect on PC cell migration, exposure to CM from WPMY-1 cells stimulated with rIL30 (WPMY-1 + rIL30 CM) resulted in a marked enhancement of wound closure compared with both untreated PC cells and PC cells treated with WPMY-1 CM. Microscopic analysis revealed a higher density of migrating cells at the wound edge in PC cell monolayers treated with CM from WPMY-1 + rIL30 or from co-cultures of WPMY-1 cells with IL30-overexpressing PC3 or DU145 cells. A significant increase in cell migration was detected as early as 8 h post-scratch, with treated PC3 or DU145 cells exhibiting faster wound-closure rates (Two-way ANOVA:* p* < 0.0001). At 24 h, PC3 and DU145 cell cultures exposed to conditioned medium (CM) from WPMY-1 fibroblasts stimulated with rIL30 exhibited significantly enhanced wound closure, reaching 88% and 87%, respectively, compared with 58% and 54% in control (CTRL) groups (two-way ANOVA, *p* < 0.0001) (Fig. 6a, b; Fig. S11). These results demonstrate that IL30–activated fibroblasts release soluble mediators that markedly enhance PC cell motility and directional migration, critical steps in the metastatic cascade.

The enhanced wound closure observed, by 24 h, in PC cells treated with CM from co-cultures of WPMY-1 cells with EV-PC3 or EV-DU145 cells (67% and 58%, respectively), and more prominently with IL30-PC3 or IL30-DU145 cells (97% and 94%, respectively) (Fig. 6a, b; and Fig. S11), reinforces the functional significance of cancer–stromal fibroblast crosstalk and highlights IL30’s role in driving PC metastasis. Although fibroblasts can repress some PC progression genes, their overall influence on PC cells via co-culture is predominantly tumor-promoting, ultimately driving disease progression.

### Bioinformatic analysis of metastatic prostate cancer cohorts highlights IL30-associated, fibroblast-driven transcriptional programs

**Fig. 7:**
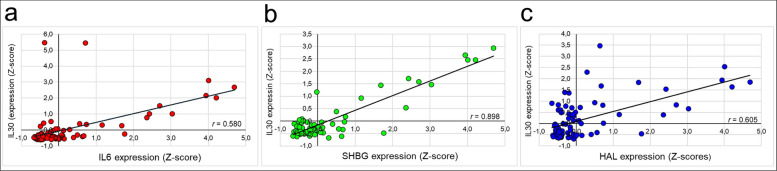
Correlation between the expression of PC driver genes and expression of IL30 in bone metastasis clinical samples. **a**, **b**, **c** Correlation between the expression levels of IL30 and that of IL6, SHBG and HAL, obtained from RNA-Seq data of bone metastases biopsies from the *Metastatic Prostate Adenocarcinoma (SU2C/PCF Dream Team)* dataset. The scatter plots display individual samples, and the regression lines indicate the direction and strength of the association.* r* = Pearson correlation coefficient

Given the tumor-promoting transcriptional program induced by IL30 in prostatic fibroblasts and their capacity to regulate PC progression pathways, we investigated whether IL30-driven fibroblast–PC cell crosstalk influences PC cell migration and metastatic potential. Bioinformatic analysis of RNA-Seq data from metastatic lesions of 201 patients included in the Metastatic Prostate Adenocarcinoma (SU2C/PCF Dream Team) cohort demonstrated, through Pearson correlation analysis, moderate-to-strong positive associations between IL30 expression and several key PC regulatory genes upregulated in IL30-overexpressing PC cells following fibroblast co-culture. These included IL6 (r = 0.337), LGALS4 (r = 0.440), HAL (r = 0.648), and SHBG (r = 0.734), among the most strongly induced transcripts observed after co-culture with prostatic fibroblasts (Fig. 5c). Additional analysis focused on transcriptional profiles from bone metastases, the most common site of PC progression [36], derived from biopsy samples of 82 PC patients, revealed strong to very strong correlations between IL30 and IL6 (r = 0.580), HAL (r = 0.605), and particularly SHBG (r = 0.898) (Fig. 7a-c). These findings support the involvement of IL30-associated, fibroblast-enhanced genes in PC progression and bone metastatic colonization.

### Functional impact of IL30-driven tumor–stromal crosstalk in a PC–bone marrow organ-on-chip model

To investigate the functional contribution of tumor–stromal interactions in this process, we employed a two-compartment PC–BM organ-on-chip platform (HUMIMIC Chip2, TissUse, Berlin, Germany). This microfluidic bioreactor comprised a 3D PC cell–fibroblast spheroid culture connected through pulsatile flow to a second chamber containing a hydroxyapatite-coated ceramic scaffold seeded with human BM-derived mesenchymal stromal cells (MSCs; CD105 ^+^, CD166 ^+^, CD44 ^+^, CD90 ^+^, CD73 ^+^) and CD34 ^+^ hematopoietic stem/progenitor cells (HSPCs), thereby reproducing key structural and cellular features of the BM niche.

**Fig. 8:**
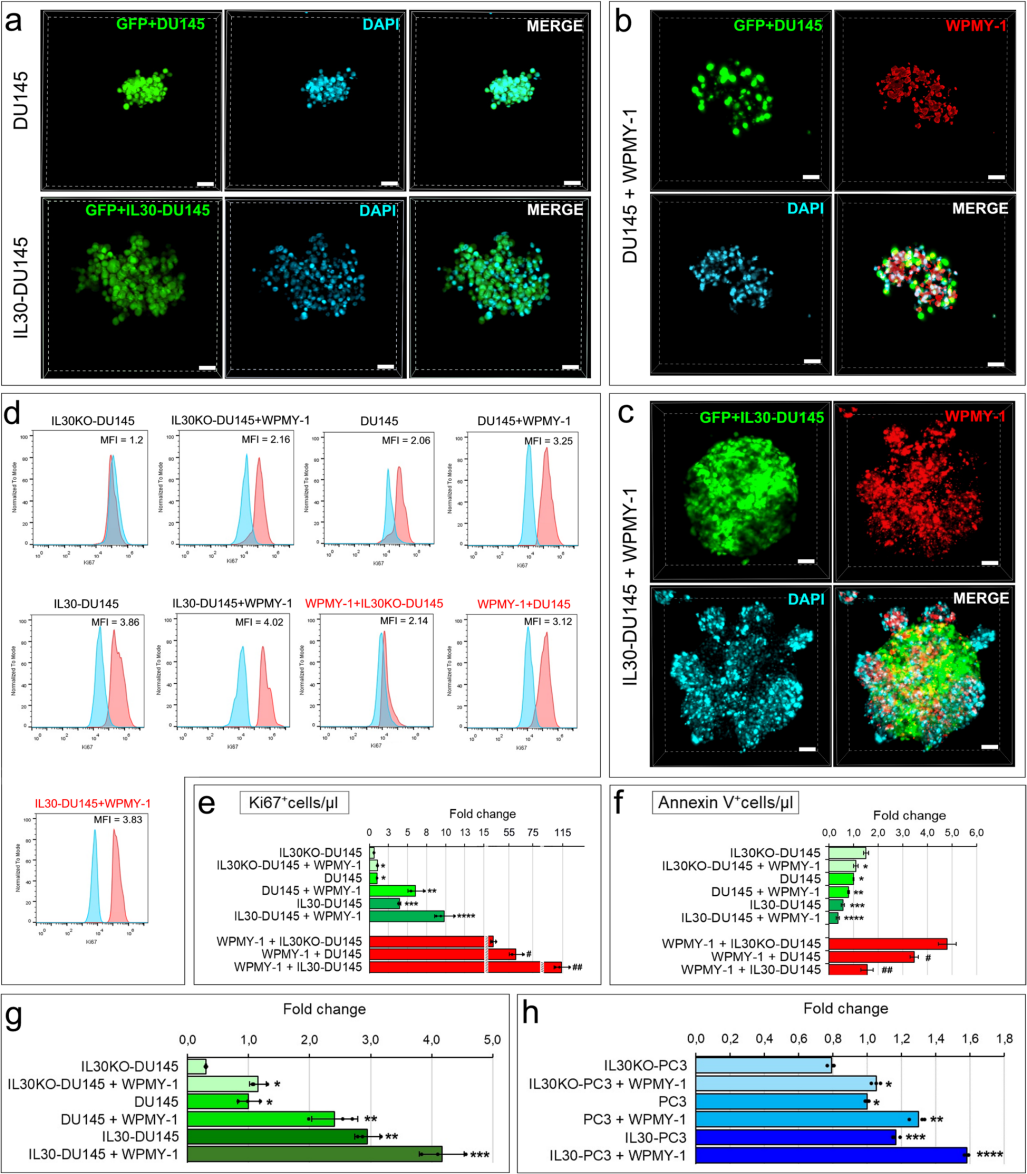
Bone marrow colonization of IL30-overexpressing or IL30 knocked out PC cells co-cultured with prostatic fibroblasts in 3D spheroids housed into the 2-OC Device. **a**, **b**, **c** Confocal microscopy images of tumor spheroids, developed into the 2-OC device, containing: (**a**) wild type or IL30-overexpressing DU145 cells; (**b**) wild type DU145 cells co-cultured with WPMY-1 cells; (**c**) IL30-DU145 cells co-cultured with WPMY-1 cells. Magnification: × 200. Scale bars: 30 µm. **d** Flow cytometry analysis of Ki67 fluorescence in EV-DU145, IL30KO-DU145 and IL30-DU145 cells, cultured with or without WPMY-1 cells, and in WPMY-1 cells co-cultured with EV-DU145, IL30KO-DU145 or IL30-DU145 cells, in 2-OC. Red areas: specific Ab. Blue areas: isotype control. **e**, **f** Proliferation and apoptosis of DU145, IL30KO-DU145 and IL30-DU145 cells cultured with or without WPMY-1 cells (green bars), and proliferation and apoptosis of WPMY-1 cells cultured with DU145, IL30KO-DU145 or IL30-DU145 cells (red bars), in the 2-OC device, as assessed by flow cytometry with Ki67^+^ or Annexin V staining. ANOVA: *p* < 0.001. Tukey HSD test: *p < 0.05, *versus* IL30KO-DU145 cells; **p < 0.05, *versus* IL30KO-DU145 cells, cultured with or without WPMY-1 cells, and DU145 cells; ***p < 0.05, *versus* IL30KO-DU145 cells, cultured with or without WPMY-1 cells, and DU145 cells, cultured with or without WPMY-1 cells; ****p < 0.05, *versus* IL30KO-DU145 cells, cultured with or without WPMY-1 cells, DU145 cells, cultured with or without WPMY-1 cells, and IL30-DU145 cells; ^#^*p* < 0.01, *versus* WPMY-1 + IL30KO-DU145 cells; ^##^*p* < 0.01, *versus* WPMY-1 cells cultured with IL30KO-DU145 or DU145 cells. **g** Flow cytometry analysis of GFP⁺ DU145, IL30KO-DU145 and IL30-DU145 cells, cultured with or without WPMY-1 cells, which migrate and colonize the BM scaffold in the 2-OC device. ANOVA: *p* < 0.001. Tukey HSD test: *p < 0.05, *versus* IL30KO-DU145 cells; **p < 0.05, *versus* IL30KO-DU145 cells, cultured with or without WPMY-1 cells, and DU145 cells; ***p < 0.05, *versus* IL30KO-DU145 cells, cultured with or without WPMY-1 cells, DU145 cells, cultured with or without WPMY-1 cells, and IL30-DU145 cells. **h** Flow cytometry analysis of GFP⁺ PC3, IL30KO-PC3 and IL30-PC3 cells, cultured with or without WPMY-1 cells, which migrate and colonize the BM scaffold in the 2-OC device. ANOVA: *p* < 0.001. Tukey HSD test: *p < 0.01, *versus* IL30KO-PC3 cells; **p < 0.01, *versus* IL30KO-PC3 cells, cultured with or without WPMY-1 cells, and PC3 cells; ***p < 0.01, *versus* IL30KO-PC3 cells, cultured with or without WPMY-1 cells, and PC3 cells, cultured with or without WPMY-1 cells; ****p < 0.01, *versus* IL30KO-PC3 cells, cultured with or without WPMY-1 cells, PC3 cells, cultured with or without WPMY-1 cells, and IL30-PC3 cells

Confocal microscopy analyses (Fig. 8a–c) and flow cytometry evaluation (Fig. 8d) demonstrated that the viability and proliferative activity of wild-type (or empty vector), IL30KO, and IL30-overexpressing PC cells were significantly enhanced when tumor spheroids included fibroblasts, compared with spheroids lacking stromal cells (ANOVA, *p* < 0.001). Increased proliferation was evidenced by a higher proportion of Ki67 + cells (Fig. 8d, e), consistent with findings obtained in 2D co-culture systems (Fig. 1c), whereas apoptotic events, assessed by Annexin V staining, were markedly reduced (ANOVA, *p* < 0.001) (Fig. 8f; Fig. S12). These data confirm the supportive role of stromal fibroblasts in sustaining PC cell survival, proliferation, and expansion within a biomimetic microenvironment.

Importantly, fibroblasts isolated from spheroids containing IL30-overexpressing PC cells displayed significantly increased proliferation and reduced apoptosis compared with fibroblasts co-cultured with wild-type (or empty vector) PC cells (ANOVA, *p* < 0.001) (Fig. 8d–f; Fig. S13–15). Conversely, fibroblasts recovered from spheroids containing IL30KO-PC3 or IL30KO-DU145 cells exhibited significantly lower proliferative rates and increased apoptotic cell death (ANOVA, *p* < 0.001) (Fig. 8e, f; Fig. S14). These findings further support the ability of IL30 to protect fibroblasts from programmed cell death and maintain their survival, even beyond the effects mediated by baseline tumor–stromal co-culture.

Assessment of PC cell migration from tumor spheroids toward the BM-like scaffold after 8 days of co-culture revealed a marked increase in GFP^+^ PC cells colonizing the BM compartment when cancer cells were co-cultured with WPMY-1 fibroblasts (Fig. 8g, h; Fig. S16). IL30 overexpression further enhanced migration toward the BM-like niche, whereas IL30KO-PC cells displayed reduced dissemination, consistent with our previous findings [37]. Notably, fibroblast co-culture strongly potentiated BM colonization across experimental conditions, indicating that stromal fibroblasts and the signaling networks activated through tumor–stromal crosstalk critically contribute to PC dissemination and metastatic seeding. Collectively, these findings identify IL30 as a key mediator capable of reprogramming prostatic fibroblasts toward a CAF-like phenotype that actively supports PC progression and bone metastasis.

## Discussion

The prostatic stroma, particularly fibroblasts that organize the extracellular matrix and maintain tissue architecture, plays a pivotal yet still debated role in PC initiation, metastatic progression, and therapeutic resistance [38]. These cells actively remodel the TME, shaping immune responses and tumor behavior. Dissecting fibroblast heterogeneity and the upstream signals that govern their functional states is critical for the rational design of more effective therapeutic strategies.

Increasing evidence underscores the stroma’s dual role, as it can both restrain and promote tumor progression, ultimately influencing treatment outcomes [39, 40]. In PC, activated CAF subsets, including FN1⁺/FAP⁺ matrix-remodeling CAFs [41], CXCL12⁺/CXCL14⁺ inflammatory CAFs [11], and iron-loaded CAFs [42], enhance aggressiveness, immune evasion, and therapy resistance, whereas AR-active, p53-intact, or quiescent fibroblasts may counteract tumor progression [43]. Although derived from a single model of prostatic stromal fibroblasts, our gene expression data support a dual regulatory role for stromal fibroblasts in PC. In co-culture with PC cells, fibroblasts upregulated tumor-promoting genes, such as PES1 and LGALS4, alongside tumor suppressors including TIMP3 and RASSF1, while also downregulating additional tumor suppressors (FOXO1), EMT-related genes, and pro-tumorigenic mediators, such as PTGS2. Despite this mixed transcriptional response, fibroblast–PC interactions ultimately promote tumor aggressiveness, as demonstrated by wound-healing assays showing enhanced migration and invasion of PC cells exposed to conditioned media from co-cultures, findings further validated using a 2-OC device-based model [44]. Within this bidirectional communication, PC cells reprogram fibroblasts toward a pro-angiogenic, tumor-supportive state characterized by increased secretion of ECM components, growth factors, and matrix-remodeling enzymes.

Given the emerging evidence implicating IL30 in PC initiation, progression, and metastatic evolution [13, 14, 19, 45], we investigated whether IL30 contributes to this stromal reprogramming by acting on fibroblasts, the predominant cellular component of the PC microenvironment [3]. Our findings demonstrate that IL30 directly targets fibroblasts expressing its putative receptor, the IL6Rα/gp130 complex, promoting their survival, enhancing proliferation, and driving their conversion into CAFs by reshaping both their phenotype and transcriptional profile.

In fibroblasts, IL30 activates both the AKT and TGF-β/BMP signaling pathways, as demonstrated by increased phosphorylation of key components of the AKT cascade (AKT, 4E-BP1, GSK3β, p27, p70S6K, and RPS6) and of SMAD1/2, downstream effectors of TGF-β/BMP signaling [46]. These effects are dependent on the IL6Rα/gp130 receptor complex, as they are abolished by neutralizing antibodies against CD126 and CD130, highlighting IL30’s role in coordinating multiple pro-survival and growth-related signalling pathways. In addition, IL30 interferes with the fibroblast–PC relationship by boosting fibroblasts’ ability to express adhesion molecules, such as ITGAV, a fundamental pathway regulating fibrosis [47]; to secrete ECM components that drive matrix and vascular remodelling, such as COL4A3 and TIMPs; to release growth and angiogenic regulators, including JAG1 [48], ANG [49], and TGFB2 [50]; and to produce immunoregulatory chemokines, such as CXCL9 and CXCL10. These chemokines exert pleiotropic effects in cancer, promoting T lymphocyte and NK cell recruitment to enhance antitumor immunity [51] and inhibiting angiogenesis and tumor progression, although context-dependent pro-tumor effects may arise via autocrine or cellular interactions [52]. IL30 overexpression in cancer cells strengthens the ability of fibroblasts to remodel the transcriptional profile of PC cells themselves toward a pro-metastatic direction, as demonstrated by the upregulation of a wide range of inflammatory and metastasis promoting genes, such as *IL6, PTGS2, LOXL1, VEGFA, LGALS4, CCNA1, CAV2, TNFRSF10D, HAL, ACACA* [53], *EGFR* [54], *NOTCH1, SNAI2, TWIST1* and *SNAI1* [55], which overrides the induction of the few tumor suppressors, such as *ZNF185* [56]. Indeed, most PC driver or regulatory genes upregulated in IL30-overexpressing PC cells after co-culture with fibroblasts, including *IL6, EGR3, VEGFA, LGALS4, SHBG* and *CCND2* were similarly regulated after co-culture with ECs [57], suggesting a concomitant responsiveness of these stromal cells to IL30 and their influence on the gene expression and the behaviour of PC cells. On the other hand, co-culture with IL30-overexpressing PC cells activates in both endothelia and fibroblasts the expression of critical angiogenesis regulatory factors, such as JAG1, ITGAV and CXCL10 [57]. The proangiogenic program activated in prostatic fibroblasts by recombinant, or PC cell derived, IL30 was demonstrated by increased endothelial cell proliferation and microvessel formation in response to conditioned media from IL30-treated fibroblasts. The concomitant reshaping of ECM-related gene expression in fibroblasts exposed to recombinant IL30, or co-cultured with IL30-overexpressing PC cells, contributes to the robust stromal component observed in IL30-overexpressing and fibroblast-enriched tumor xenografts compared with controls. In vivo studies in NSG mice confirm molecular findings, showing strong nuclear expression of NOTCH1 and SNAI2 in IL30-overexpressing tumor cells, alongside upregulation of pro-tumorigenic mediators such as JAG1, ANG, HAS1, and TGFB1 in associated fibroblasts.

Although the 2-OC platform [44], which connects 3D fibroblast–PC spheroids to a BM–like niche under pulsatile flow, does not fully recapitulate the systemic complexity of metastatic PC, it provides a physiologically relevant model of tumor–stromal interactions, offering insight into how primary tumor fibroblasts promote PC cell proliferation and metastatic dissemination to bone. These effects are reduced by IL30 knockout in PC cells and enhanced by IL30 overexpression. Consistently, RNA-seq analysis of metastasis samples from the SU2C/PCF Dream Team cohort revealed a moderate-to-strong positive correlation between IL30 expression and PC regulatory genes induced in IL30-overexpressing PC cells following fibroblast co-culture, including IL6 [32], LGALS4, HAL [58], and SHBG [59], highlighting IL30-driven transcriptional reprogramming during fibroblast–tumor crosstalk.

IL6 acts as both a paracrine and autocrine mediator in the tumor–bone microenvironment. Produced by osteoblasts, stromal cells, and metastatic PC cells, IL6 activates STAT3-dependent signaling pathways promoting tumor cell survival and proliferation. Concurrently, IL6 induces RANKL expression in osteoblastic cells and enhances osteoclast differentiation, leading to increased bone resorption that facilitates tumor colonization and sustains a pro-inflammatory niche [60].

In advanced PC, upregulation of Galectin-4 and O-glycosylation enzymes C1GALT1 and ST3GAL1 correlates with disease progression. Galectin-4 binding to O-glycans activates receptor tyrosine kinase signaling and SOX9-associated stemness pathways, reinforcing MYC-driven metabolic and glycosylation programs that promote tumor aggressiveness and worse survival [61].

HAL, the rate-limiting enzyme of histidine catabolism, may modulate the TME by altering metabolic and immune signaling networks. Although HAL expression varies across cancers, mechanistic evidence in PC remains limited. Emerging data suggest that dysregulated histidine metabolism may contribute to therapy resistance, immune evasion, and tumor progression through metabolic reprogramming [62].

SHBG is a liver-derived glycoprotein that regulates circulating testosterone and dihydrotestosterone bioavailability. Systemic SHBG levels are associated with PC risk and progression in a context-dependent manner [59], while prostate-derived SHBG may activate androgen-independent signaling affecting proliferation and differentiation [63]. Its dysregulation reflects androgen-driven disease and associates with progression, therapy resistance, and relapse risk [64].

In conclusion, our integrated approach, combining in vitro experiments, immunopathological analyses of tumor xenografts, and the 2-OC platform linking PC–fibroblast spheroids to a BM–like niche, highlights the pivotal role of prostatic fibroblasts in shaping tumor transcriptomic programs and their capacity to either promote or restrain PC progression. Within this crosstalk, we uncover IL30 as a novel mediator driving fibroblast reprogramming into proangiogenic, tumor supportive CAFs. Beyond its established role in vascular endothelia [19], IL30 empowers prostatic fibroblasts to activate cancer driver genes and molecular programs that foster bone metastatic colonization, revealing a critical mechanism of PC microenvironment remodeling. While the model may not fully capture systemic complexity, these findings collectively position IL30 as a promising therapeutic target to counteract stromal-driven progression and treatment resistance in advanced PC.

## Materials and methods

### Cell cultures

Human prostate cancer (PC) cell lines DU145 (CK8/14 ^+^, AR ^+^, PSA ^+^; RRID:CVCL_0105) and PC3 (CD44 ^+^, AR ^-^, PSA ^-^, CgA ^+^, NSE ^+^; RRID:CVCL_0035), derived from metastatic high-grade PCs, were obtained from ATCC (Manassas, VA, USA). WPMY-1 human prostatic fibroblasts (α-SMA ^+^, vimentin ^+^, fibronectin ^+^; RRID:CVCL_3814) were also purchased from ATCC. Human BM CD34 ^+^ hematopoietic stem/progenitor cells (HSPCs; Lonza, Cat. #2 M-101C) and mesenchymal stem cells (MSCs; Lonza, Cat. #PT-2501) were acquired from Lonza (Morrisville, NC, USA). All cells were cultured according to Supplementary Materials and Methods. Cell line identity was confirmed by STR profiling, and mycoplasma contamination was excluded by PCR testing.

### MTT assay

Cell viability and proliferation were assessed using the CellTiter 96 AQueous One Solution Cell Proliferation Assay (Cat. #G3582; Promega, Madison, WI, USA), according to manufacturer’s instructions. Experimental settings are described in the Supplementary Materials and Methods. Data are expressed as mean ± SD of three independent experiments, each performed in triplicate.

### Transfection with *IL27p28 *(*IL30*) expressing vector

For the overexpression of human *IL30* in DU145 and PC3 cells, we used the *IL27p28* Human Tagged ORF Clone (Cat. #RC209337L1; OriGene, Rockville, MD, USA) which was transfected in cancer cells using Lipofectamine 3000 Reagent (Cat. #L3000001; Thermo Fisher Scientific, Waltham, MA, USA) [18]. The expression of IL30 was confirmed by real-time RT-PCR and Western Blotting (WB) [18].

### CRISPR/Cas9-mediated IL30 gene knockout

For CRISPR mediated *IL30* gene deletion in DU145 and PC3 cell lines, we used two Trueguide Synthetic single guide (sg)RNAs, designed and synthesized by Thermofisher (Cat. #CRISPR947384_SGM, IL27p28, Human and Cat. #CRISPR947272_SGM, IL27p28, Human), and abrogation of *IL30* expression was validated by Western blotting [18].

### Human phosphorylation multi-pathway profiling array

To confirm that IL30 effects on prostatic fibroblasts are primarily mediated through the IL6Rα/gp130 receptor complex, phosphorylation signaling was evaluated using the Human Phosphorylation Multi-Pathway Profiling Array (Ray Biotech, Cat. #AAH-PPP-1–8), which enables the semi-quantitative detection of 55 phosphorylated proteins involved in MAPK, AKT, JAK/STAT, NF-κB, and TGFβ pathways. WPMY-1 cells were either left untreated or stimulated with recombinant IL30 (100 ng/ml; R&D Systems, Cat. #7430-ML), with or without prior neutralization using anti-IL6Rα (2.5 µg/ml; R&D Systems, Cat. #MAB227, RRID:AB_2127908) and anti-gp130 (2.5 µg/ml; R&D Systems, Cat. #AF-228-NA, RRID:AB_354411) antibodies. Neutralizing antibodies were applied for 1 h at 37 °C before IL30 stimulation for 30 min. Cells were then lysed, and samples processed according to the manufacturer’s instructions. Membranes were imaged using the Uvitec Alliance Q9 system, and signal intensities quantified with ImageJ Protein Array Analyzer (RRID:SCR_003070), with a ≥ 1.5-fold change corresponding to *p* < 0.01.

### ELISA

ELISA analysis was performed on protein extracts from PC and WPMY-1 cells, as described in the Supplementary Materials and Methods.

### PCR array and real-time RT-PCR

Real-time RT-PCR and PCR array were performed using the RT2 Profiler™ PCR Array Human Angiogenesis (Cat. #PAHS-024Z)**,** RT2 Profiler™ PCR Array Human ECM and Adhesion Molecules (Cat. #PAHS-013Z), RT2 Profiler™ PCR Array Human Prostate Cancer (Cat. #PAHS-135Z) (all from Qiagen, Hilden, Germany) and selected primers specific for PC driver and EMT genes, as reported in the Supplementary Materials and Methods. In the main figures, PCR array and real-time RT-PCR results are presented as bar graphs, for clarity and quantitative comparison. Complementary heatmap representations of the same datasets are provided in the Supplementary Information, to enable comprehensive visualization of gene expression patterns. A twofold change in gene expression was considered significant (*p* < 0.001), indicated by a dashed line in bar graphs. Only genes with a fold change > 2 are shown.

### Western blotting

WB was performed, on human PC and WPMY-1 cells, as described in the Supplementary Materials and Methods, using the primary Abs listed in the Table S3. All Western blot images are presented in their original, uncropped form in the Supplementary Information, to ensure transparency.

### PC cells and WPMY-1 2D co-cultures

To investigate tumor–stromal interactions, 2D co-culture experiments were performed by seeding WPMY-1 fibroblasts with either empty vector (EV)-transfected or IL30-overexpressing DU145 or PC3 cells at a 1:1 ratio in DMEM supplemented with 5% FCS. Co-cultures were maintained for 48 h under conditions previously optimized through proliferation assays to ensure the viability and balanced growth of both cell populations. Following incubation, fibroblasts and PC cells were separated by fluorescence-activated cell sorting (FACS) using a BD FACSAria Cell Sorter (RRID: SCR_018934). Cell populations were discriminated according to GFP and EpCAM expression: WPMY-1 fibroblasts were GFP ^−^/EpCAM ^−^, whereas PC cells were GFP ^+^/EpCAM ^+^. After sorting, each sample was equally divided for subsequent RNA and protein extraction analyses.

### 3D PC spheroids

EV-transfected, IL30-overexpressing, and IL30 knockout (IL30KO) DU145 or PC3 cells were cultured either alone or with WPMY-1 cells (1:1 ratio) in 96-well U-bottom ultra-low-attachment plates (Cat. #174925, Thermo Fisher Scientific, Waltham, MA, USA) for 24 h to generate spheroids. Formed spheroids were transferred using sterile transfer pipettes (Cat. #204-1S, Thermo Fisher Scientific) into 24-well ultra-low-attachment plates (Cat. #174930, Thermo Fisher Scientific) and maintained for an additional 48 h. Spheroids were subsequently loaded into the 24-well compartment of the 2-OC system (HUMIMIC Chip2; TissUse, Berlin, Germany). Experimental procedures ensuring reproducibility and reduced variability are described in the Supplementary Materials and Methods.

### 3D models of BM niche

3D models of the BM were prepared according to TissUse’s protocols. MSCs were cultured in T-175 flasks using the Mesencult-ACF Plus, for 7–10 days (Cat. #05445, STEMCELL Technologies, Vancouver, Canada). They were then seeded on Sponceram cylinders (TissUse, Berlin, Germany), made of hydroxyapatite-coated zirconium oxide ceramic scaffold and cultured, for 7–10 days, in Mesencult-ACF Plus Medium (Cat. #05445, STEMCELL Technologies, Vancouver, Canada). On day 1 of the 2-OC experiment, the MSCs containing scaffolds were seeded with BM CD34^+^ HSPCs, and inserted into the 96-well compartment of the 2-OC.

### Incorporation of PC and BM scaffold into 2-OC device

The 2-OC used consisted of a 24-well compartment connected, in circuit, by microfluidic channels to a 96-well compartment. On each chip, two circuits were present. Twenty PC spheroids, containing 10 000 cells, were placed inside an 8 mm culture plate insert (Cat. #PI8P01250, Merck, Burlington, MA, USA) present in the 24-well compartments of each circuit. BM scaffolds were loaded into the 96-well compartment of each circuit.

### Laser scanning confocal analyses

3D PC spheroids were generated by seeding either WT- EV- or IL30-DU145 or IL30-PC3 cells, labelled with the LuminiCell Tracker 540-Cell Labeling Kit (green fluorescence; Cat. #SCT010, Merck, Burlington, MA, USA). PC cells were cultured with or without WPMY-1 stromal fibroblasts stained with the LuminiCell Tracker 670-Cell Labeling Kit (red fluorescence; Cat. #SCT011, Merck), according to manufacturer’s protocol. At the end of the 2-OC experiment, PC spheroids were visualized using a Zeiss LSM 800 confocal microscope, with Airyscan (RRID: SCR_015963). Images were evaluated and processed using the Zeiss ZEN Microscopy Software (RRID:SCR_013672) (Zeiss, Oberkochen, Germany).

### Flow cytometry

Cells were harvested and mechanically dissociated into a single-cell suspension. After centrifugation, cells were resuspended in PBS and incubated for 30 min at 4 °C with the following antibodies: anti-human CD126 phycoerythrin-conjugated (BD Biosciences, Cat. #551850, RRID:AB_394271), anti-human CD130 FITC-conjugated (Abcam, Cat. #ab47218, RRID:AB_868803), anti-human IL30 eFluor™ 660-conjugated (Thermo Fisher Scientific, Cat. #50-8277-42, RRID:AB_11149127), and anti-human EpCAM APC-conjugated (Miltenyi Biotec, Cat. #130-111-000). Acquisition was performed using a BD FACSVerse™ flow cytometer (BD Biosciences), and data were analyzed with FlowJo software (RRID:SCR_008520). Dead cells were excluded by 7-AAD staining. Non-specific fluorescence was assessed using fluorescence minus one (FMO) controls, while compensation was performed using CompBeads and single-stained controls. Proliferation, apoptosis of PC and WPMY-1 cells, and migration of GFP ^+^ PC cells into BM scaffolds were quantified as described in the Supplementary Materials and Methods (Fig. S16–S17). All experiments were performed in triplicate.

### Endothelial tube formation assay

HUVECs (100,000 cells/well) were seeded onto 24-well plates pre-coated with Matrigel (Cat. #356231; Corning, Amsterdam, NL) according to the manufacturer’s instructions. Cells were cultured for 18 h at 37 °C in conditioned medium (CM) collected from WPMY-1 fibroblasts, either untreated or stimulated with recombinant IL30 (30 or 50 ng/mL). Tube formation was visualized using a Thermo Fisher Scientific EVOS M5000 Imaging System (RRID:SCR_023650) with a 4 × objective in pseudo-phase contrast. For each well, 5–6 randomly selected fields were acquired for analysis. Images were quantified using the “Angiogenesis Analyzer” plugin in ImageJ (RRID:SCR_003070), assessing number of segments, meshes, nodes, and junctions. Nodes were defined as branching points, junctions as clusters of adjacent nodes, segments as structures between two junctions, and meshes as interconnected segment networks [65].

### Wound healing assay

Prostate cancer (PC) cells (DU145 and PC3) were seeded into 24-well plates, at a density of 1 × 10^5^ cells/well and grown to confluence. After 24 h, a linear scratch was made in the centre of each well using a sterile 200 µl pipette tip. Then, the wells were washed with PBS and, subsequently, serum-free medium or serum-free CM derived from WPMY-1 cells cultured alone, WPMY-1 supplemented with rIL30 (50 ng/ml) or WPMY-1 in co-culture with EV and/or IL30 PC, was added.

Images were acquired immediately after scratching (time 0) and after 8 h and 24 h using an EVOS M5000 Imaging System (Cat. #AMF5000; ThermoFisher Scientific, Waltham, MA, USA). The migration rate (R_M_) was calculated using the formula *R*_*M*_ = *(Wi − Wf)/t*, where Wi represents the initial scratch width, Wf the final scratch width, and *t* the duration of the experiment.

### Prostate cancer xenograft samples

NSG male mice (RRID:IMSR_JAX:005557) were obtained from Charles River Laboratories and housed under high-barrier conditions in accordance with The Jackson Laboratory guidelines at the animal facility of the Center for Advanced Studies and Technology, Chieti, Italy. To assess in vivo the effects of IL30 overexpression in PC cells on their crosstalk with WPMY-1 cells, particularly regarding their expression of ECM components, angiogenesis and PC driver genes, six cohorts of 8-week-old NSG mice (n = 15 per group) were subcutaneously inoculated with co-cultures of WPMY-1 cells and WT, EV, or IL30-overexpressing DU145 or PC3 cells at a 2:1 ratio (1 × 10^6^ total cells per mouse, suspended in PBS). Tumor growth was monitored as reported in the Supplementary Materials and Methods.

Animal procedures were performed in accordance with the European Community and ARRIVE guidelines and were approved by the Institutional Animal Care Committee of “G. d’Annunzio” University and by the Italian Ministry of Health (Authorization n. 409/2025-PR).

### Histology, histochemistry, immunohistochemistry and morphometric analyses

For histological analysis, tissue samples were fixed in 4% formalin, paraffin-embedded, sectioned at 4 μm, and stained with hematoxylin and eosin. Masson’s trichrome staining was additionally performed to distinguish PC cells from WPMY-1–enriched connective tissue, as detailed in the Supplementary Materials and Methods. Immunostainings were carried out as previously described [52], using antibodies listed in Table S4. Expression of extracellular matrix components, angiogenic markers, and key PC driver genes was evaluated using a Leica DM2500 microscope equipped with a Leica DFC camera and QWin image analysis software (Leica QWin, RRID:SCR_018940).

### Bioinformatic analyses

Gene expression data from the *Metastatic Prostate Adenocarcinoma (SU2C/PCF Dream Team)* database (n = 201) were downloaded from the cBioPortal platform (RRID:SCR_014555). For each sample, Z-scores of gene expression levels were calculated relative to the mean expression level of all samples in the dataset, to minimize the impact of outliers and technical variability. Correlations between *IL30* expression and that of PC driver genes were assessed using the Pearson correlation coefficient.

### Statistical analysis

All experiments were performed in biological triplicate unless otherwise specified. Between-group differences were assessed using one-way or two-way ANOVA models, including interaction terms when appropriate, followed by post-hoc pairwise comparisons with multiple-testing correction.

Data in bar graphs are presented as mean ± SD and black dots overlaid on the bars represent individual replicates. All experiments were performed in triplicate and results from wild type PC cells were comparable to those from EV-PC cells.

All statistical tests were performed at an α level of 0.05, and results are presented as mean ± standard deviation. All analyses were conducted using GraphPad Prism (RRID:SCR_002798).

## Supplementary Information

Supplementary Material 1.

## Data Availability

All raw data generated in this study are available upon request from the corresponding author. Expression profile data from the *Metastatic Prostate Adenocarcinoma (SU2C/PCF Dream Team)* database (n = 201) were downloaded from the cBioPortal platform (RRID:SCR_014555).
