# Supplementary material for: Prostatic fibroblast reprogramming by Interleukin-30 activates prostate cancer metastasis programs
Source: Mol Biomed. 2026 Jun 24;7:96. doi: 10.1186/s43556-026-00482-9 (PMC13294413; doi:10.1186/s43556-026-00482-9)
Supplement: Supplementary file 1 — Supplementary Material 1. [file 43556_2026_482_MOESM1_ESM.pdf]

Prostatic fibroblast reprogramming by Interleukin-30 activates prostate cancer metastasis programs

Stefania Livia Ciummo[1,2], Carlo Sorrentino[1,2], Simona Marchetti[1,2], Cristiano Fieni[1,2], Paola Lanuti[1], Emma Di Carlo[1,2*]

1. Department of Medicine and Sciences of Aging, "G. d'Annunzio" University" of Chieti-Pescara, Via dei Vestini, Chieti, 66100, Italy.
2. Anatomic Pathology and Immuno-Oncology Unit, Center for Advanced Studies and Technology (CAST), "G. d'Annunzio" University of Chieti-Pescara, Via L. Polacchi 11, Chieti, 66100, Italy

***Corresponding author:** Prof. Emma Di Carlo, Anatomic Pathology and Immuno-Oncology Unit, Center for Advanced Studies and Technology (CAST), "G. d'Annunzio" University, Via L. Polacchi 11, Chieti, 66100, Italy. Phone: +39 0871 541540; e-mail: edicarlo@unich.it.

SUPPLEMENTARY MATERIALS AND METHODS

Cell cultures

Human prostate cancer (PC) cell line DU145 was cultured in RPMI 1640 (#15-040-CV, Corning, Manassas, VA, USA) with 10% FCS (Seromed-Biochrom KG, Berlin, Germany). Human PC cell line PC3 was cultured in F12 Ham's (#10-080-CV, Corning, Manassas, VA, USA) with 10% FCS (Seromed-Biochrom KG, Berlin, Germany). Human prostatic fibroblasts, WPMY-1 cells were cultivated in DMEM (#10-013-CV, Corning, Manassas, VA, USA) and 5% FCS (Seromed-Biochrom KG, Berlin, Germany). Human bone marrow $CD34^{+}$ hematopoietic stem and progenitor cells (HSPCs) were cultured in StemSpan SFEM II medium (#09655, STEMCELL Technologies, Vancouver, Canada) supplemented with StemSpan CD34+ Expansion Supplement (#02691, STEMCELL Technologies). Human bone Marrow derived Mesenchymal Stem Cells (MSCs), isolated from normal adult human bone marrow, were cultured in Mesencult-ACF Plus Medium Kit (#05445, STEMCELL Technologies, Vancouver, Canada).

MTT assay

To assay the effects of IL30 on the proliferation of fibroblast cells, WPMY-1 cells were seeded in 96-well plates and were either incubated for 48h with different concentrations (from 0 ng/ml to 50 ng/ml) of recombinant IL30 (rIL30; #7430-ML, R&D Systems, Minneapolis, MN, USA), or incubated with 50 ng/ml of rIL30, in the presence or absence of neutralizing anti-CD130 (R and D Systems Cat# AF-228-NA, RRID:AB_354411) and/or anti-CD126 Abs (R and D Systems Cat# MAB227, RRID:AB_2127908) at concentrations of 0.5, 1.0, 2.5 and 5.0 µg/ml.

To assay the effects of WPMY-1 CM on the proliferation of endothelial cells, HUVECs were incubated with WPMY-1 culture medium or with WPMY-1 CM or with CM obtained from WPMY-1 cells treated with 30 ng/ml rIL30.

Absorbance was measured using a SpectraMax 190 microplate reader (RRID: SCR_018932; Molecular Devices, San Jose, CA, USA), and cell proliferation was calculated relative to untreated controls. Data are expressed as mean ± SD of three independent experiments, each performed in triplicate.

ELISA

WPMY-1 cells were seeded in T-25 flasks (100,000 cells per flask) and treated with 50 ng/mL recombinant IL30 (rIL30) every two days or left untreated. After 3, 5, and 7 days, both treated and untreated cells were collected, lysed, and analyzed using the Cancer Associated Fibroblast Marker ELISA Sampler Kit (#SKFI0037, Assay Genie, Dublin, Ireland), following the manufacturer's instructions. This kit enables the detection and quantification of key markers associated with CAFs, including FAP, PDGFRα, PDGFRβ, Vimentin, ACTA2, and S100A4.

PCR array and real-time RT-PCR

Primers for the following genes were designed and synthesized by Sigma-Aldrich Corporation (St. Louis, MO, USA): CD44 Forward 5′-AGGAACAGTGGTTTGGCAAC-3′ and CD44 Reverse 5′-CGAATGGGAGTCTTCTCTGG-3′; KISS1 Forward 5′-GAACTACAACTGGAACTCCTT-3′ and KISS1 Reverse 5′-ATGCTCTGACTCCTTTGG-3′; MMP2 Forward 5′- AGCGAGTGGATGCCGCCTTTAA-3′ and MMP2 Reverse 5′-CATTCCAGGCATCTGCGATGAG-3′; MMP9 Forward 5′-GCCACTACTGTGCCTTTGAGTC-3′ and MMP9 Reverse 5′-CCCTCAGAGAATCGCCAGTACT-3′; NM23 Forward 5′- GTGAGTTCTCCCTGTACA-3′ and NM23 Reverse 5′-AGCAATGCAACAATATGAAGTA-3′; SNAI1 Forward 5′-CCTCTTCTCTCCATACCT-3′ and SNAI1 Reverse 5′-TTCATCAAAGTCCTGTGGG-3′; SNAI2 Forward 5′-TGTCATACCACAACCAGAGA-3′ and SNAI2 Reverse 5′-

CTTGGAGGAGGTGTCAGAT-3′; TWIST1 Forward 5′-CGGAGACCTAGATGTCATT-3′ and TWIST1 Reverse 5′-CTGTCTCGCTTTCTCTTTT-3′; VEGFA Forward 5′-TTGCCTTGCTGCTCTACCTCCA-3′ and VEGFA Reverse 5′-GATGGCAGTAGCTGCGCTGATA-3′; ZEB1 Forward 5′- CCAACAGACCAGACAGT-3′ and ZEB1 Reverse 5′-TGACTCGCATTCATCATT-3′; ZEB2 Forward 5′-CGGAGACTTCAAGGTATAATCTATC-3′ and ZEB2 Reverse 5′-GTTACGCCTCTTCTAATGACAT-3′.

Primers for the following genes were purchased from Qiagen (Hilden, Germany): BMP7 (#QT00068936); MET (#QT00023408); MYC (#QT00035406); NOTCH1 (#QT01005109); STAT3 (#QT00068754); TGFB1 (#QT00000728).

PCR reactions were performed with the Quantifast SYBR Green PCR Kit (#204054, Qiagen) on a MiniOpticon System (#CFB-3120, Bio-Rad, Hercules, CA, USA). Gene expression was normalized to the median of housekeeping genes, and relative expression was calculated using the ΔΔCt method. Melting curve analysis confirmed specificity, and amplification efficiency was verified using serial cDNA dilutions. No-template controls were included.

Real-time PCR cycling conditions: Real-time PCR reactions were performed in a final volume of 20 μL. The amplification protocol included an initial denaturation step at 95 °C for 5 minutes, followed by 39 amplification cycles. Each cycle consisted of denaturation at 95 °C for 10 seconds and annealing/extension at 60 °C for 30 seconds, during which fluorescence data were collected.

At the end of the amplification phase, a melt curve analysis was performed to assess the specificity of the amplified products. The melting protocol consisted of an initial step at 95 °C for 10 seconds, followed by cooling to 65 °C for 30 seconds and a final heating step at 95 °C for 30 seconds. Melt curve data were acquired with a temperature resolution of 0.5 °C and a soaking time of 5 seconds per increment.

<u>*PCR Array cycling conditions*</u>: PCR Array reactions were carried out in a final volume of 20 µL. The cycling program included an initial activation step at 95 °C for 10 minutes, followed by 40 amplification cycles. Each cycle comprised a denaturation step at 95 °C for 10 seconds without fluorescence acquisition and an annealing/extension step at 60 °C for 30 seconds, during which fluorescence was acquired on the green channel (Cycling A).

Melt curve analysis was performed by gradually increasing the temperature from 50 °C to 99 °C, with 1 °C increments per step. A pre-melt conditioning period of 90 seconds was applied at the first temperature step, followed by a 5-second hold for each subsequent step. Fluorescence data were acquired on the green channel (Melt A). Automatic gain optimization was enabled for all wells, and the gain providing the highest fluorescence value below 95 was selected.

Western Blotting

For total protein extraction, cells were collected by centrifugation and lysed with ice cold RIPA Lysis buffer (#89900, Thermo Fisher Scientific), supplemented with Protease and Phosphatase Inhibitors Cocktail (#78440, Thermo Fisher Scientific). Total proteins were then quantified using the Bradford assay. Protein samples were loaded on Mini-PROTEAN TGX Gels 4-20% (#4561094; Bio-Rad) and transferred on Immuno-Blot PVDF Membranes (#1620177; Bio-Rad, Hercules, CA, USA), using the Mini Trans-Blot Cell apparatus (#1703930, Bio-Rad). Membranes were then blocked with 5% milk (#ERMBD282, Sigma-Aldrich) in TBST and probed with the primary Abs listed in Supplementary Table 1, followed by incubation with the appropriate HRP-conjugated secondary Ab: goat anti-rabbit IgG (whole molecule)-peroxidase (Sigma-Aldrich Cat# A0545, RRID:AB_257896) or rabbit anti-mouse IgG (whole molecule)-peroxidase (Sigma-Aldrich Cat# A9044, RRID:AB_258431). Mouse anti-ACTB Ab (Sigma-Aldrich Cat# A2228, RRID:AB_476697) was used as loading control.

Signal detection was performed using the Pierce™ ECL Western Blotting Substrate (#32106; Thermo Fisher Scientific), according to the manufacturer's instructions.

3D PC spheroids

The experimental setup was designed to ensure reproducibility while minimizing experimental variability. PC spheroids were cultured in a 1:1 (v/v) co-culture medium consisting of StemSpan™-AOF medium supplemented with 10 ng/mL thrombopoietin (TPO; #300-18, Thermo Fisher Scientific, Waltham, MA, USA) and 25 ng/mL Fms-related tyrosine kinase 3 ligand (FLT3L; #300-19, Thermo Fisher Scientific, Waltham, MA, USA), combined with DMEM (#10-013-CV, Corning, Manassas, VA, USA) supplemented with 5% fetal calf serum (FCS; Seromed-Biochrom KG, Berlin, Germany). The medium was further supplemented with 1% Antibiotic-Antimycotic (100X; Gibco, Thermo Fisher Scientific; Cat# 15240062). The cell seeding densities, ratios, and culture conditions used to generate 3D PC spheroids were optimized through proliferation assays. These assays identified conditions that supported the growth and viability of all cell types within both the PC spheroids and the BM scaffold model.
To minimize variability and ensure consistency across the 24-well compartments of the 2-OC system, twenty PC spheroids were seeded per well for each experimental condition, with each spheroid containing 10,000 cells, thereby ensuring identical initial cell numbers. This setup is consistent with our previous study (Fieni et al., *Molecular Therapy*, 2024).

Flow cytometry

To assess the migration of PC cells to the bone marrow, the BM scaffolds were washed with a solution of PBE (PBS without calcium and magnesium, containing 0.5 mM EDTA and 0.6% BSA). Then, they were incubated in the same solution, for 15 min at room temperature, and subsequently incubated with TrypLE™ Express Enzyme (#12605010, Thermofisher

Scientific), for another 15 min. Cell suspensions from both the PBE and TrypLE™ incubations were collected, pooled, and centrifuged. The resulting cell pellets were resuspended in staining buffer and subjected to flow cytometric analysis.

The gating strategy used to quantify the migration of GFP^+ PC cells was as follows (Supplementary Fig. S16):

Cells were first gated based on forward scatter (FSC) and side scatter (SSC) parameters to exclude debris and non-cellular events. As negative controls, BM scaffolds without seeded cells were analyzed to account for potential background debris.

Within the gated cell population ("Cells"), GFP^+ PC cells were identified using SSC-A and the FITC channel. Gating thresholds were established using BM scaffolds seeded only with hMSCs and $CD34^+$ cells, which were used to define the GFP^- population.

Viability and apoptosis of both PC and WPMY-1 cells in 2D co-cultures and 2-organ-on-chip (2-OC) experiments were assessed by Ki67 and Annexin V staining. Briefly, 2D co-cultures were harvested and mechanically dissociated into a single-cell suspension, whereas tumor spheroids were collected from the 24-well compartment of the 2-OC chip and dissociated into a single-cell suspension using Accumax Cell Aggregate Dissociation Medium (#00-4666-56; Thermo Fisher Scientific).

Cells were resuspended in BD Pharmingen™ Stain Buffer (BSA) (Cat# 554657; BD Biosciences) and stained with anti-human APC-conjugated EpCAM antibody (Miltenyi Biotec, Cat# 130-111-000) for 30 minutes at 4 °C. After staining, samples were centrifuged, washed, resuspended in PBS, and split into two aliquots. One aliquot was used for Ki67 staining and the other for Annexin V staining.

For Ki67 staining, cells were fixed and permeabilized using the BD Cytofix/Cytoperm™ Fixation/Permeabilization Kit (#554714; BD Biosciences, Franklin Lakes, NJ, USA) according to the manufacturer's instructions, resuspended in staining buffer, and incubated

for 30 minutes at 4 °C with anti-Ki67 antibody (Thermo Fisher Scientific, Cat# 404-5698-82; RRID: AB_2925532).

Apoptosis was assessed using the Pacific Blue™ Annexin V/SYTOX™ AADvanced™ Apoptosis Kit (#A35136; Thermo Fisher Scientific) according to the manufacturer's protocol. The gating strategy used to identify Ki67- and Annexin V-positive and negative cells was as follows (Supplementary Fig. S17):

1. Cells were initially gated based on forward scatter (FSC) and side scatter (SSC) parameters to exclude debris and non-cellular events.
2. Within the gated "Cells" population, WPMY-1 and PC cell populations were identified based on EpCAM (APC channel) and GFP (FITC channel) expression: WPMY-1 cells were negative for both EpCAM and GFP, whereas PC cells were positive for both markers. Gating thresholds were established using EpCAM-stained and unstained WPMY-1-only and PC-cell-only control samples.
3. Ki67 (BV421) and Annexin V (Pacific Blue) signals were analyzed separately in PC and WPMY-1 cells cultured alone or in co-culture. Thresholds for Ki67- and/or Annexin V-negative events were defined using unstained controls.

Data acquisition was performed using a BD FACSVerse™ Flow Cytometer (BD Biosciences, Franklin Lakes, NJ, USA), and data analysis was conducted using FlowJo software (RRID: SCR_008520).

Prostate cancer xenograft samples

Tumors were measured with calipers every three days starting from the time they became palpable, by investigators blinded to the experimental groups. Mice were sacrificed when the tumor reached 700 mm^3, since at this size there are still no important necrotic phenomena that can invalidate the immunohistochemical examination. An overall sample

size of 15 mice per group allowed the detection of a statistically significant difference between the three groups, with an 80% power, at a 0.05 significance level (G*Power, RRID:SCR_013726).

Histology, histochemistry, immunohistochemistry and morphometric analyses

Masson's trichrome staining was performed on formalin-fixed, paraffin-embedded tissue sections, using the Masson's trichrome kit (#04-010802, Bio-Optica, Milano, Italy) according to manufacturer's instructions. Briefly, after deparaffinization, sections were incubated in Bouin's solution at 56 °C for 1 hr. Then, the slides were sequentially stained with Weigert's iron hematoxylin for nuclei (10 min), Ponceau B for cytoplasm (4 min), phosphomolybdic-phosphotungstic acid (10 min) and aniline blue (10 min) for collagen visualization. Sections were then washed in 1% acetic acid, cleared and mounted.

Morphometric analyses were performed, on single immunostained sections, using a Leica DM2500 microscope equipped with a Leica DFC camera and QWin image analysis software (Leica QWin, RRID:SCR_018940). Analyses were carried out, at ×400 magnification, on 85,431.59 μm^2 fields. The image processing workflow allowed the morphometric analysis to take into account both the extent and the intensity of the staining, and consisted of the following steps: (1) digital image acquisition; (2) conversion of RGB (true color) images to binary (black and white) format; (3) background noise reduction; (4) automated counting of positive cells or measurement of positively stained area; and (5) assessment of the optical density of the stained areas. A total of 6–8 high-power fields were analyzed per section, and five sections were evaluated for each sample. Results were expressed as mean percentage of positive cells/number of total cells ± SD (NOTCH1, SNAI2) or mean percentage of positively stained area/total area of viable tissue within the examined field ± SD (ANG, HAS1, IL6, JAG1, TGFB1, VEGFA).

Table S1. Morphometric analysis of the expression of ECM and angiogenesis related genes in tumor xenografts developed after co-injection of prostatic WPMY-1 fibroblasts with IL30-overexpressing PC cells or EV-PC cells, in NSG mice.

Genes	WPMY-1 + EV-DU145	WPMY-1 + IL30-DU145	WPMY-1 + EV-PC3	WPMY-1 + IL30-PC3	Student's *t*-test (*p*)
HAS1*	12 ± 4%	54 ± 10%	11 ± 2%	56 ± 12%	<0.01
TGFB1*	35 ± 9%	66 ± 15%	42 ± 9%	75 ± 16%	<0.01
JAG1*	9 ± 2%	32 ± 9%	8 ± 3%	30 ± 9%	<0.01
ANG*	13 ± 2%	60 ± 14%	12 ± 3%	58 ± 14%	<0.01

* Results are expressed as mean percentage of positively stained area/total area of viable tissue within the examined field ± SD, evaluated as described in Supplementary Materials and Methods. Results obtained in tumors developed after co-injection of WPMY-1 with WT-PC cells were comparable to those obtained in tumors developed after co-injection of WPMY-1 with EV-PC cells.

Table S2. Morphometric analysis of PC driver and EMT related genes in tumor xenografts developed after injection of IL30-overexpressing PC cells with or without prostatic WPMY-1 fibroblasts, in NSG mice.

**Genes**	**IL30-DU145**	**IL30-DU145 + WPMY-1**	**IL30-PC3**	**IL30-PC3 + WPMY-1**	**Student's *t*-test (*p*)**
IL6*	39 ± 8%	68 ± 12%	46 ± 9%	74 ± 16%	<0.01
VEGFA*	47 ± 12%	87 ± 17%	54 ± 13%	81 ± 15%	<0.01
NOTCH1†	50 ± 11%	73 ± 15%	56 ± 9%	77 ± 14%	<0.01
SNAI2†	43 ± 8%	62 ± 11%	48 ± 9%	70 ± 11%	<0.01

* Results are expressed as mean percentage of positively stained area/total area of viable tissue within the examined field ± SD, evaluated as described in Supplementary Materials and Methods.
† Results are expressed as mean percentage of positive cells/number of total cells ± SD, evaluated as described in Supplementary Materials and Methods.

Table S3. Antibodies used in Western blotting

Protein	Code	Dilution	RRID*	Source
CCL11	ab133604	1:1000	Not available	Abcam, Cambridge, UK
IL30 (IL27p28)	ab118910	1:1000	AB_10898806	“
IL6	ab9324	1:2000	AB_307175	“
CNTN1	15413	1:1000	AB_2798740	Cell Signaling Technology, Danvers, MA, USA
EGR3	2559	1:1000	AB_11142006	“
FOXO1	2880	1:1000	AB_2106495	“
ITGAV	60896	1:1000	AB_2753190	“
MMP3	14351	1:1000	AB_2798459	“
NKX3.1	83700	1:1000	AB_2800027	“
TIMP3	5673	1:1000	AB_10694530	“
VCAM1	13662	1:1000	AB_2798286	“
LGALS4	GTX114527	1:1000	AB_2885342	GeneTex, Irvine, CA, USA
MMP13	GTX100665	1:1000	AB_2037446	“
PTGS2	GTX100656	1:1000	AB_1951531	“
VEGFA	AF-293-NA	1:1000	AB_354450	R&D Systems, Minneapolis, MN, USA
CCL2	710002	1:1000	AB_2532516	Thermofisher Scientific, Waltham, MA, USA
DKK3	PA5-102626	1:1000	AB_2852023	“
HAS1	PA5-95599	1:1000	AB_2807401	“
MMP12	MA5-32011	1:1000	AB_2809305	“
SPP1	PA5-34579	1:1000	AB_2551931	“
TGFA	PA5-120044	1:1000	AB_2913616	“
TGFB1	MA5-15065	1:1000	AB_10984297	“

*RRID: Research Resource Identifier.

Table S4. Antibodies used in immunohistochemistry

Protein	Code	RRID*	Dilution	Source
IL6	ab9324	AB_307175	1:200	Abcam, Cambridge, UK
VEGFA	ab1316	AB_299738	1:100	“
PTGS2	GTX100656	AB_1951531	1:100	GeneTex, Irvine, CA, USA
NOTCH1	TA500078	AB_2282907	1:50	OriGene Technologies, Rockville, MD, USA
SNAI2	TA800167	AB_2625278	1:150	“
ANG	NBP2-41185	AB_3302052	1:50	R&D Systems, Minneapolis, MN, USA
JAG1	AF1277	AB_354713	1:10	“
TGFB1	sc-146	AB_632486	1:50	Santa Cruz Biotechnology, Dallas, TX, USA
HAS1	PA5-95599	AB_2807401	1:500	Thermofisher Scientific, Waltham, MA, USA

*RRID: Research Resource Identifier.

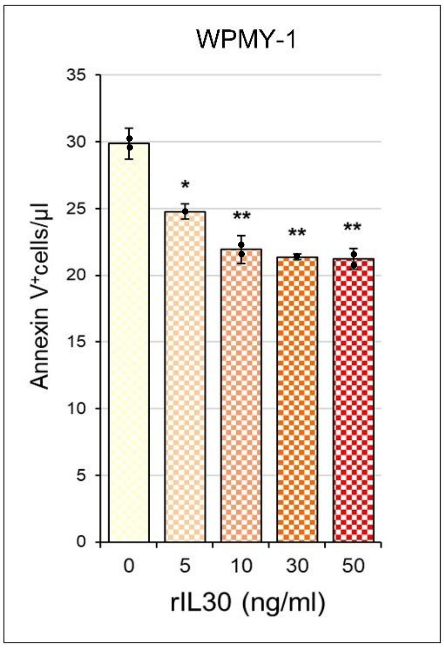

Fig. S1 Flow-cytometric analysis of apoptotic events (Annexin V$^+$ cells/µl) in WPMY-1 cells untreated (0) or treated with rIL30 (5–50 ng/ml). ANOVA: p<0.0001. *p<0.01, Tukey HSD Test compared with 0 ng/ml. **p<0.05, Tukey HSD Test compared with 0 and 5 ng/ml. Experiments were performed in triplicate and results are expressed as mean ± SD. Black dots overlaid on the bars represent individual replicates.

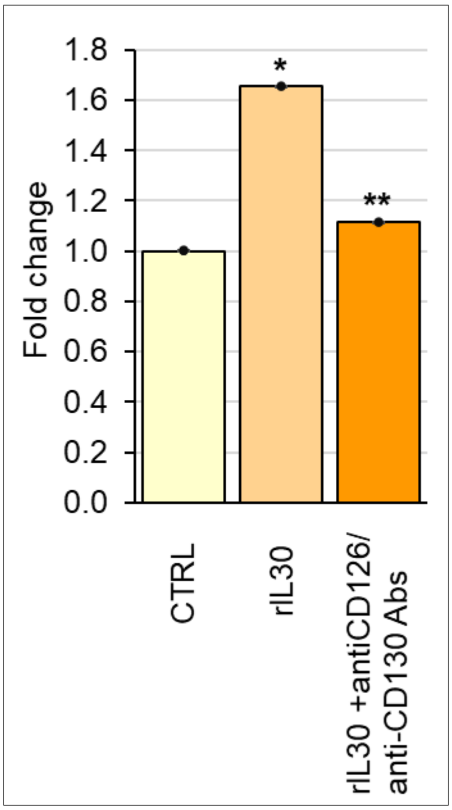

Fig. S2 MTT assay of WPMY-1 cells, 48 hours after treatment with rIL30 (50 ng/ml) with or without anti-CD126 and anti-CD130 Abs (1 µg/ml). CTRL: untreated WPMY-1 cells. ANOVA: $p<0.0001$. *$p<0.01$, Tukey HSD test compared with CTRL. **$p<0.01$, Tukey HSD test compared with CTRL and rIL30. Results are expressed as mean ± SD and black dots overlaid on the bars represent individual replicates. Experiments were performed in triplicate.

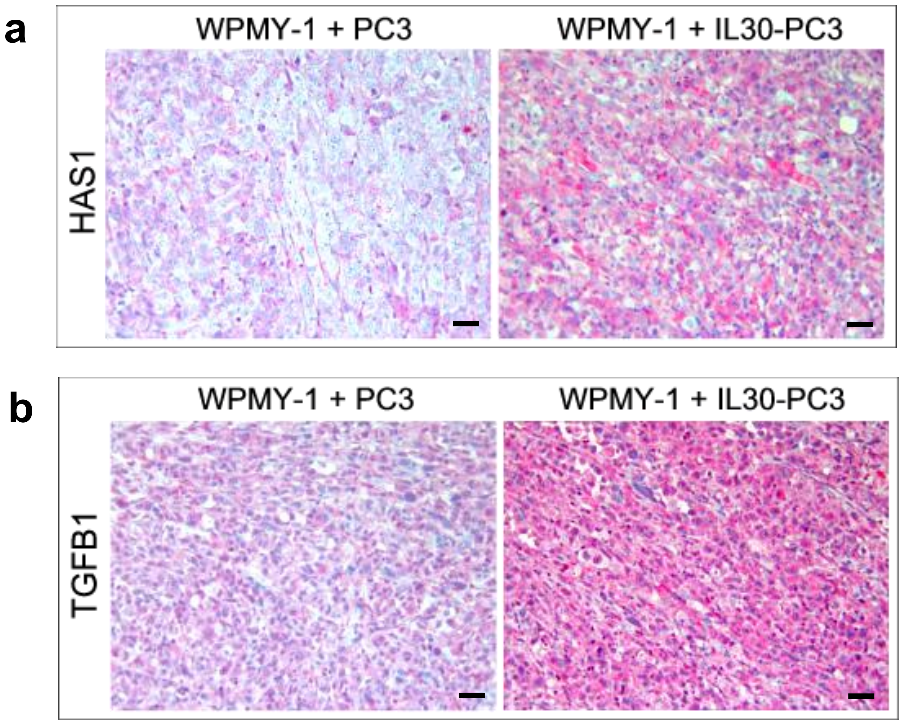

Fig. S3 Immunohistochemical staining of tumor xenografts revealed increased expression of HAS1 (a) and TGFB1 (b), primarily in prostatic WPMY-1 fibroblasts, when co-injected subcutaneously with IL30-overexpressing PC3 cells (right panel), compared with WT (or EV) PC3 cells (left panel), in NSG mice. Results from WT-PC cells were comparable to those from EV-PC cells. Magnification: ×200. Scale bars: 40 μm.

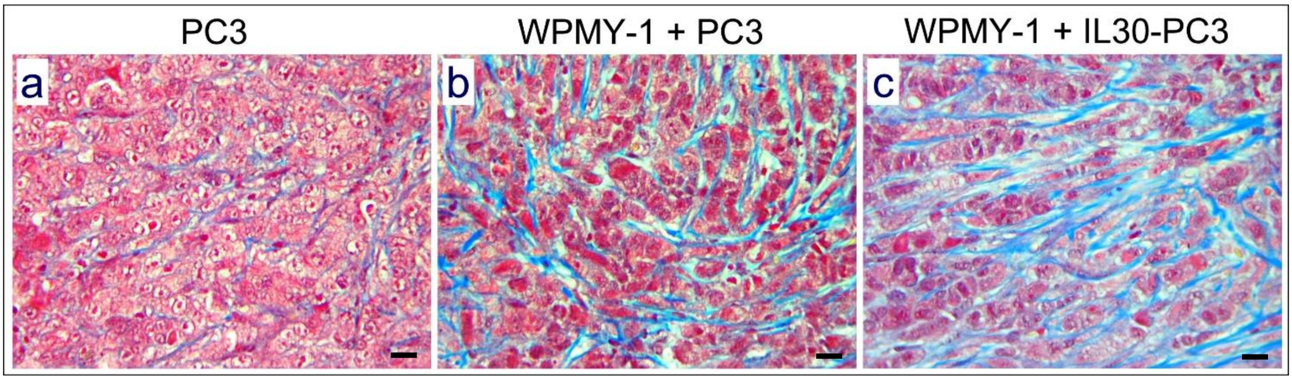

Fig. S4 Histochemical staining using Masson's trichrome, highlights the tumor's extracellular matrix in blue, while the tumor cells are red. Compared to the tumor developed following the implantation of PC cells only, in NSG mice (**a**), the tumor developed following the co-inoculation of PC cells with prostatic fibroblasts, WPMY-1, presents a greater stromal and ECM component (**b**)**.** When fibroblasts were co-injected with IL30-overexpressing PC cells the resulting tumor has a more robust stroma and a higher extracellular matrix content (**c**). Magnification: ×400. Scale bars: 20 µm.

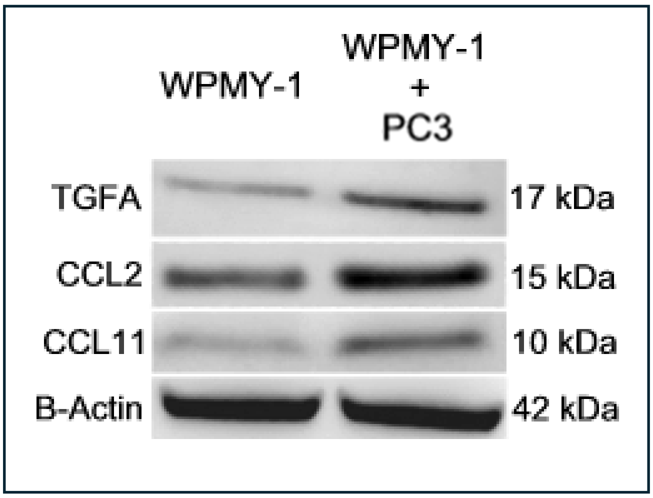

Fig. S5 Western blot analyses of TGFA, CCL2, and CCL11 protein expression in WPMY-1 cells cocultured or not with WT-PC3 cells.

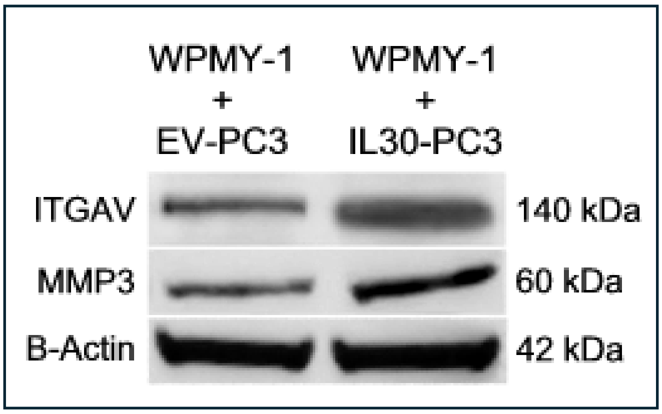

Fig. S6 Western blot analyses of ITGAV and MMP3 protein expression in WPMY-1 cells cocultured with EV- or IL30-PC3 cells. Results of cocultures with WT-PC3 cells were comparable to those from coculture with EV-PC3 cells.

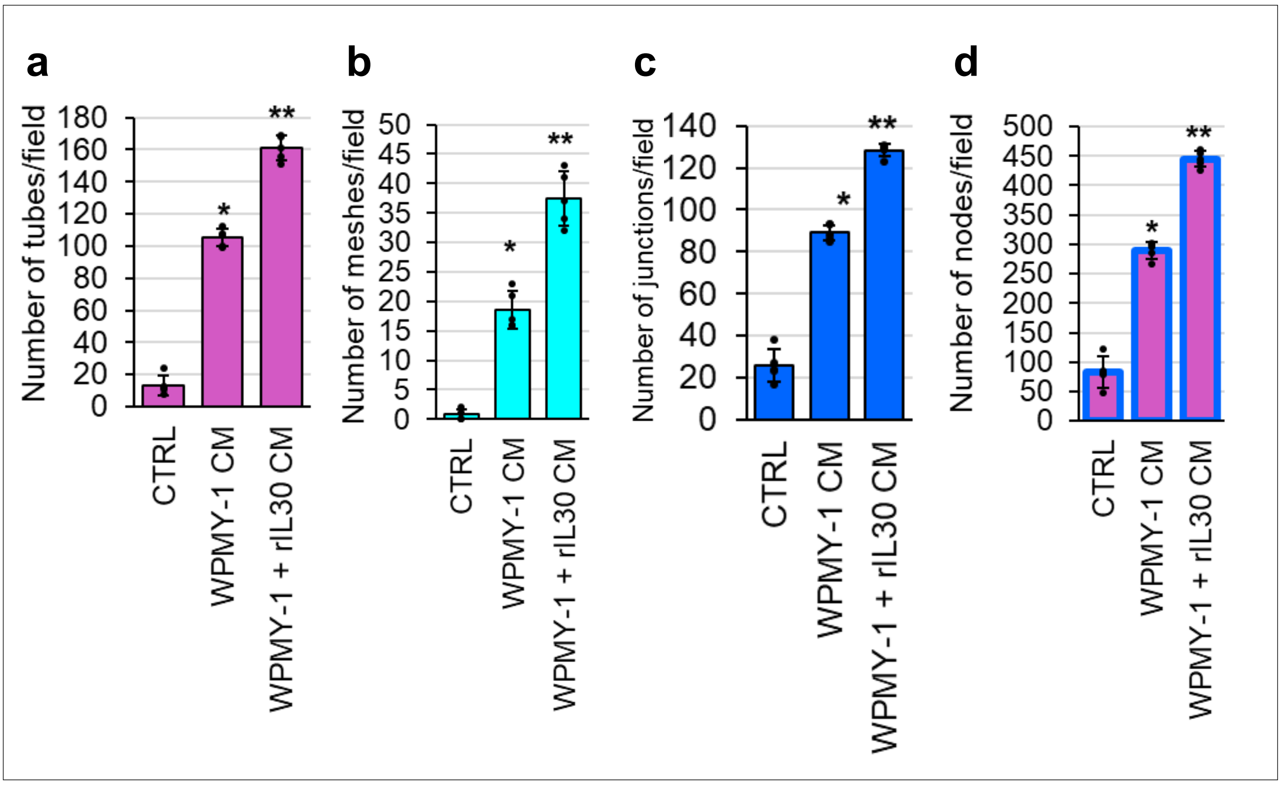

Fig. S7 Mean number of (**a**) endothelial tubes, (**b**) capillary meshes, (**c**) tube junctions, (**d**) vascular nodes, formed by HUVEC cultured, on Matrigel-coated wells, with WPMY-1 cells medium (CTRL), WPMY-1 conditioned medium (CM) or CM obtained from WPMY-1 cells treated with 30 ng/ml rIL30. Results from HUVEC cultured with WPMY-1 CM obtained from WPMY-1 cells treated with 50 ng/ml rIL30, were similar to that from HUVEC cultured with WPMY-1 CM obtained from WPMY-1 cells treated with 30 ng/ml rIL30. ANOVA: $p<0.001$. *$p<0.01$, Tukey HSD test *versus* CTRL. **$p<0.01$, Tukey HSD test *versus* WPMY-1 CM and CTRL. Results are expressed as mean ± SD and black dots overlaid on the bars represent individual replicates. Experiments were performed in triplicate.

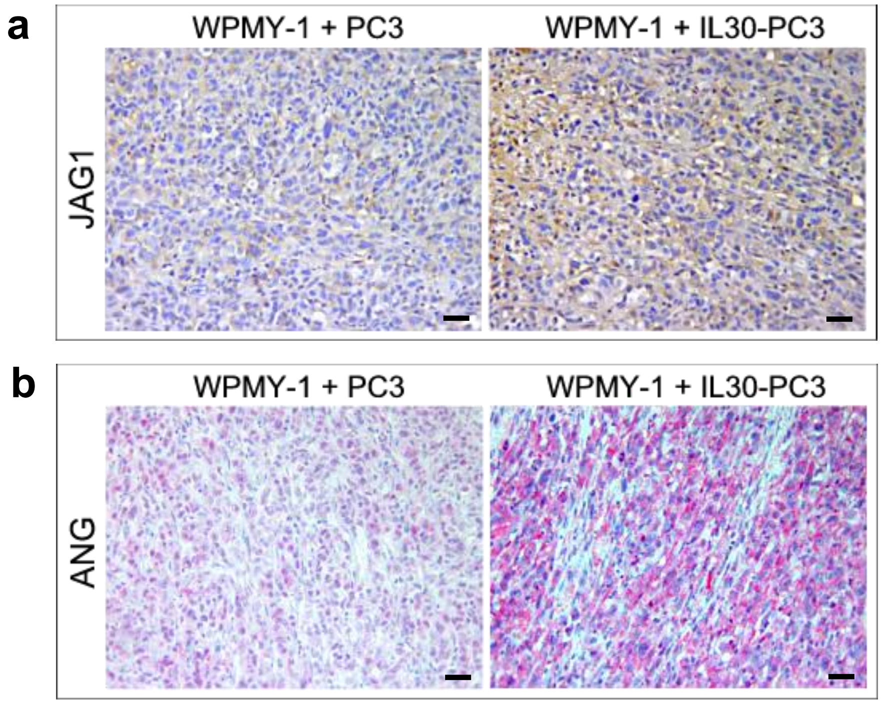

Fig. S8 Immunohistochemical staining of tumor xenografts revealed increased expression of JAG1 (**a**) and ANG (**b**), primarily in prostatic WPMY-1 fibroblasts, when co-injected subcutaneously with IL30-overexpressing PC3 cells (right panel), compared with WT (or EV) PC3 cells (left panel), in NSG mice. Results from WT-PC cells were comparable to those from EV-PC cells. Magnification: ×200. Scale bars: 40 µm.

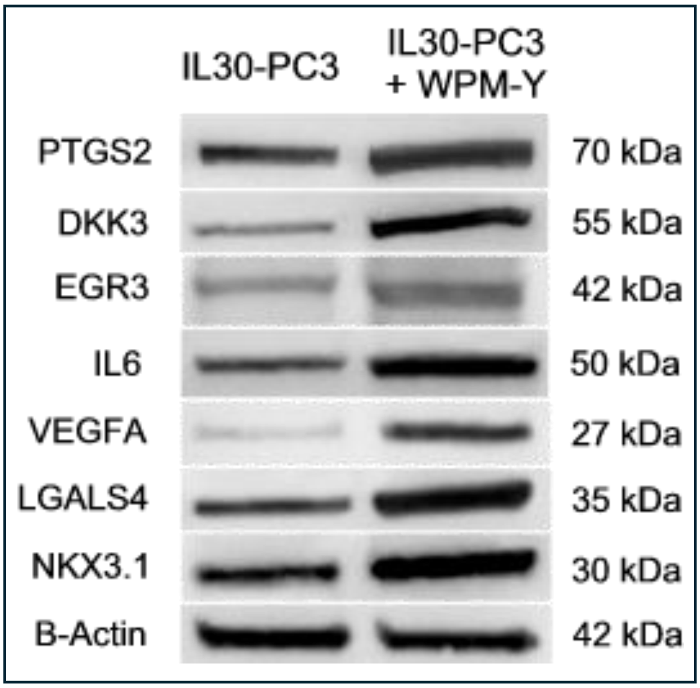

Fig. S9 Western blot analyses of PTGS2, DKK3, EGR3, IL6, VEGFA, LGALS4, NKX3.1 protein expression in IL30-PC3 cells cocultured or not with WPMY-1 cells.

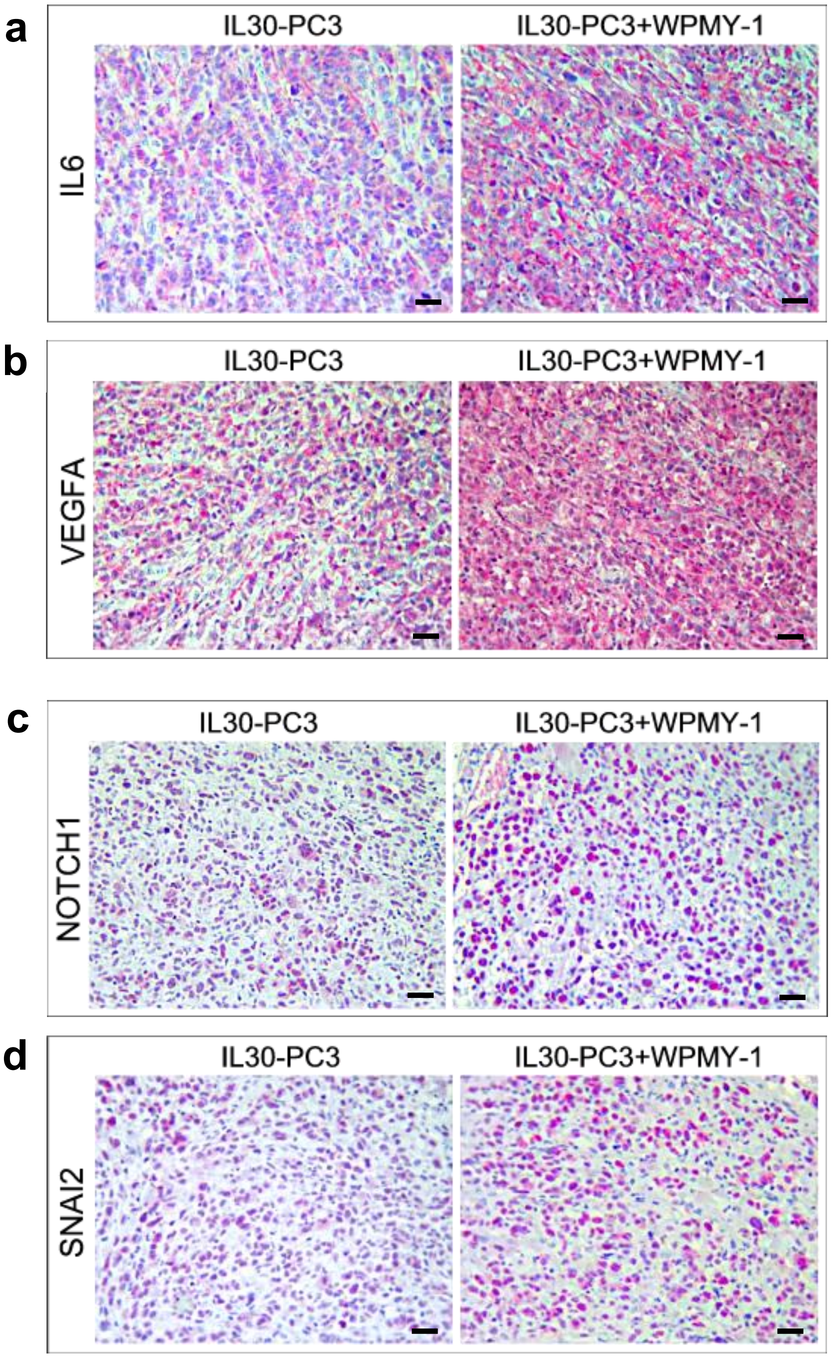

Fig. S10 Immunohistochemical staining reveals increased expression of IL6 (**a**), VEGFA (**b**), NOTCH1 (**c**), and SNAI2 (**d**) in tumor xenografts generated by subcutaneous injection of IL30-PC3 cells, either alone or in combination with prostatic WPMY-1 fibroblasts, in NSG mice. Comparable expression patterns were observed in xenografts derived from subcutaneous injection of IL30-DU145 cells, with or without WPMY-1 fibroblasts. Magnification: ×200. Scale bars: 20 µm.

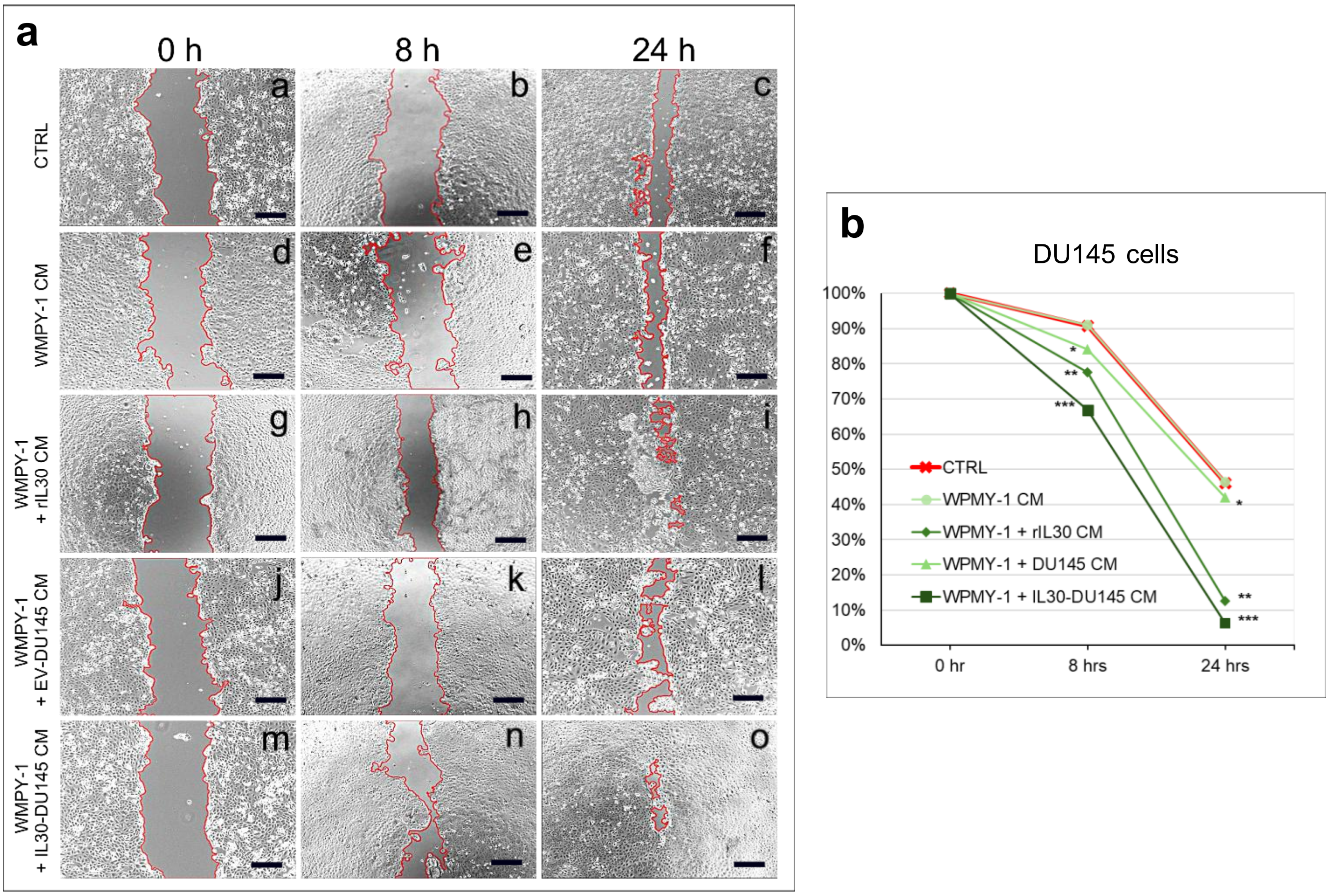

Fig. S11 Wound healing assay of PC cells treated with different conditioned medium. **a** Representative field images from an *in vitro* wound healing assay performed on DU145 cell monolayers either untreated (CTRL: a-c) or treated with conditioned media (CM) derived from: WPMY-1 cells (d-f); rIL30–treated WPMY-1 cells (g-i); EV-DU145+WPMY-1 co-cultures (j-l); or IL30-DU145+WPMY-1 co-cultures (m-o). Red lines indicate the wound margins used for quantification of cell migration. The scratch area at time 0 was normalized to 100%, and wound closure is expressed as the percentage of the remaining cell-free area. **b** Statistical analysis was performed using two-way ANOVA, revealing significant effects of treatment ($p < 0.0001$), time ($p < 0.0001$), and their interaction ($p<0.0001$). *$p<10^{-3}$, post hoc Welch's t-tests with Šidák correction *vs* CTRL and WPMY-1 CM. **$p<10^{-3}$, post hoc Welch's t-tests with Šidák correction *vs* CTRL, WPMY-1 CM, and WPMY-1 + DU145 CM. ***$p<10^{-3}$, post hoc Welch's t-tests with Šidák correction *vs* CTRL, WPMY-1 CM, WPMY-1 + rIL30 CM, and WPMY-1 + DU145 CM. Data are presented as mean ± SD of 3 biological replicates. Individual data points are included; however, in some cases both the data points and error bars may not be clearly visible due to the high consistency among replicates.

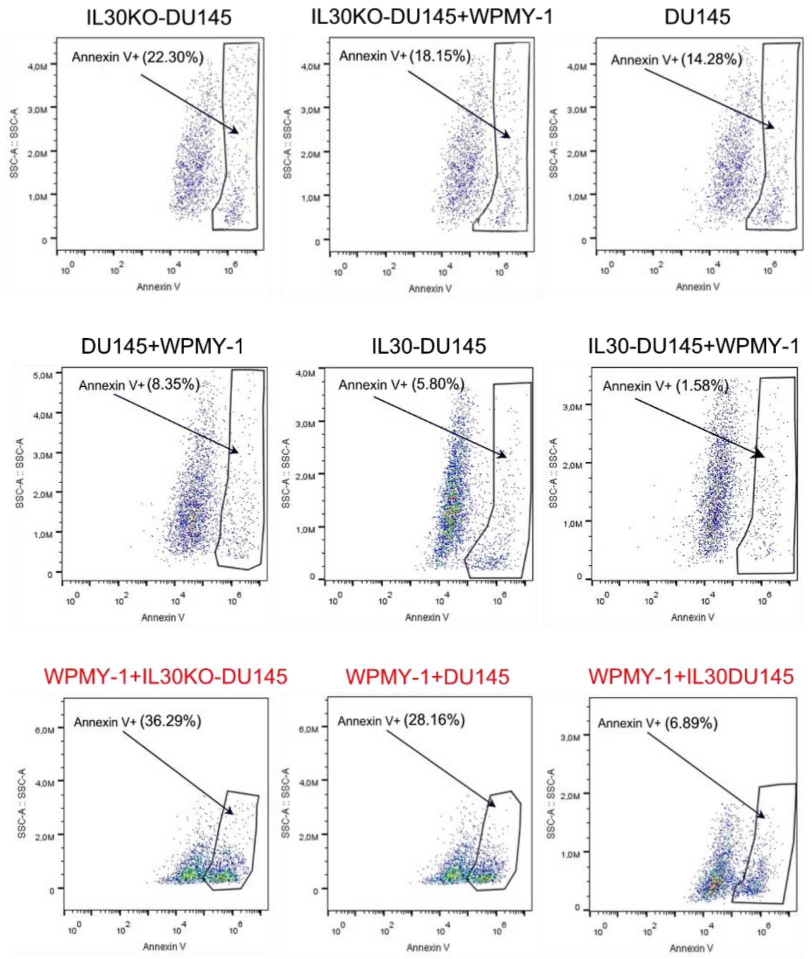

Fig. S12 Representative flow cytometry dot plots showing Annexin V$^+$ events in (WT- or EV-) DU145, IL30KO-DU145 and IL30-DU145 cells, cultured with or without WPMY-1 cells, and in WPMY-1 cells cultured with (WT- or EV-) DU145, IL30KO-DU145 or IL30-DU145 cells, isolated from spheroids developed in 2-OC device. Annexin V$^+$ cells (gated population, indicated by black arrows) correspond to early apoptotic cells, with raw percentages indicated in parentheses. Annexin V$^-$ cells (outside the gated region) represent viable, non-apoptotic cells. Results obtained by using WT-DU145 cells were comparable to those obtained with EV-DU145 cells. Experiments were performed in triplicate.

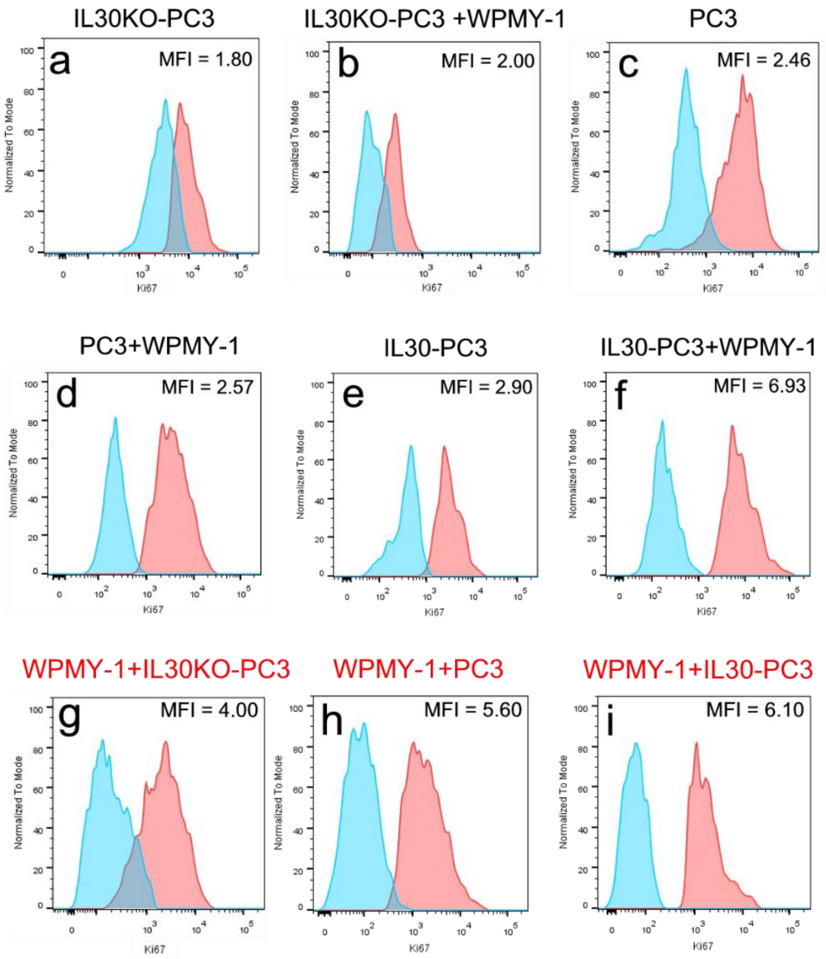

Fig. S13 Flow cytometry analysis of Ki67 fluorescence in EV-PC3, IL30KO-PC3 and IL30-PC3 cells, cultured with or without WPMY-1 cells (panels **a**–**f**), and in WPMY-1 cells cocultured with EV-PC3, IL30KO-PC3 or IL30-PC3 cells (panels **g**–**i**), in 2-OC. Red areas: specific Ab. Blue areas: isotype control. Results obtained by using WT-PC3 cells were comparable to those obtained with EV-PC3 cells. Experiments were performed in triplicate. MFI: mean fluorescence intensity.

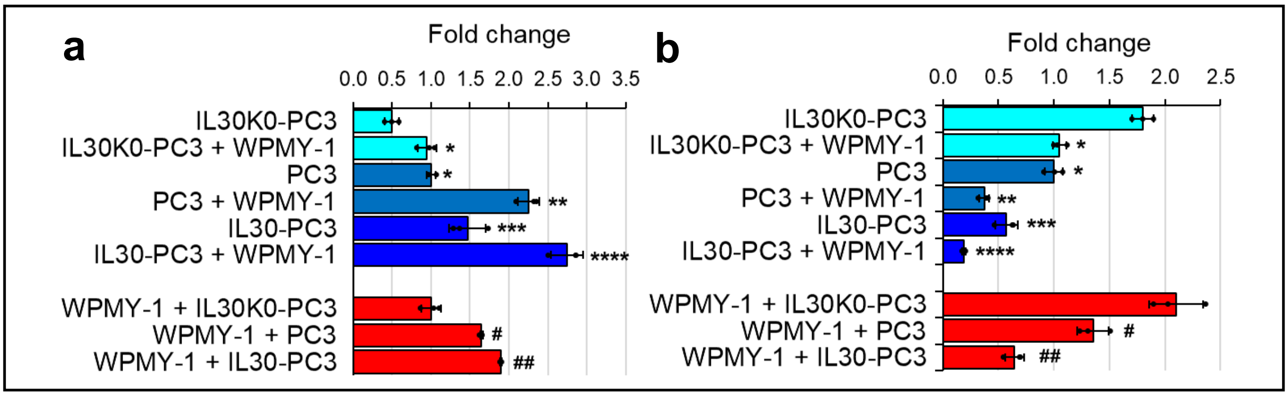

Fig. S14

a. Proliferation of PC3, IL30KO-PC3 and IL30-PC3 cells cultured with or without WPMY-1 cells (blue bars), and proliferation of WPMY-1 cells cultured with PC3, IL30KO-PC3 or IL30-PC3 cells (red bars), in the 2-OC device, as assessed by flow cytometry with Ki67$^+$ staining. ANOVA: p<0.001. *p<0.05, Tukey HSD test *versus* IL30KO-PC3 cells. **p<0.05, Tukey HSD test *versus* IL30KO-PC3 cells, cultured with or without WPMY-1 cells, and PC3 cells. ***p<0.05, Tukey HSD test *versus* IL30KO-PC3 cells, cultured with or without WPMY-1 cells, and PC3 cells, cultured with or without WPMY-1 cells. ****p<0.05, Tukey HSD test *versus* IL30KO-PC3 cells, cultured with or without WPMY-1 cells, PC3 cells, cultured with or without WPMY-1 cells, and IL30-PC3 cells. $^{\#}p$<0.01, Tukey HSD test *versus* WPMY-1 + IL30KO-PC3 cells. $^{\#\#}p$<0.01, Tukey HSD test *versus* WPMY-1 cells cultured with IL30KO-PC3 or PC3 cells. Results obtained by using WT-PC3 cells were comparable to those obtained with EV-PC3 cells. Experiments were performed in triplicate. Cell proliferation (Ki67$^+$cells/μl) is expressed as fold change relative to PC3 cells and reported as mean ± SD. Black dots overlaid on the bars represent individual replicates. Experiments were performed in triplicate.

b. Apoptosis of PC3, IL30KO-PC3 and IL30-PC3 cells cultured with or without WPMY-1 cells (blue bars), and apoptosis of WPMY-1 cells cultured with PC3, IL30KO-PC3 or IL30-PC3 cells (red bars), in the 2-OC device, as assessed by flow cytometry with Annexin V staining. ANOVA: p<0.001. *p<0.05, Tukey HSD test *versus* IL30KO-PC3 cells. **p<0.05, Tukey HSD test *versus* IL30KO-PC3 cells, cultured with or without WPMY-1 cells, and PC3 cells.

***$p<0.05$, Tukey HSD test *versus* IL30KO-PC3 cells, cultured with or without WPMY-1 cells, and PC3 cells, cultured with or without WPMY-1 cells. ****$p<0.05$, Tukey HSD test *versus* IL30KO-PC3 cells, cultured with or without WPMY-1 cells, PC3 cells, cultured with or without WPMY-1 cells, and IL30-PC3 cells. #$p<0.05$, Tukey HSD test *versus* WPMY-1 + IL30KO-PC3 cells. ##$p<0.01$, Tukey HSD test *versus* WPMY-1 cells cultured with IL30KO-PC3 or PC3 cells. Results obtained by using WT-PC3 cells were comparable to those obtained with EV-PC3 cells. Experiments were performed in triplicate. Frequency of apoptotic events (Annexin V^+cells/μl) is expressed as fold change relative to PC3 cells and reported as mean ± SD. Black dots overlaid on the bars represent individual replicates. Experiments were performed in triplicate.

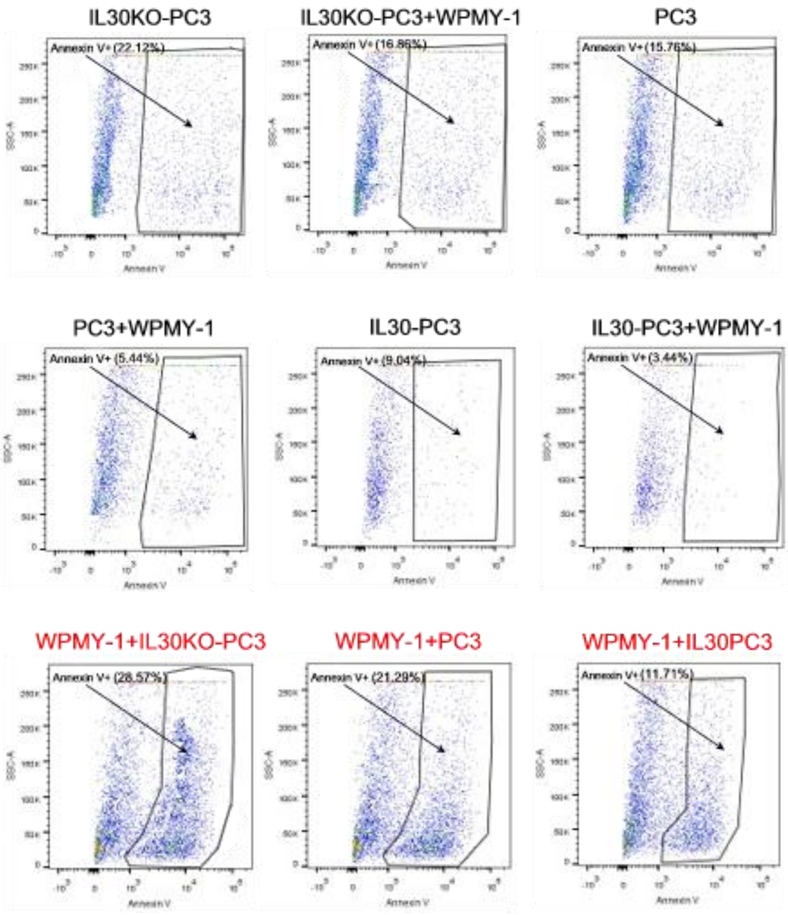

Fig. S15 Representative flow cytometry dot plots showing Annexin V^+ events in (WT- or EV-) PC3, IL30KO- and IL30-PC3 cells, cultured with or without WPMY-1 cells, and in WPMY-1 cells cocultured with (WT- or EV-) PC3, IL30KO- or IL30-PC3 cells, isolated from spheroids cultured in 2-OC device. Annexin V^+ cells (gated population, indicated by black arrows) correspond to early apoptotic cells, with raw percentages indicated in parentheses. Annexin V^- cells (outside the gated region) represent viable, non-apoptotic cells. Results obtained by using WT-PC3 cells were comparable to those obtained with EV-PC3 cells.

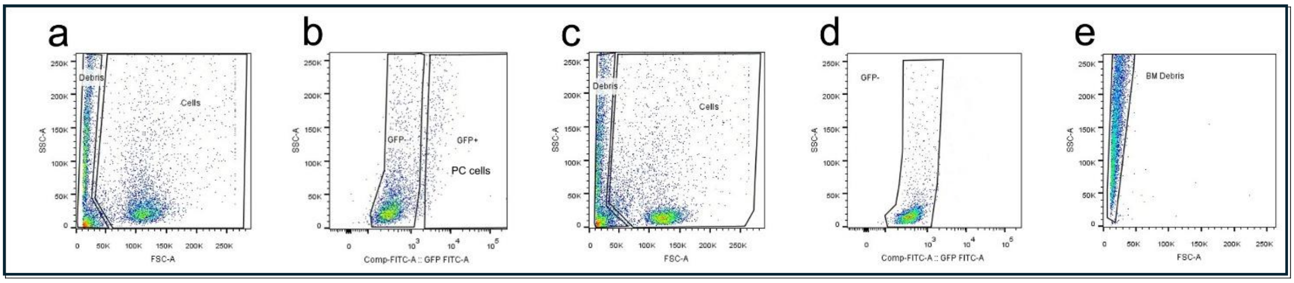

Fig. S16 Gating strategy to quantify the migration of GFP^+ PC cells from the tumor compartment to the BM compartment in 2-OC.To identify cellular events and exclude debris and non-cellular signals, samples were first gated on side scatter area (SSC-A) and forward scatter area (FSC-A) (**a**). Within the gated cell population (“Cells”), GFP^+ PC cells were identified using SSC-A and the FITC channel (**b**). Gating thresholds were established using BM scaffolds seeded only with hMSCs and $CD34^+$ cells (**c**), to define the GFP^- population (**d**). As negative controls, BM scaffolds with no seeded cells were analyzed (**e**) to exclude potential background debris.

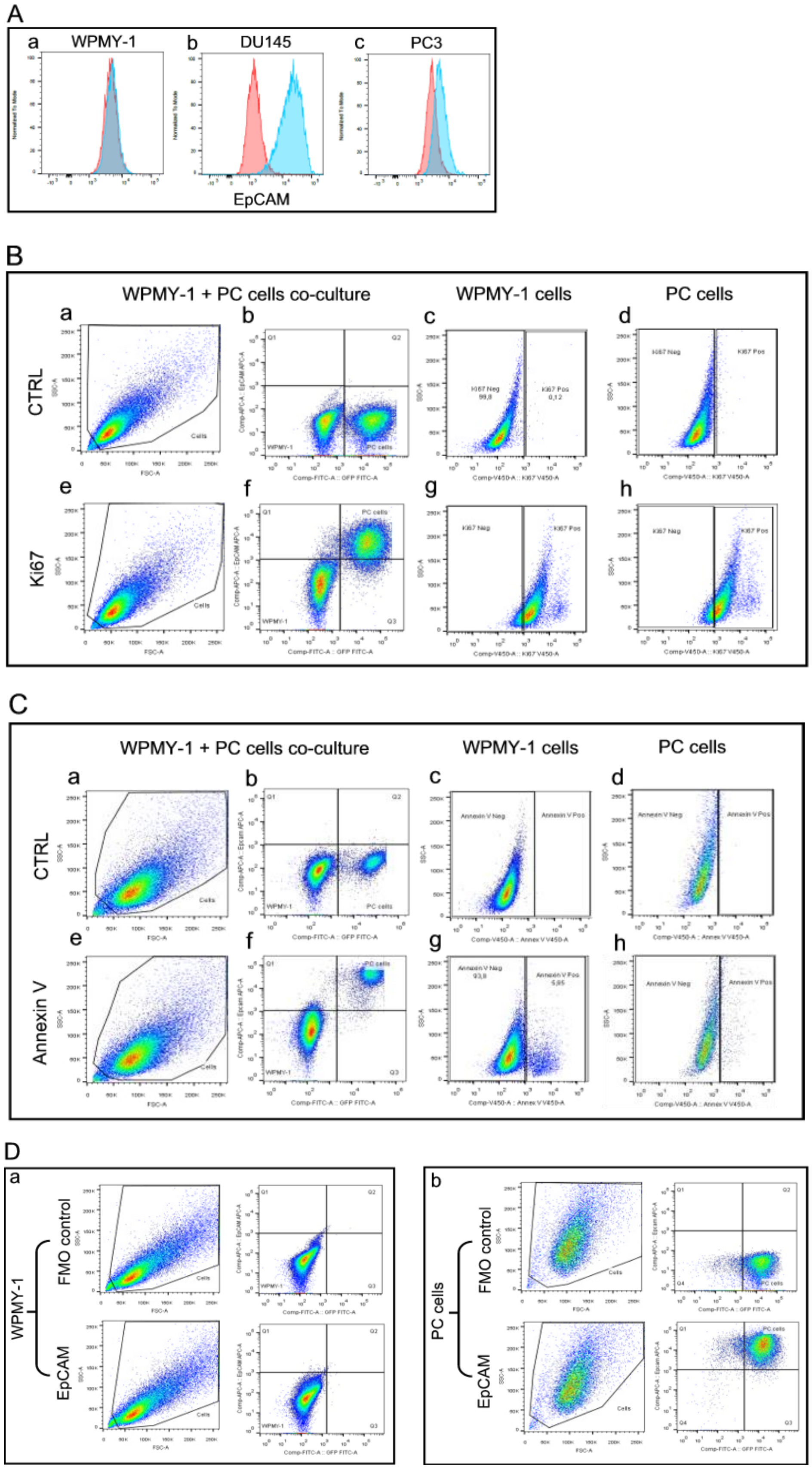

Fig. S17 Gating strategy used for Ki67 and Annexin V staining.

A. Flow cytometry analysis of EpCAM expression in WPMY-1 cells (**a**), DU145 cells (**b**) and PC3 cells (**c**).

B, C. Cells were first gated based on forward scatter (FSC) and side scatter (SSC) parameters, to exclude debris and non-cellular events (**a** and **e**). Within the gated “Cells” population, WPMY-1 and PC cell populations (DU145 and PC3 cells) were identified based on their expression of EpCAM (APC channel) and GFP (FITC channel). WPMY-1 cells were negative for both EpCAM and GFP, whereas PC cells were EpCAM- and GFP-positive (**b** and **f**). Subsequently, WPMY-1 and PC cell populations were analyzed separately, and gating thresholds for Ki67 (BV421) and Annexin V (Pacific Blue) positivity were established using unstained control samples (**c** and **d**). These thresholds were then applied to WPMY-1 and PC cells in Ki67- or Annexin V-stained samples (**g** and **h**). CTRL: samples not stained for Ki67, Annexin V or EpCAM.

D. EpCAM stained and fluorescence minus one (FMO) controls were used to discriminate between WPMY-1 (**a**) and PC cells (**b**) and to set the gating thresholds.

Heatmaps of gene expression analyses corresponding to Figures 3–5

(color scales were adjusted to the dynamic range of each dataset to allow optimal visualization of fold changes)

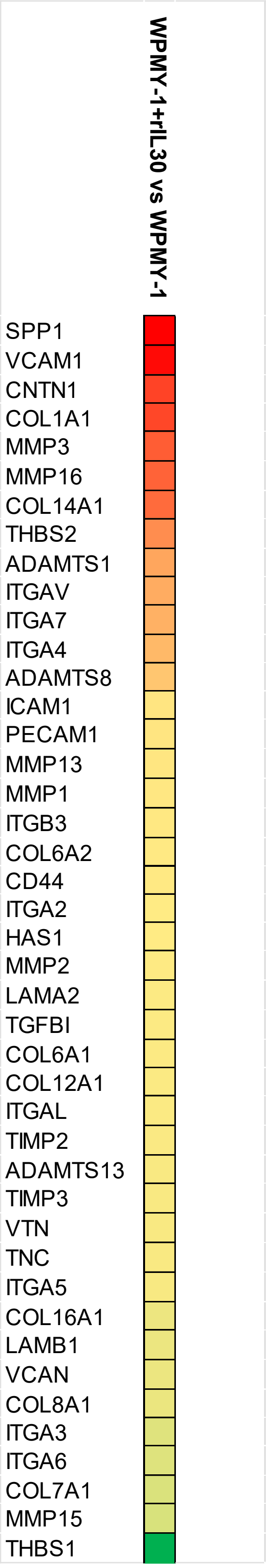

Fig. 3a Fold changes in mRNA expression of ECM-related genes in WPMY-1 cells treated with rIL30 (50 ng/ml) compared with untreated cells. A twofold change in gene expression was considered significant (*p*<0.001). Only genes with a fold change > 2 are shown. Experiments were performed in triplicate.

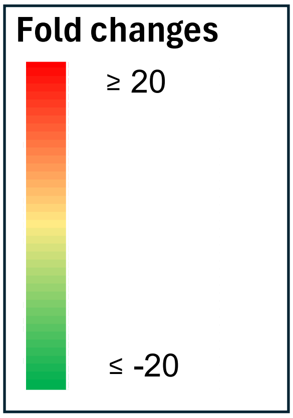

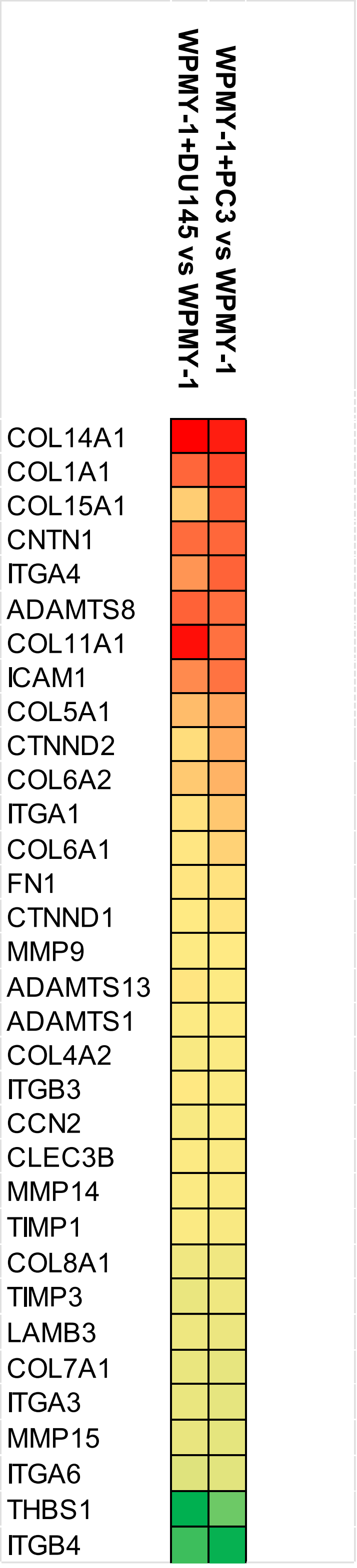

Fig. 3c Fold changes in mRNA expression of ECM-related genes in WPMY-1 cells co-cultured with WT-DU145 or WT-PC3 cells, compared with WPMY-1 cells cultured alone. A twofold change in gene expression was considered significant ($p<0.001$). Only genes with a fold change > 2 in both co-culture conditions are shown. Experiments were performed in triplicate.

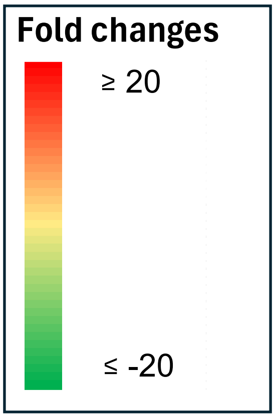

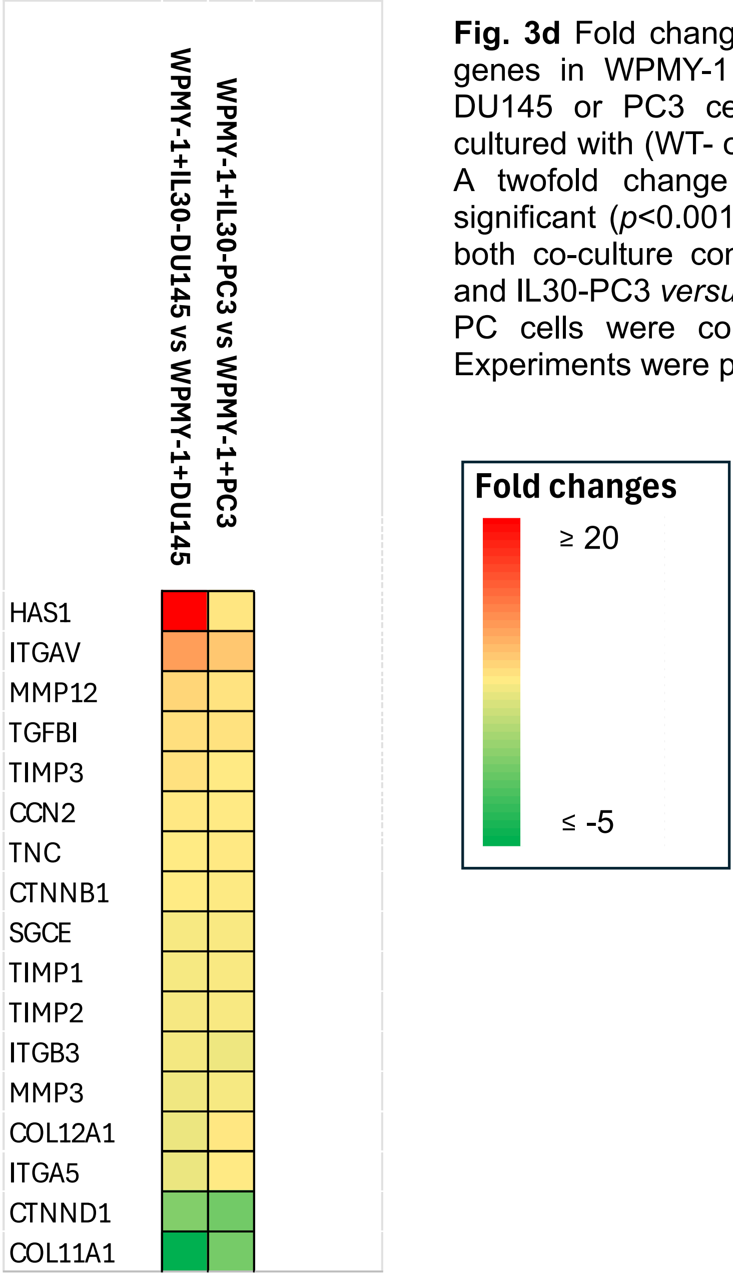

Fig. 3d Fold changes in mRNA expression of ECM-related genes in WPMY-1 cells co-cultured with IL30-expressing DU145 or PC3 cells, compared with WPMY-1 cells co-cultured with (WT- or EV-) DU145 or (WT- or EV-) PC3 cells. A twofold change in gene expression was considered significant (p<0.001). Only genes with a fold change > 2 in both co-culture conditions (IL30-DU145 *versus* EV-DU145 and IL30-PC3 *versus* EV-PC3) are shown. Results from WT-PC cells were comparable to those from EV-PC cells. Experiments were performed in triplicate.

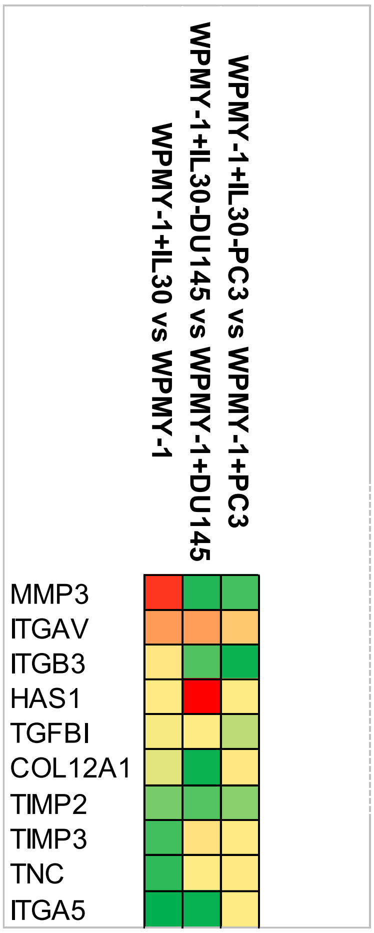

Fig. 3g Fold changes in mRNA expression of ECM-related genes under the following conditions: WPMY-1 cells untreated or treated with rIL30; WPMY-1 cells co-cultured with WT, EV, or IL30-overexpressing DU145 or PC3 cells. Only genes regulated in all three conditions are shown. A twofold change in gene expression was considered significant ($p<0.001$). Results from WT-PC cells were comparable to those from EV-PC cells. Experiments were performed in triplicate.

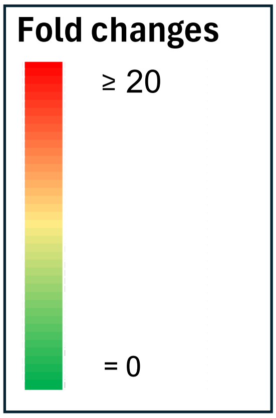

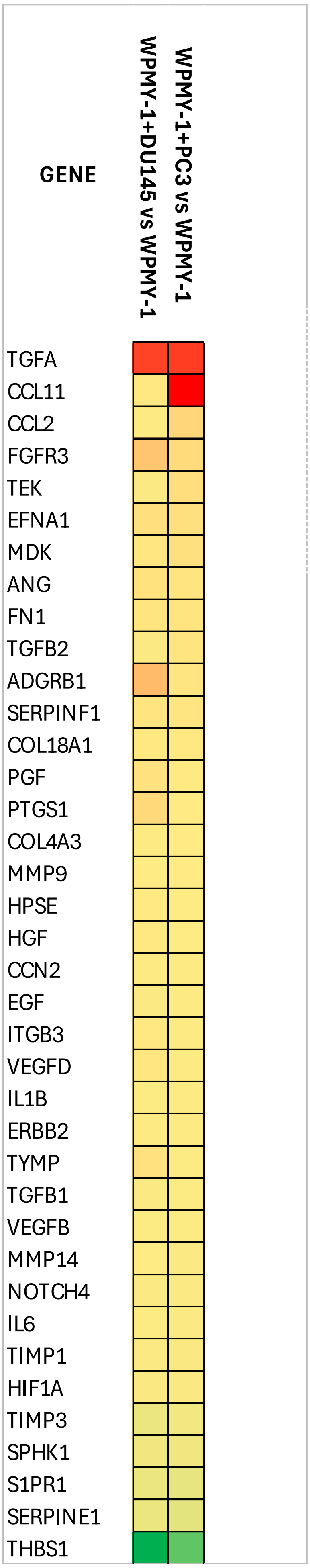

Fig. 4a Fold changes in mRNA expression of angiogenesis-related genes in WPMY-1 cells co-cultured with WT-DU145 or WT-PC3 cells, compared with WPMY-1 cells cultured alone. A twofold change in gene expression was considered significant ($p<0.001$). Only genes with a fold change > 2 in both co-culture conditions are shown. Experiments were performed in triplicate.

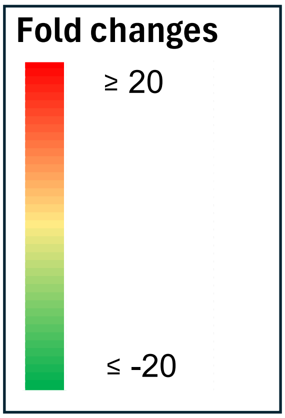

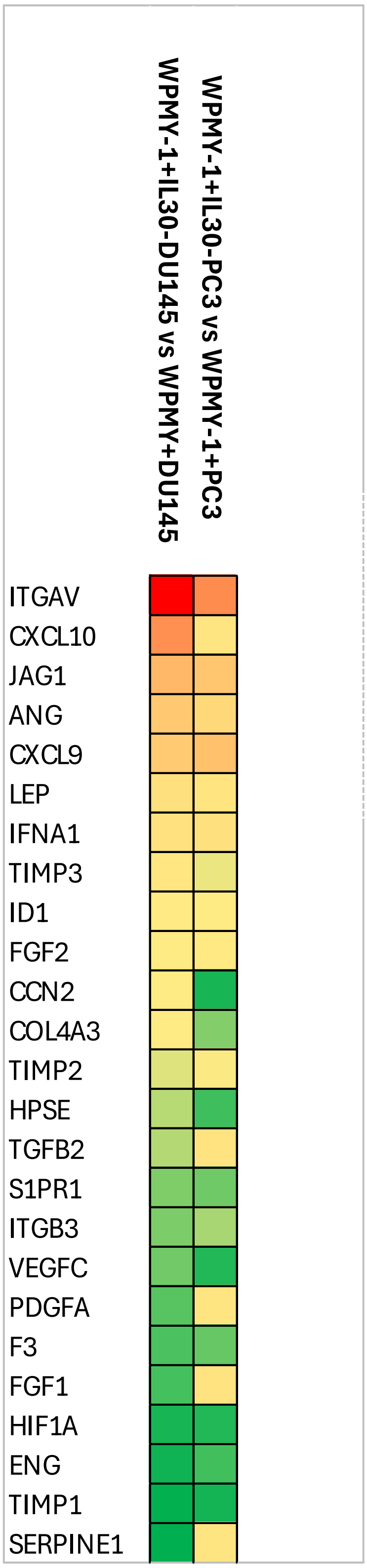

Fig. 4c Fold changes in mRNA expression of angiogenesis-related genes in WPMY-1 cells co-cultured with IL30-expressing DU145 or PC3 cells, compared with WPMY-1 cells co-cultured with (WT- or EV-) DU145 or (WT- or EV-) PC3 cells. A twofold change in gene expression was considered significant (*p*<0.001). Only genes with a fold change > 2 in both co-culture conditions (IL30-DU145 *versus* EV-DU145 and IL30-PC3 *versus* EV-PC3) are shown. Results from co-culture with WT PC cells were similar to those from co-culture with EV PC cells. Experiments were performed in triplicate.

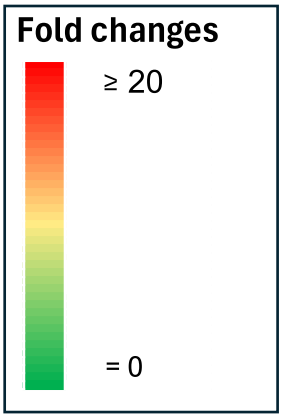

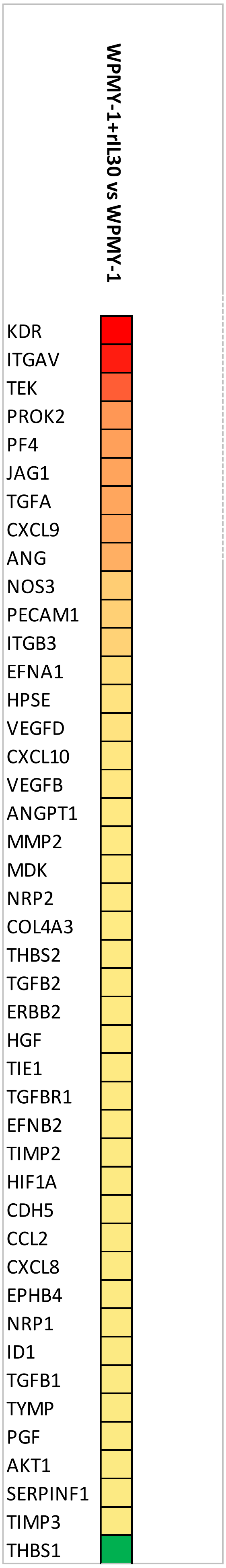

Fig. 4e Fold changes in mRNA expression levels of angiogenesis-related genes in rIL30 treated versus untreated WPMY-1 cells. A two-fold change in gene expression was considered significant at $p<0.001$. Only genes with a fold change > 2 are shown. Experiments were performed in triplicate.

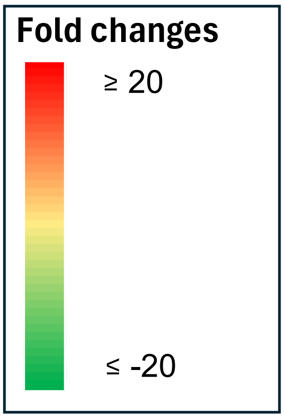

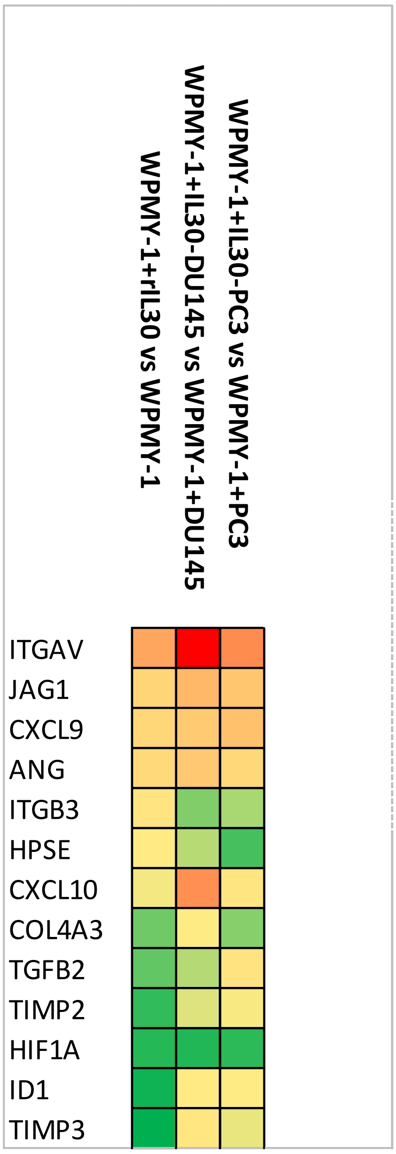

Fig. 4f Fold changes in mRNA expression of angiogenesis-related genes under the following conditions: WPMY-1 cells untreated or treated with rIL30; WPMY-1 cells co-cultured with WT, EV, or IL30-overexpressing DU145 or PC3 cells. Only genes regulated in all three conditions are shown. A twofold change in gene expression was considered significant ($p<0.001$). Results from WT-PC cells were comparable to those from EV-PC cells. Experiments were performed in triplicate.

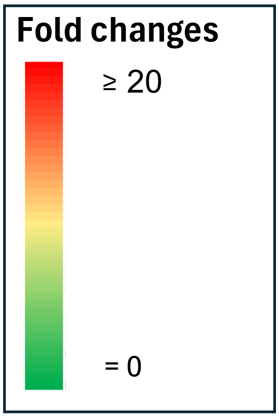

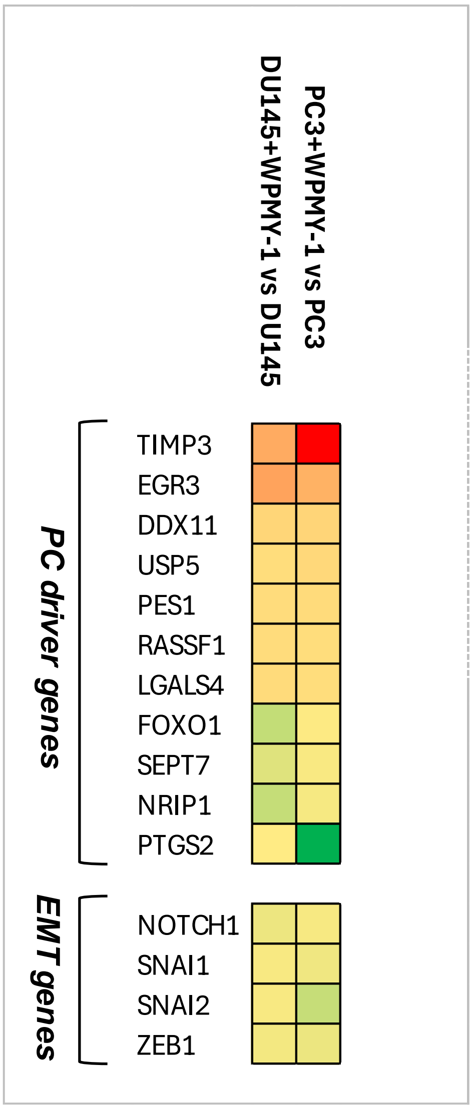

Fig. 5a Fold changes in mRNA expression of PC driver and EMT genes in WT-DU145 or WT-PC3 cells cultured alone or co-cultured with WPMY-1 cells. A twofold change in gene expression was considered significant ($p<0.001$). Only genes with a fold change > 2 in both co-culture conditions are shown. Experiments were performed in triplicate.

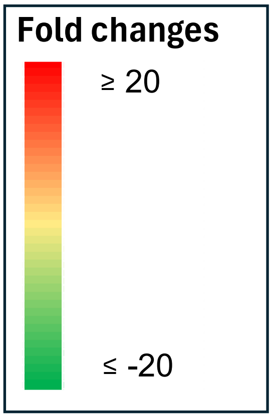

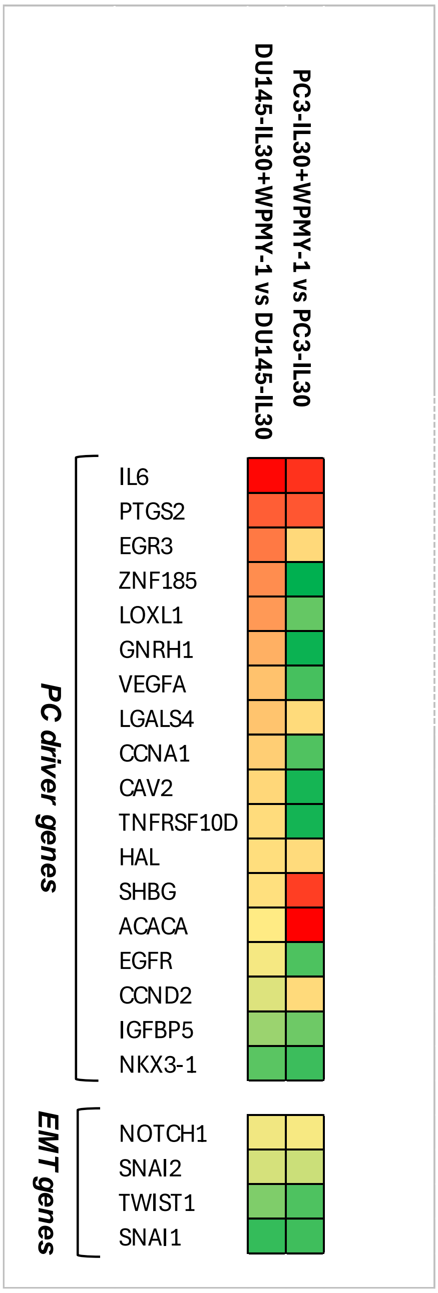

Fig. 5c Fold changes in mRNA expression of PC driver and EMT genes in IL30-overexpressing DU145 or PC3 cells cultured alone or co-cultured with WPMY-1 cells. A twofold change in gene expression was considered significant ($p<0.001$). Only genes with a fold change > 2 in both co-culture conditions (IL30-DU145 and IL30-PC3) are shown. Experiments were performed in triplicate.

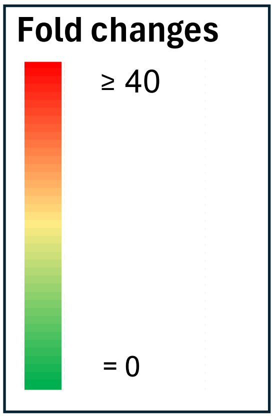

Original uncropped WB membranes in alphabetical order

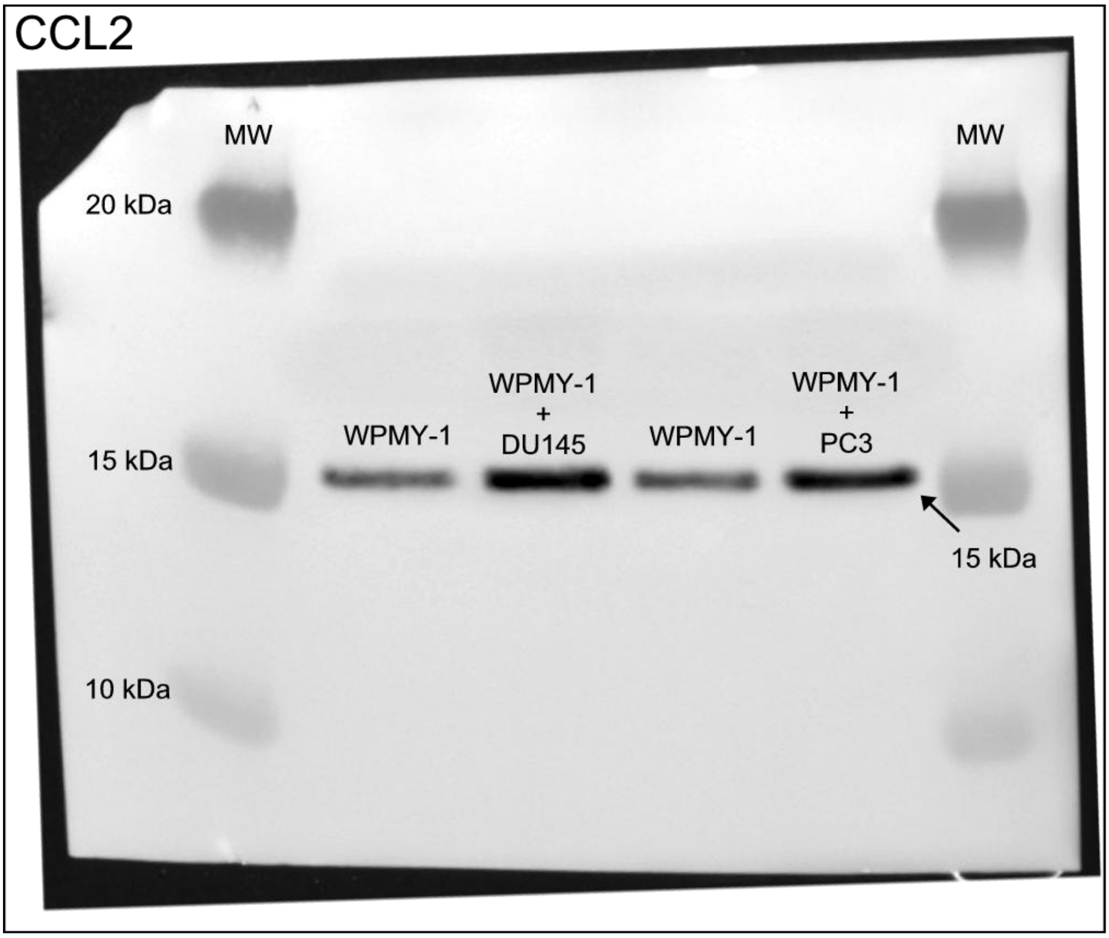

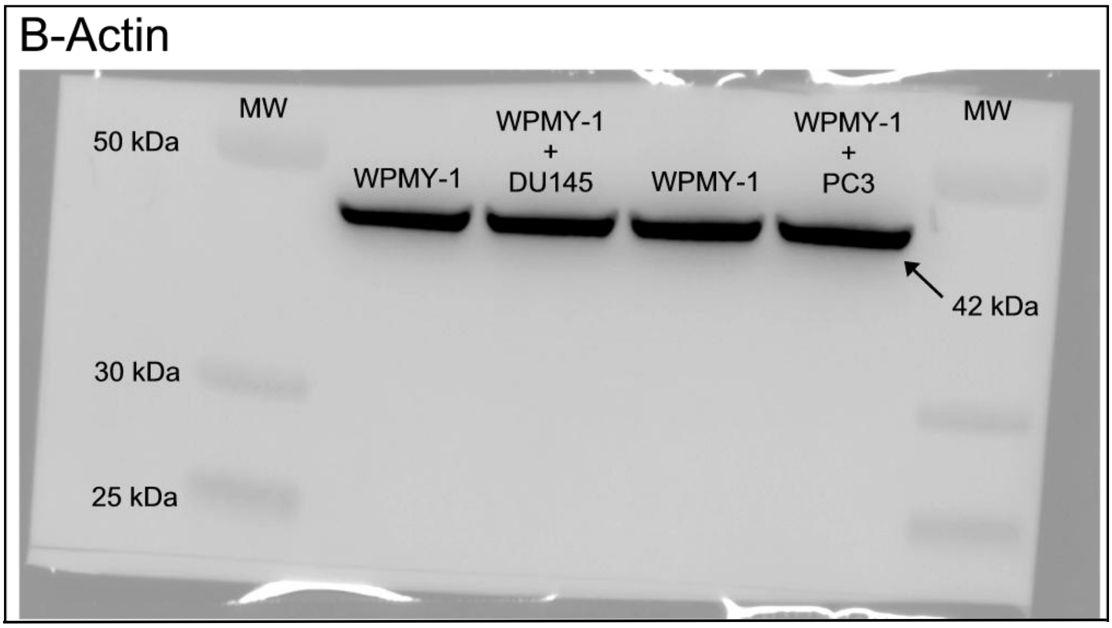

MW: molecular weight marker.

CCL11

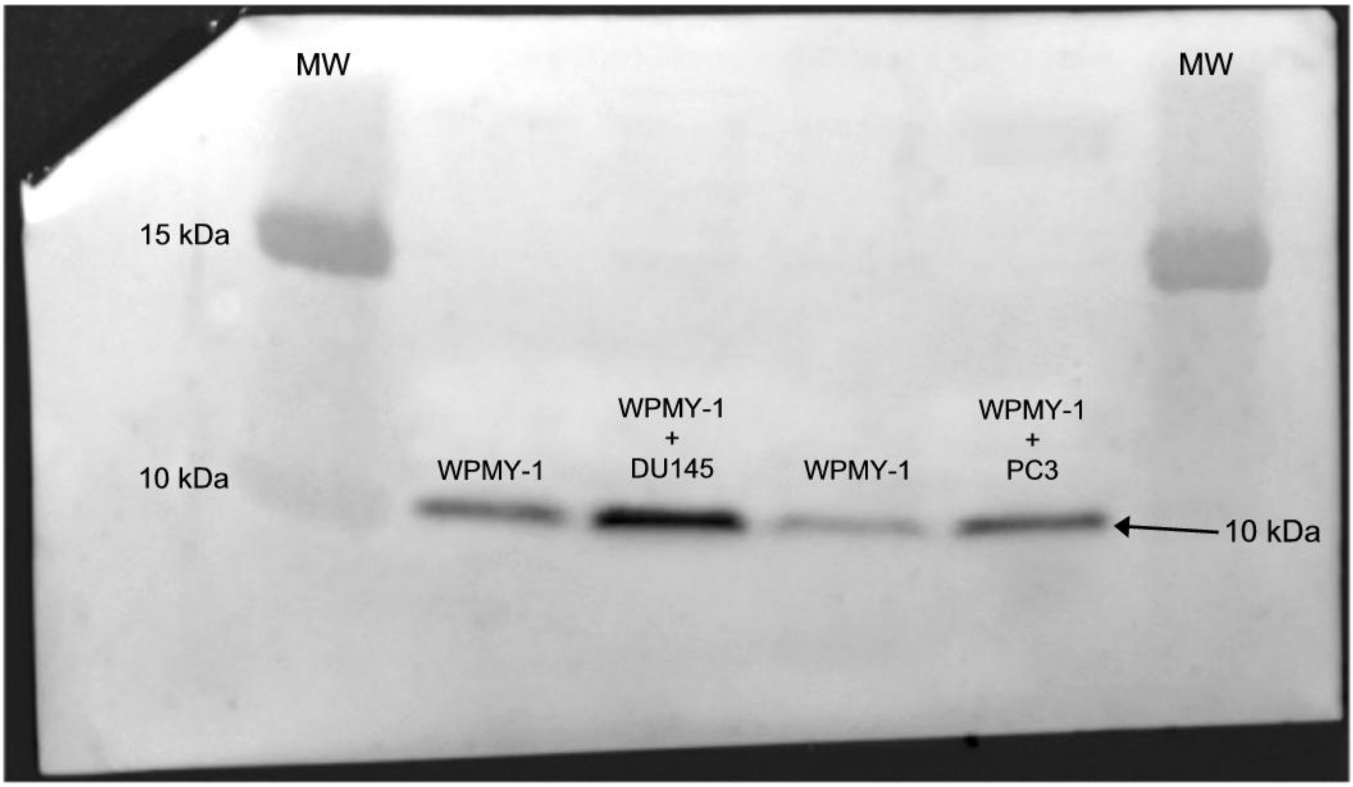

B-Actin

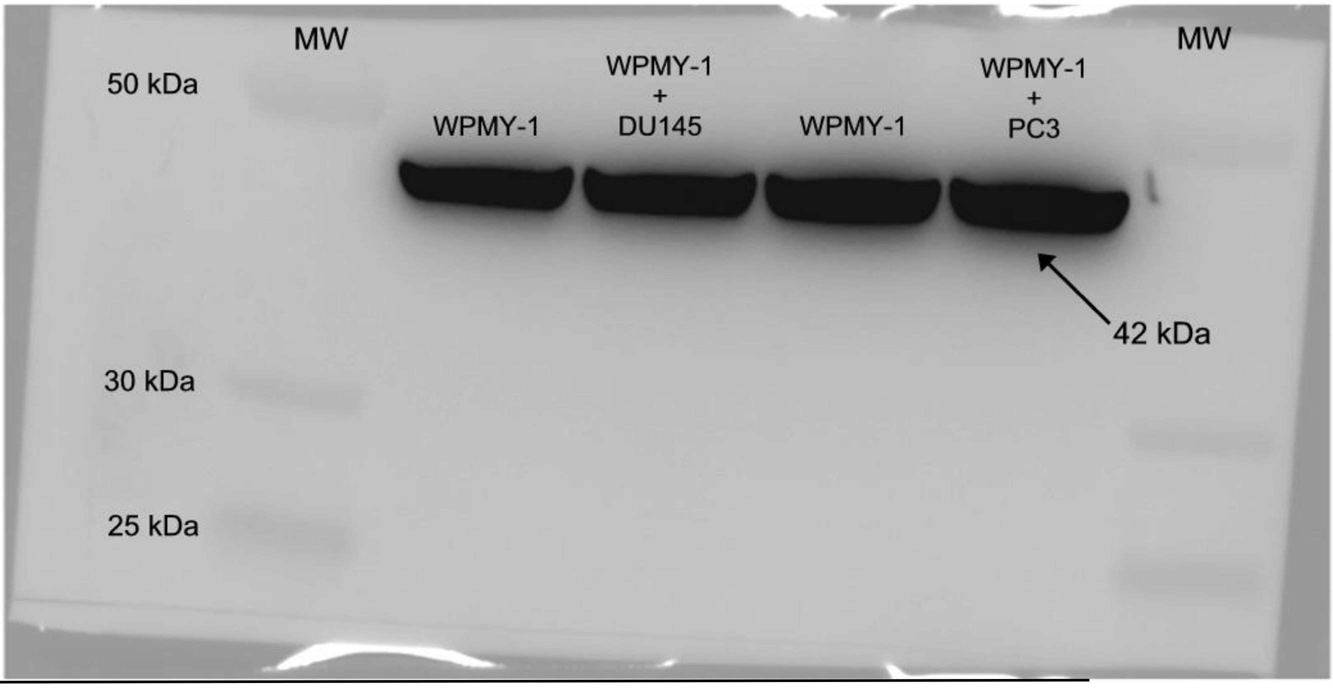

MW: molecular weight marker.

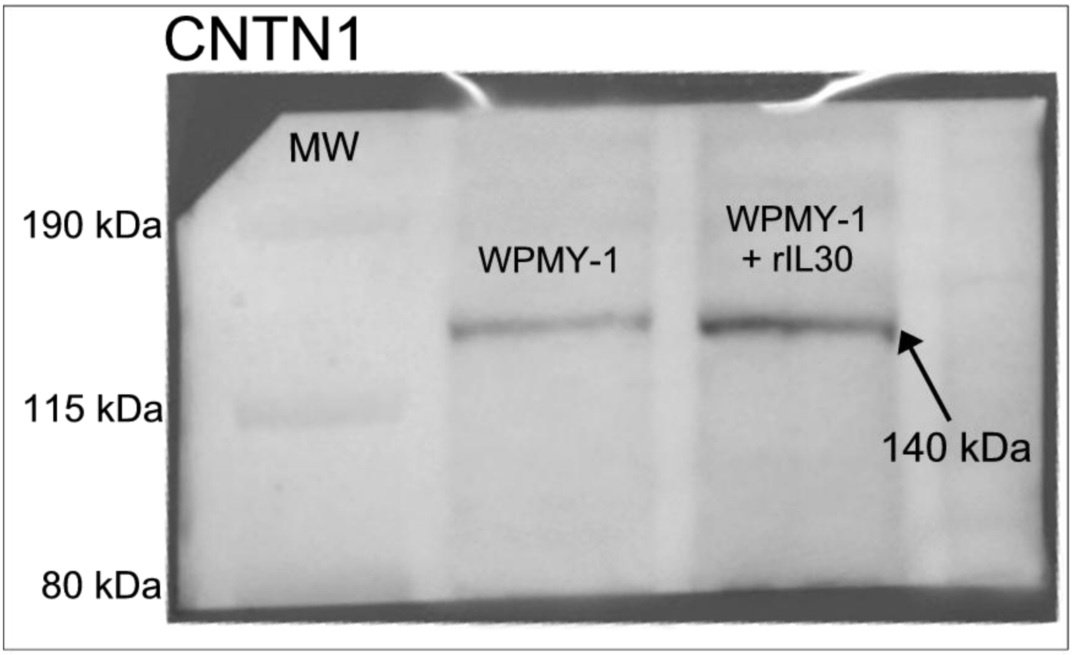

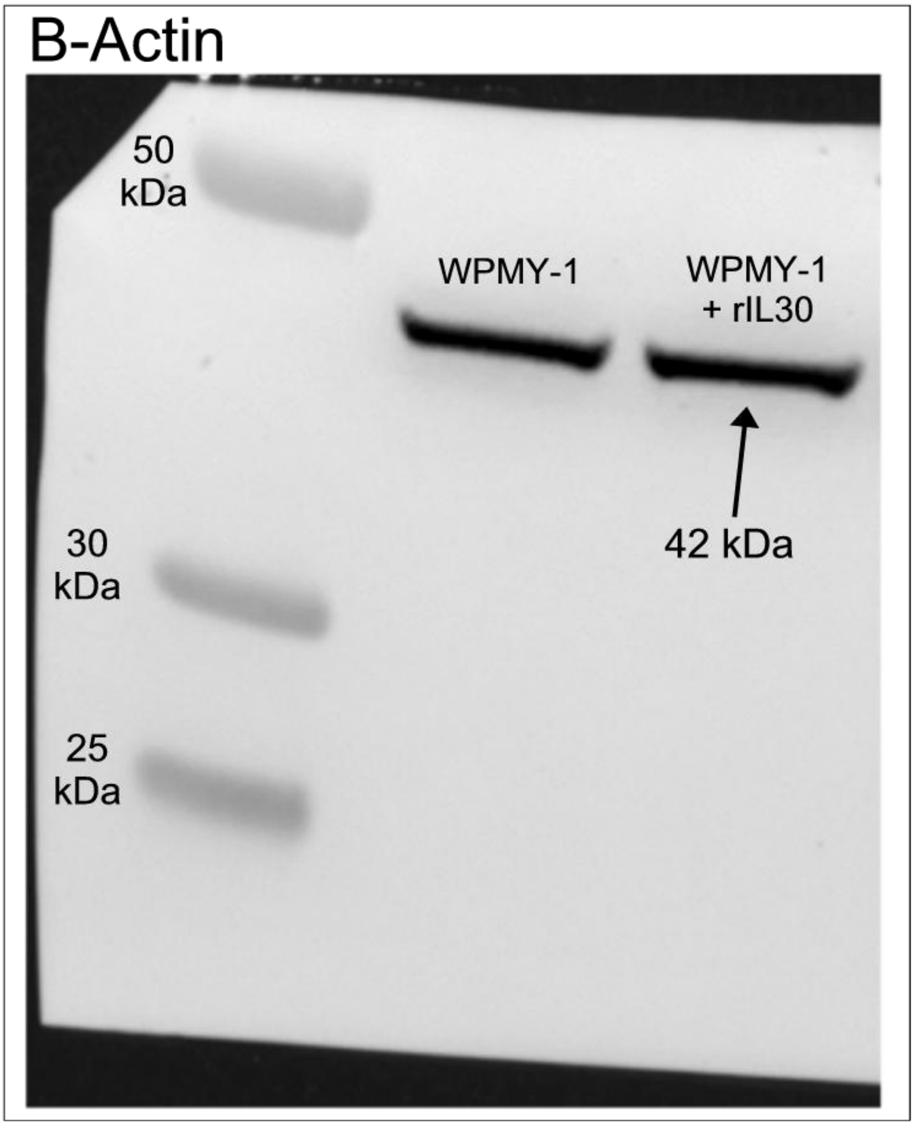

MW: molecular weight marker.

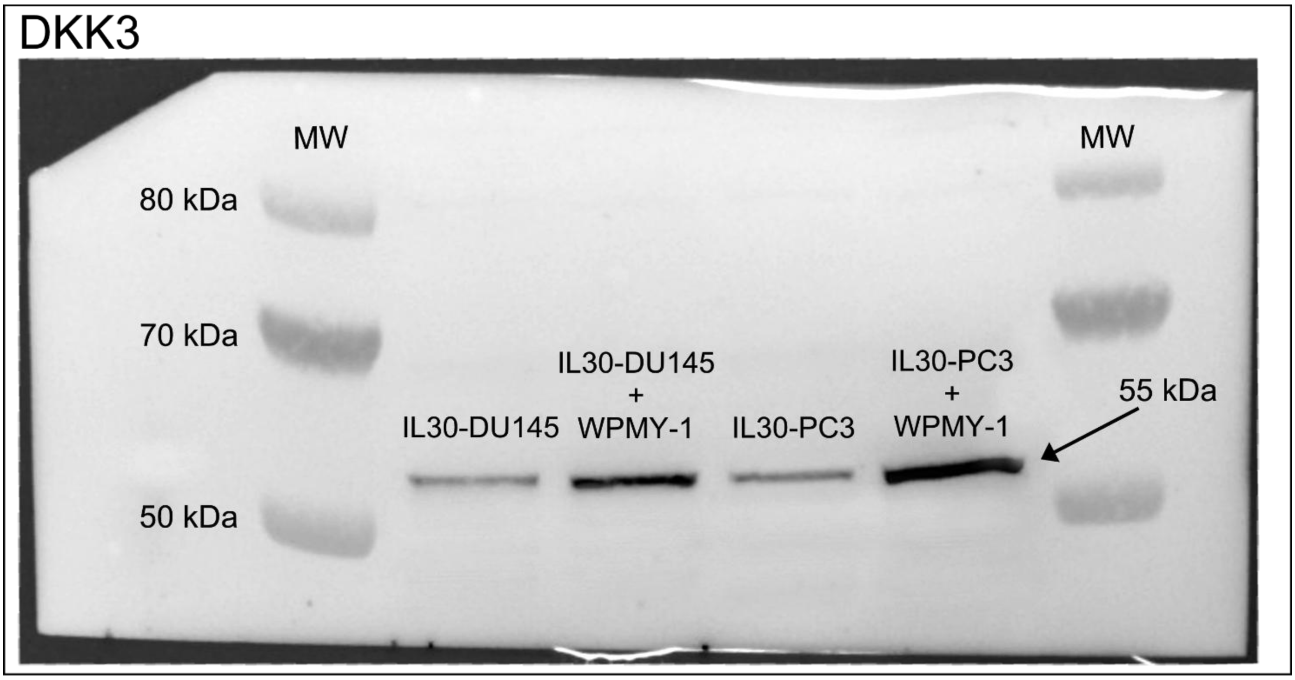

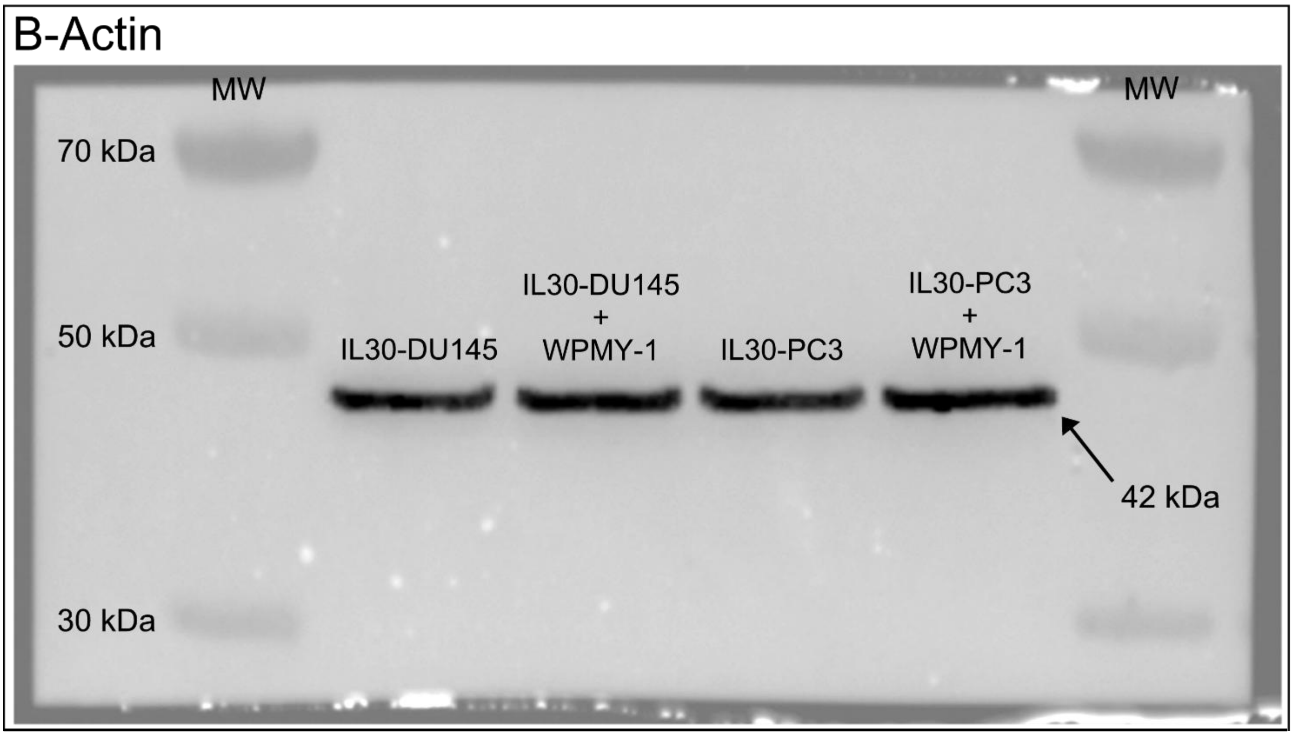

MW: molecular weight marker.

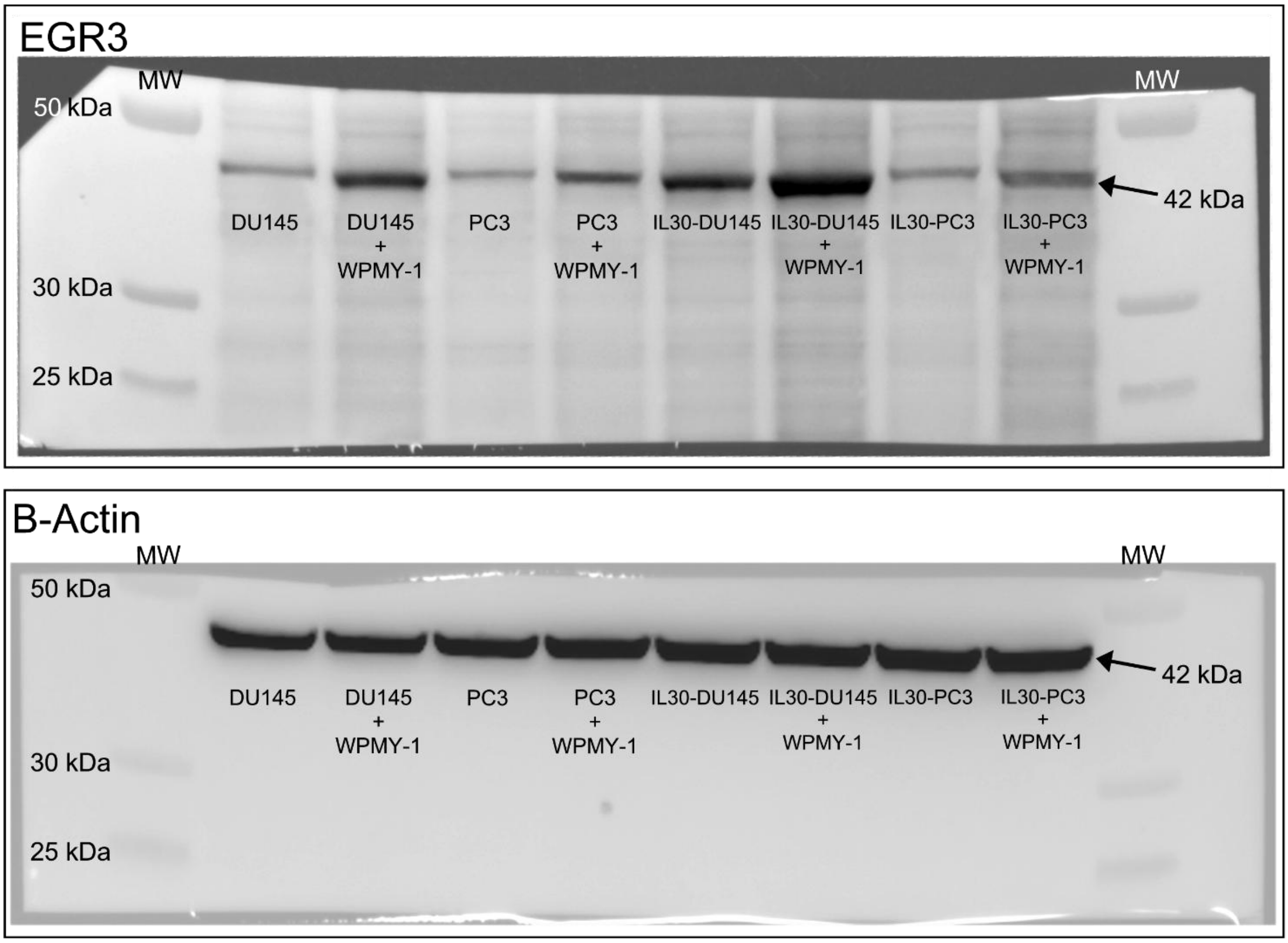

MW: molecular weight marker.

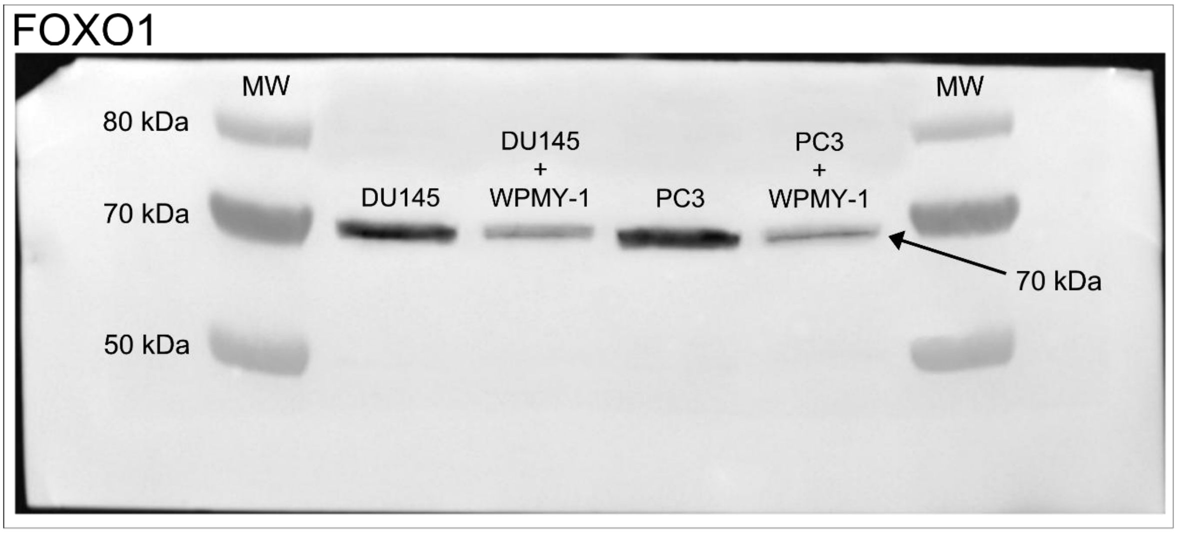

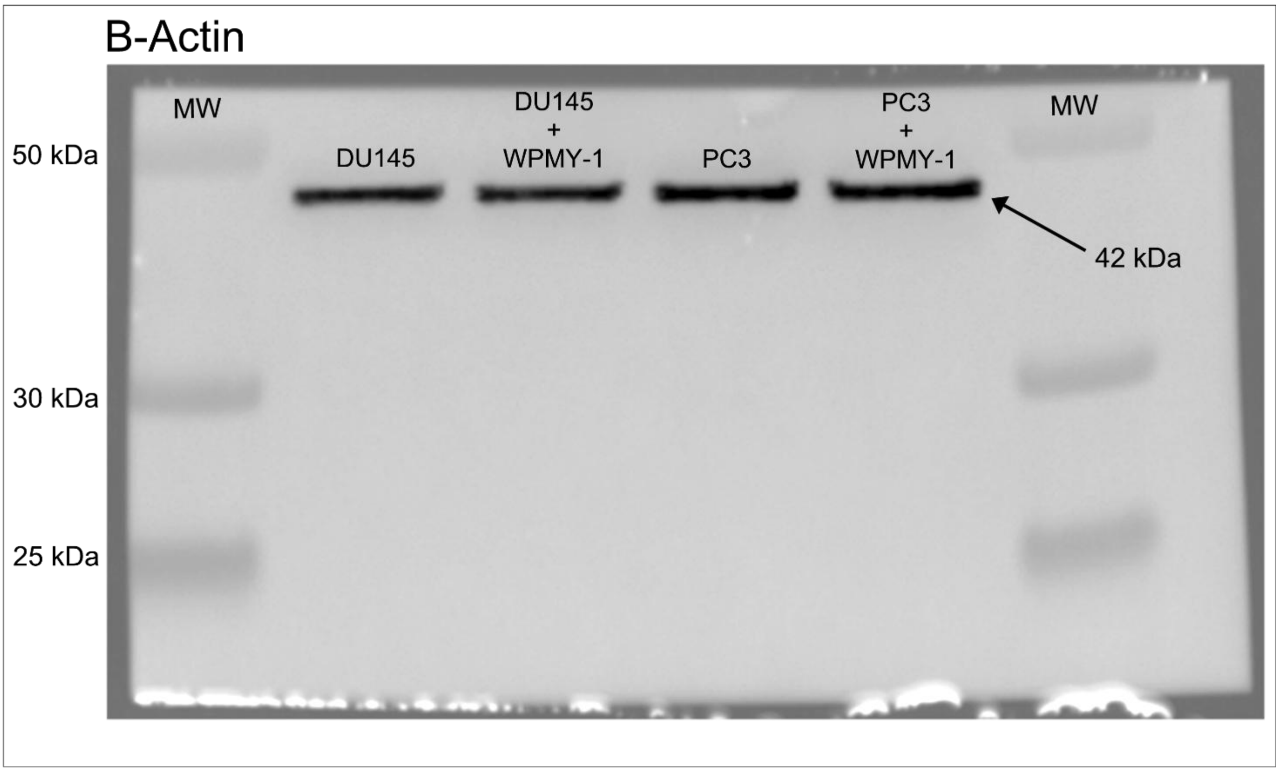

MW: molecular weight marker.

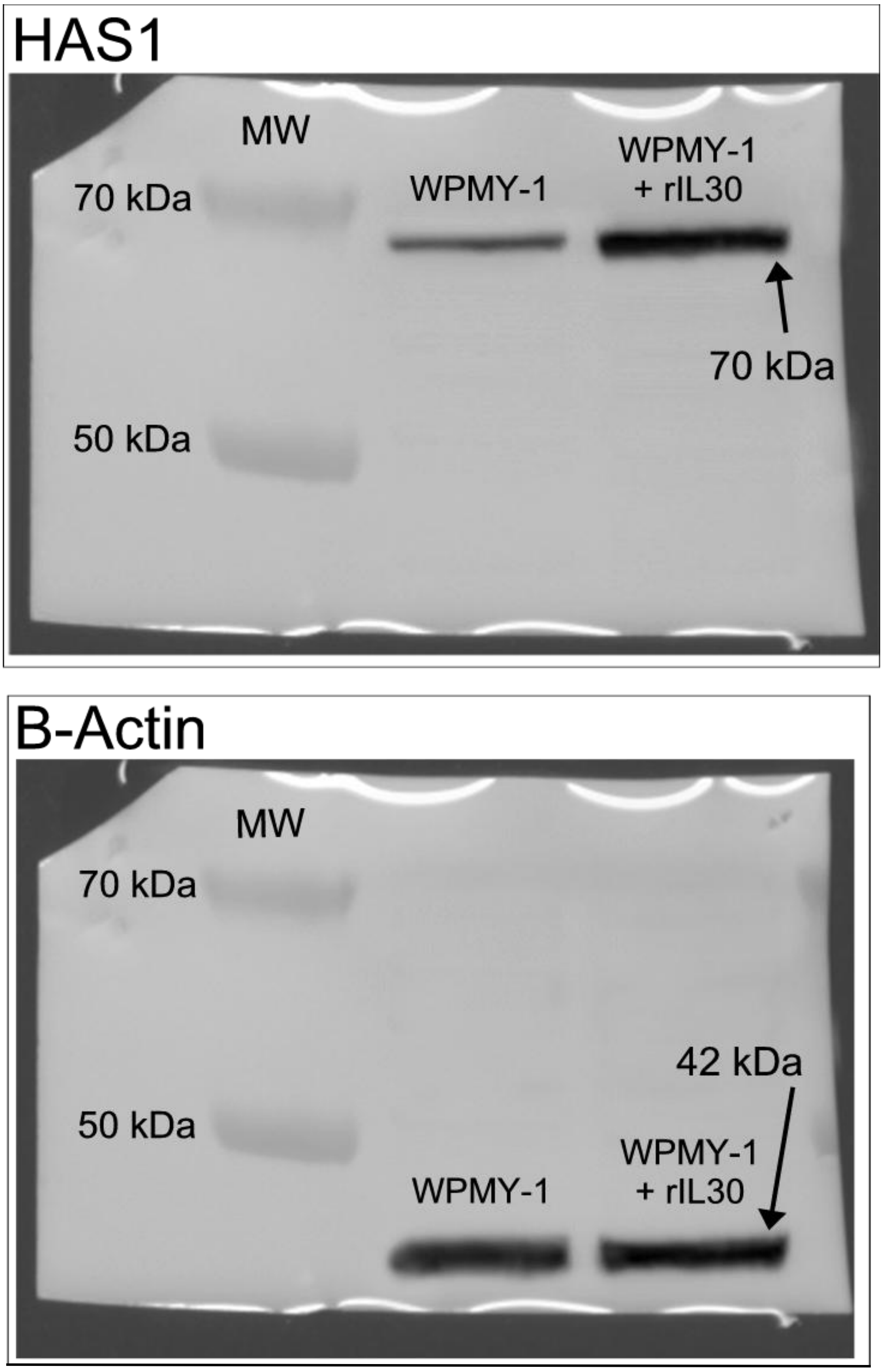

MW: molecular weight marker.

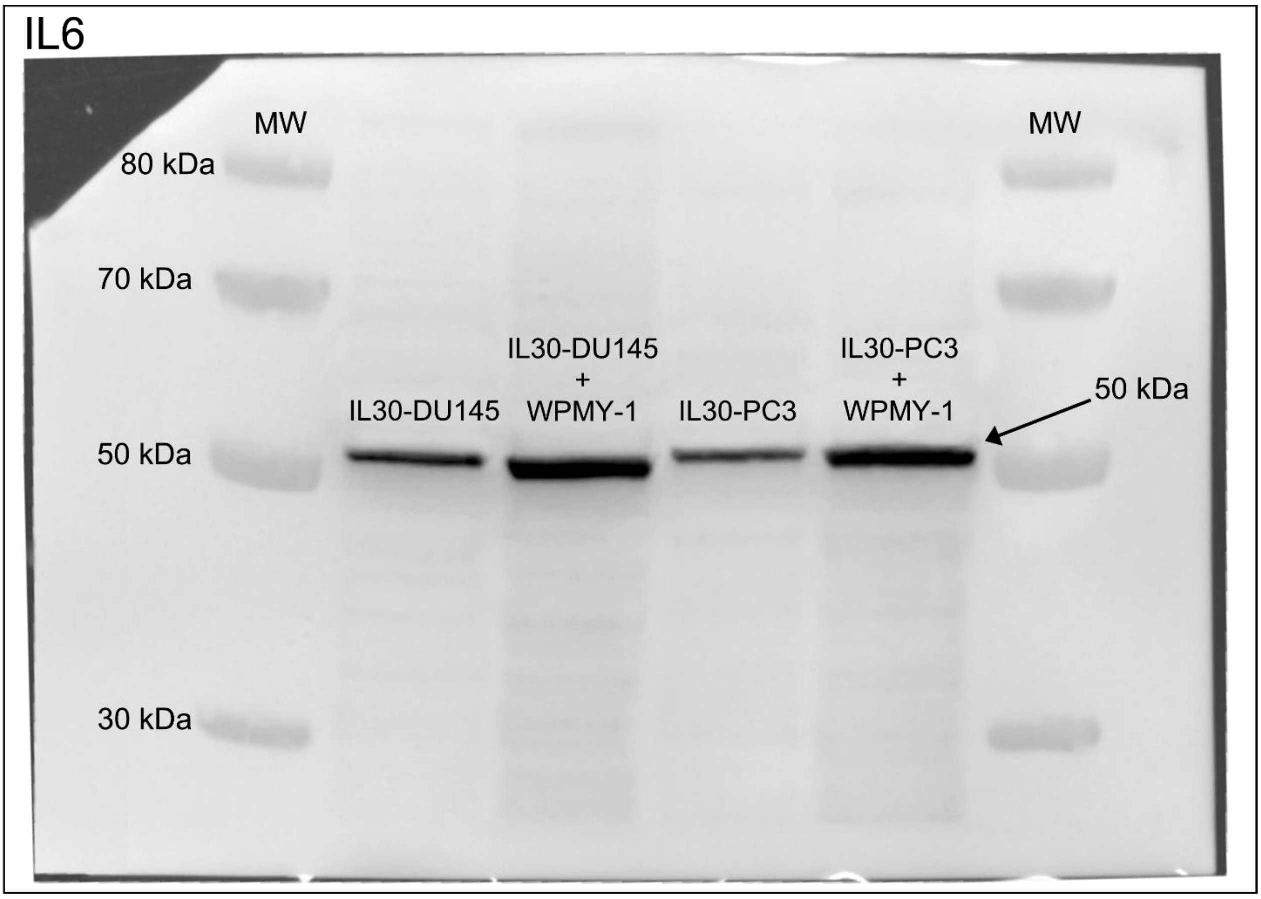

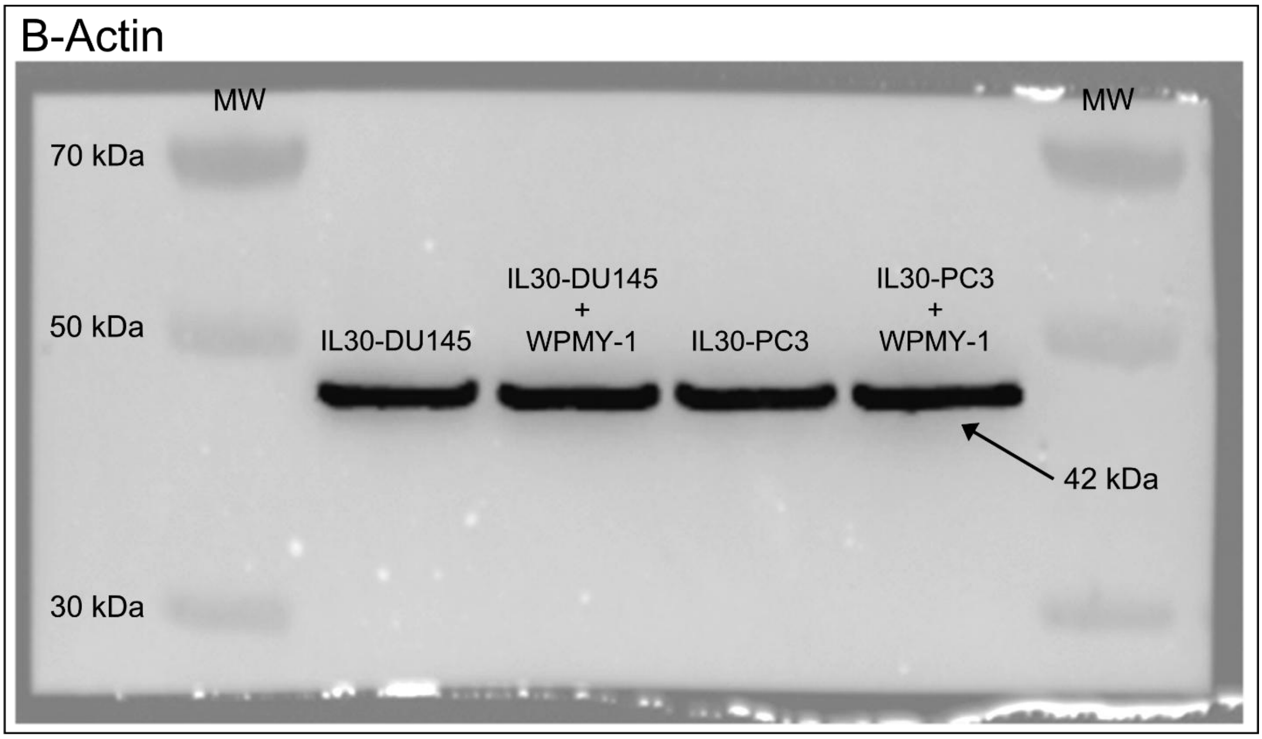

MW: molecular weight marker.

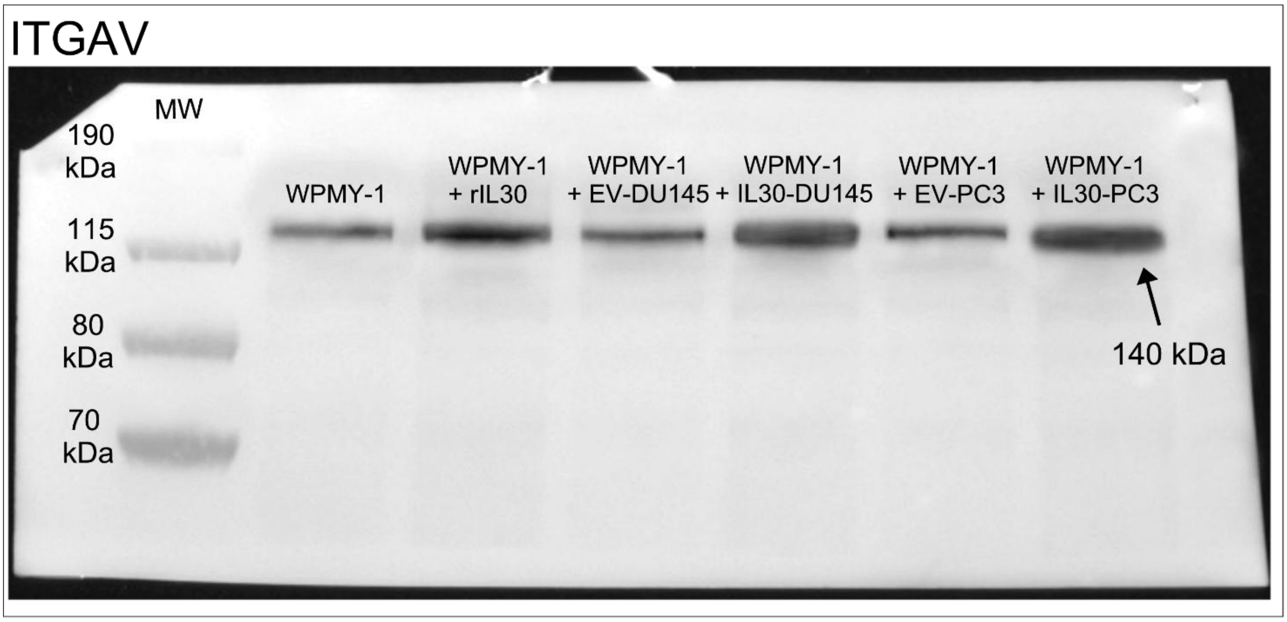

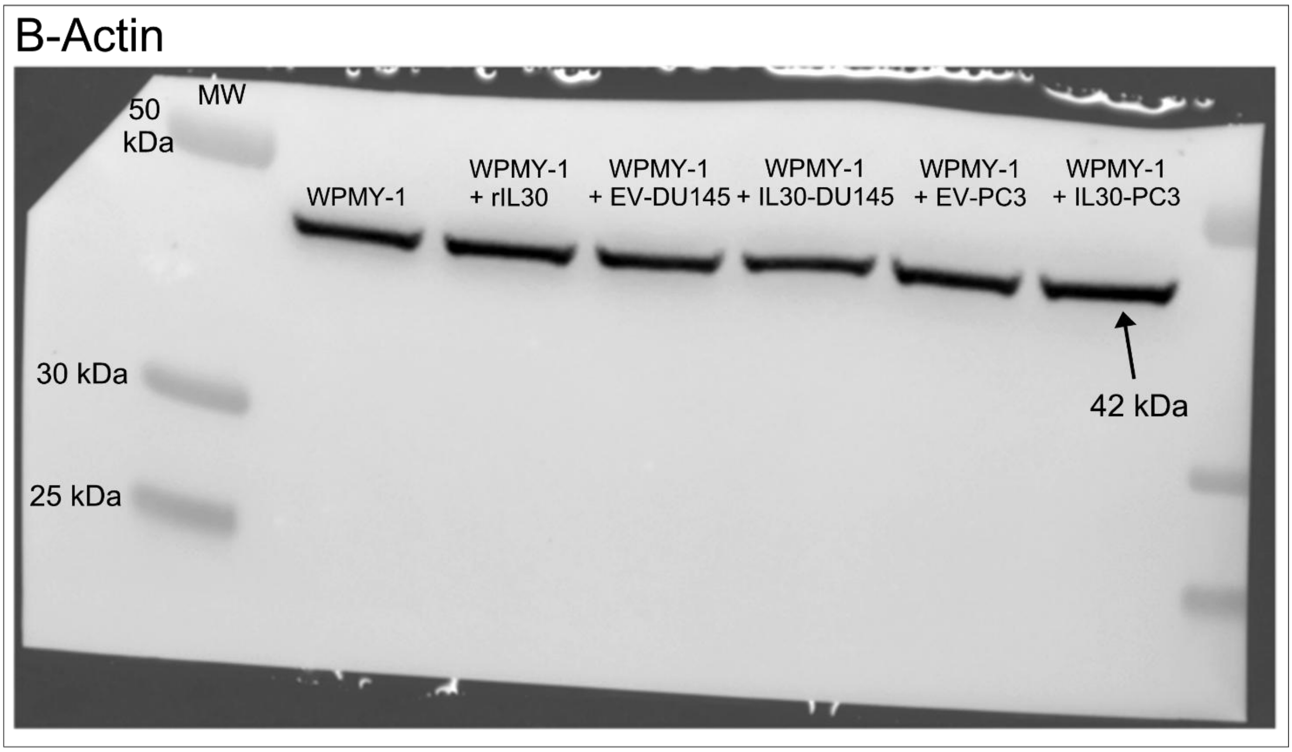

MW: molecular weight marker.

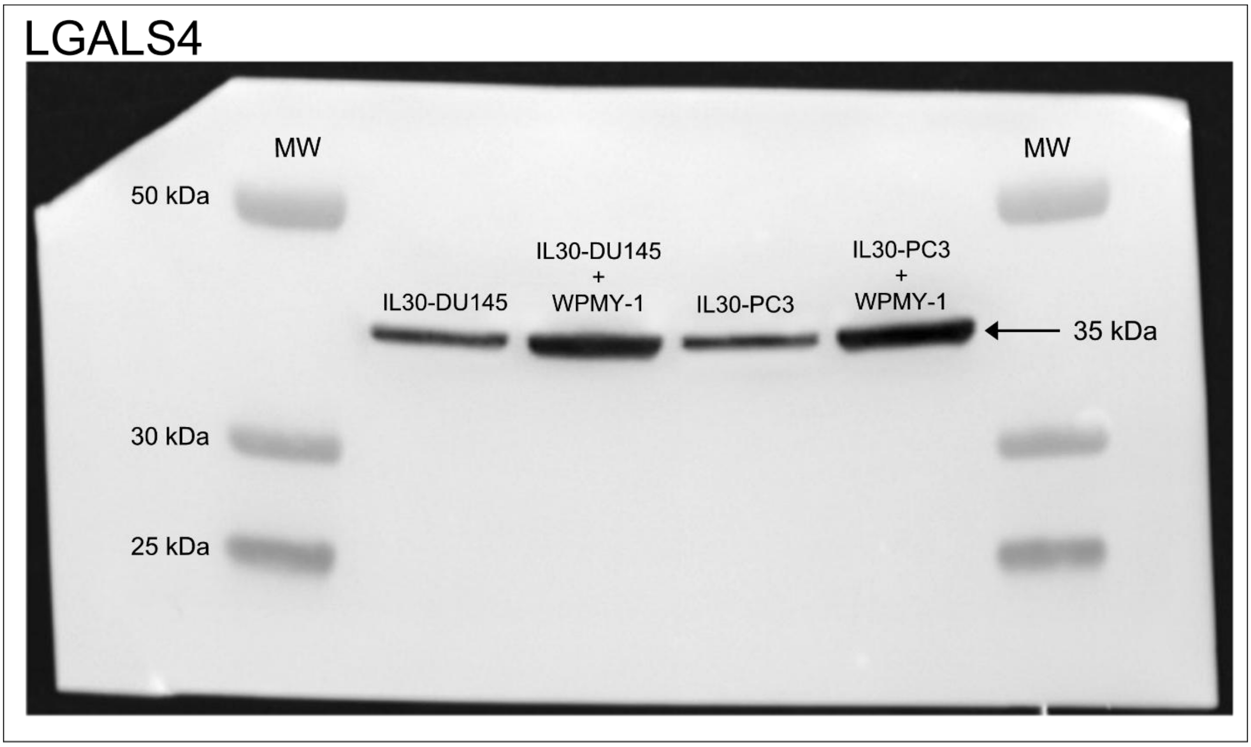

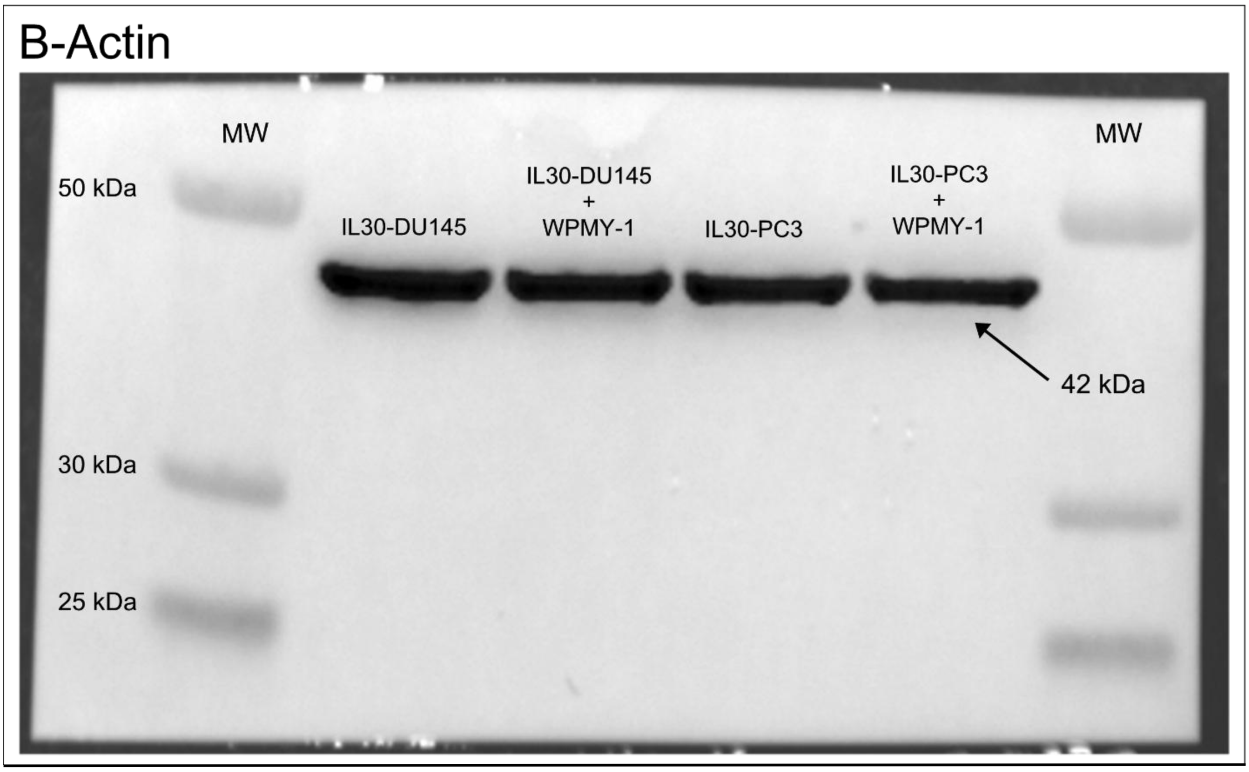

MW: molecular weight marker.

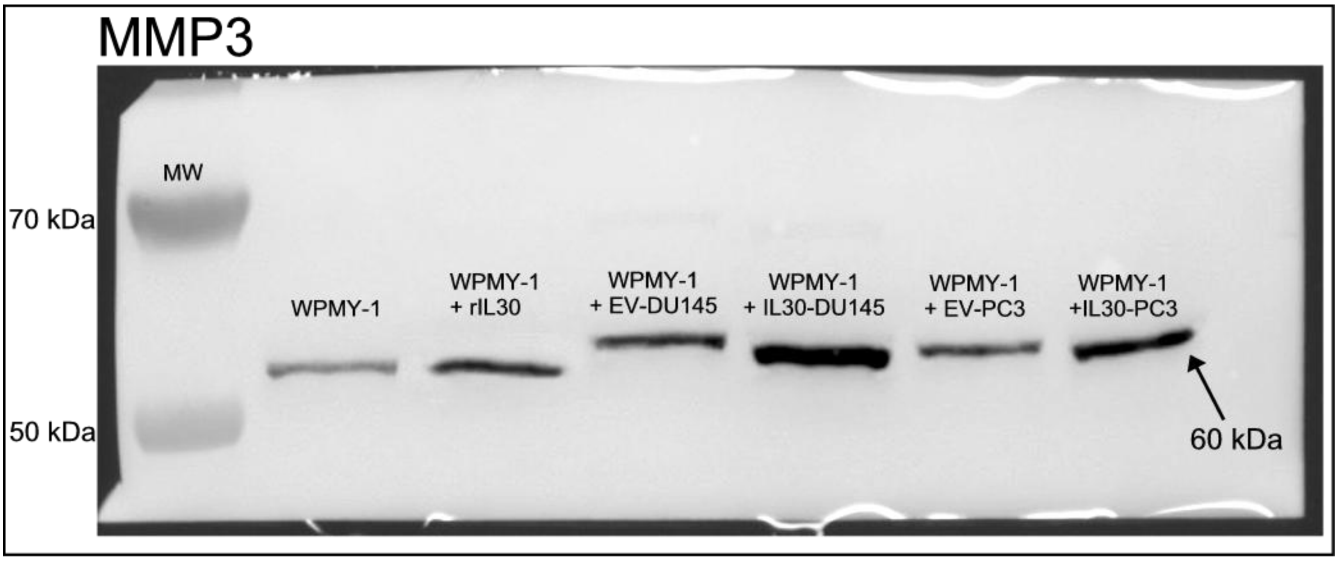

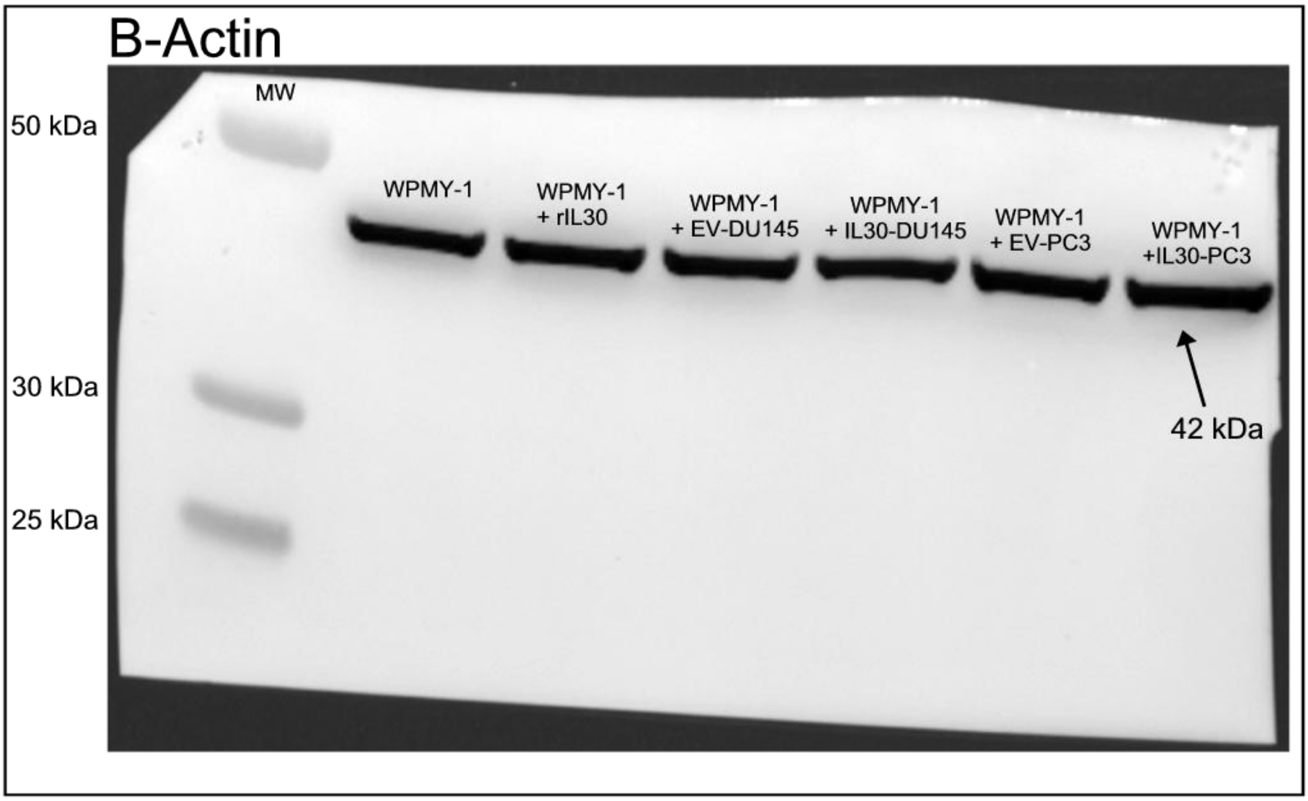

MW: molecular weight marker.

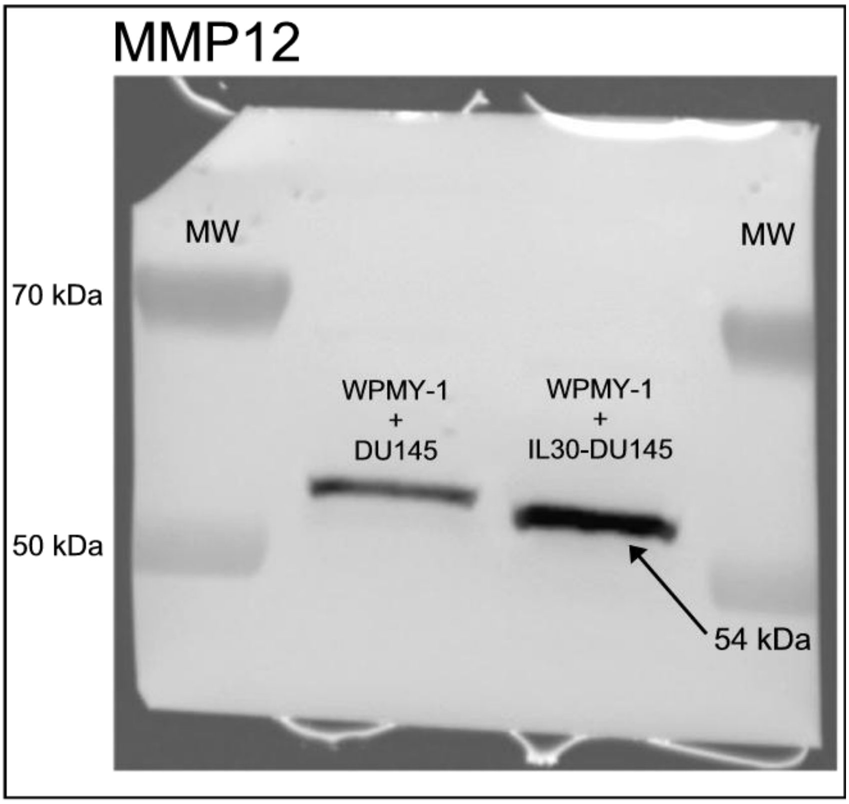

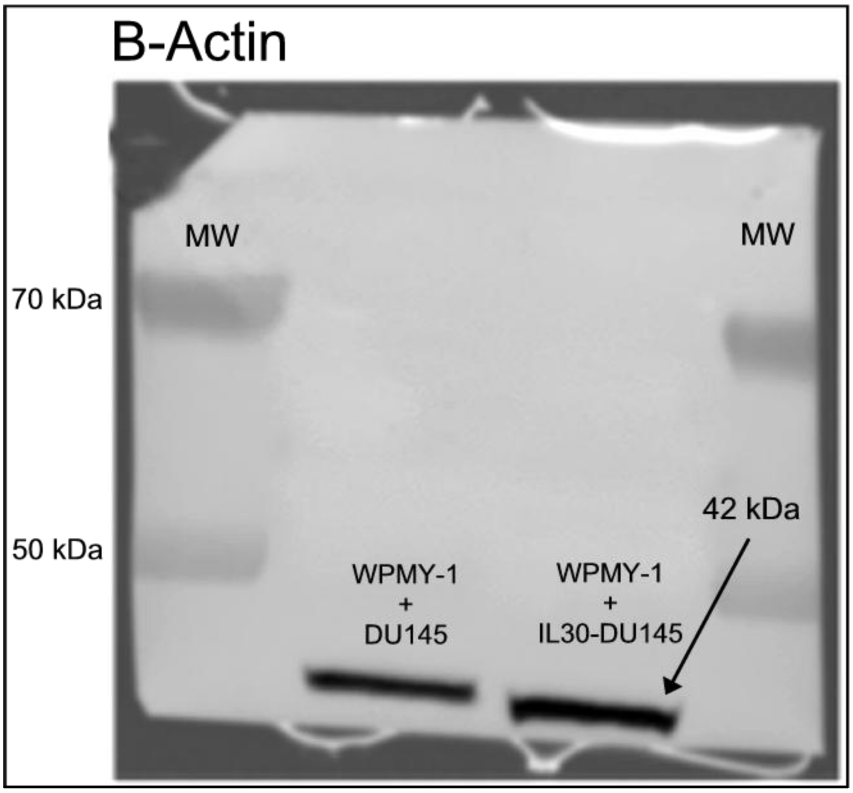

MW: molecular weight marker.

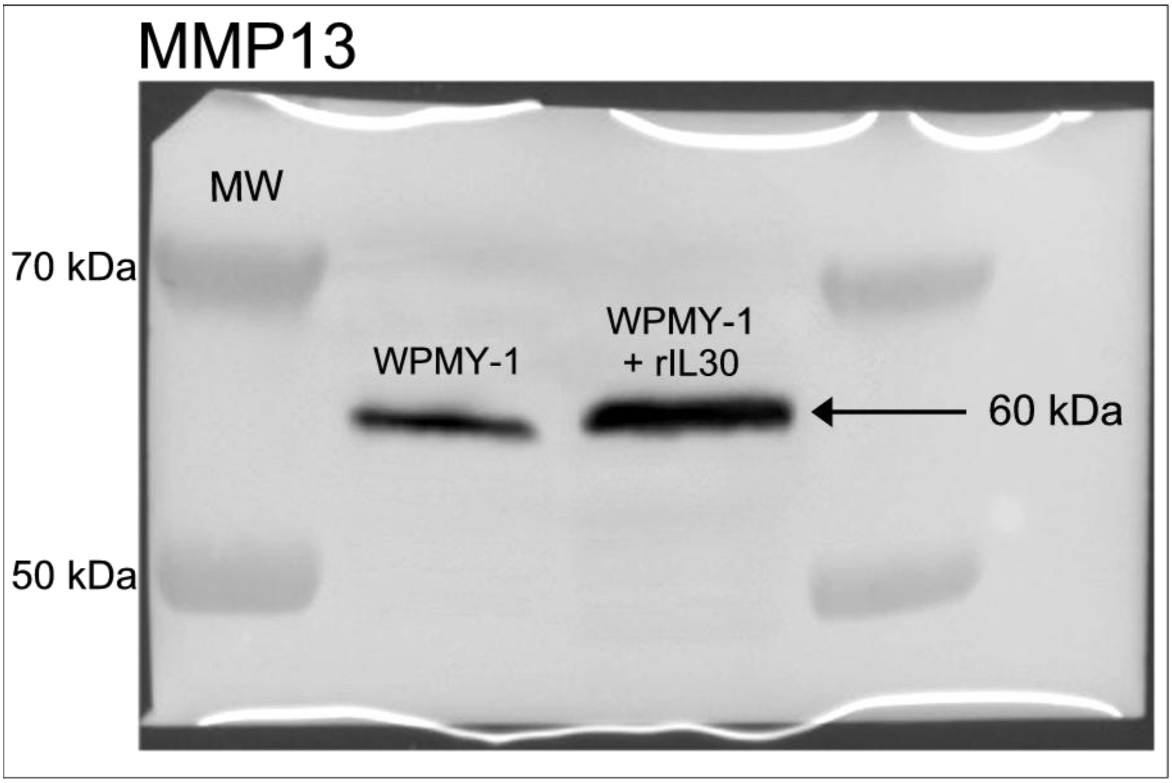

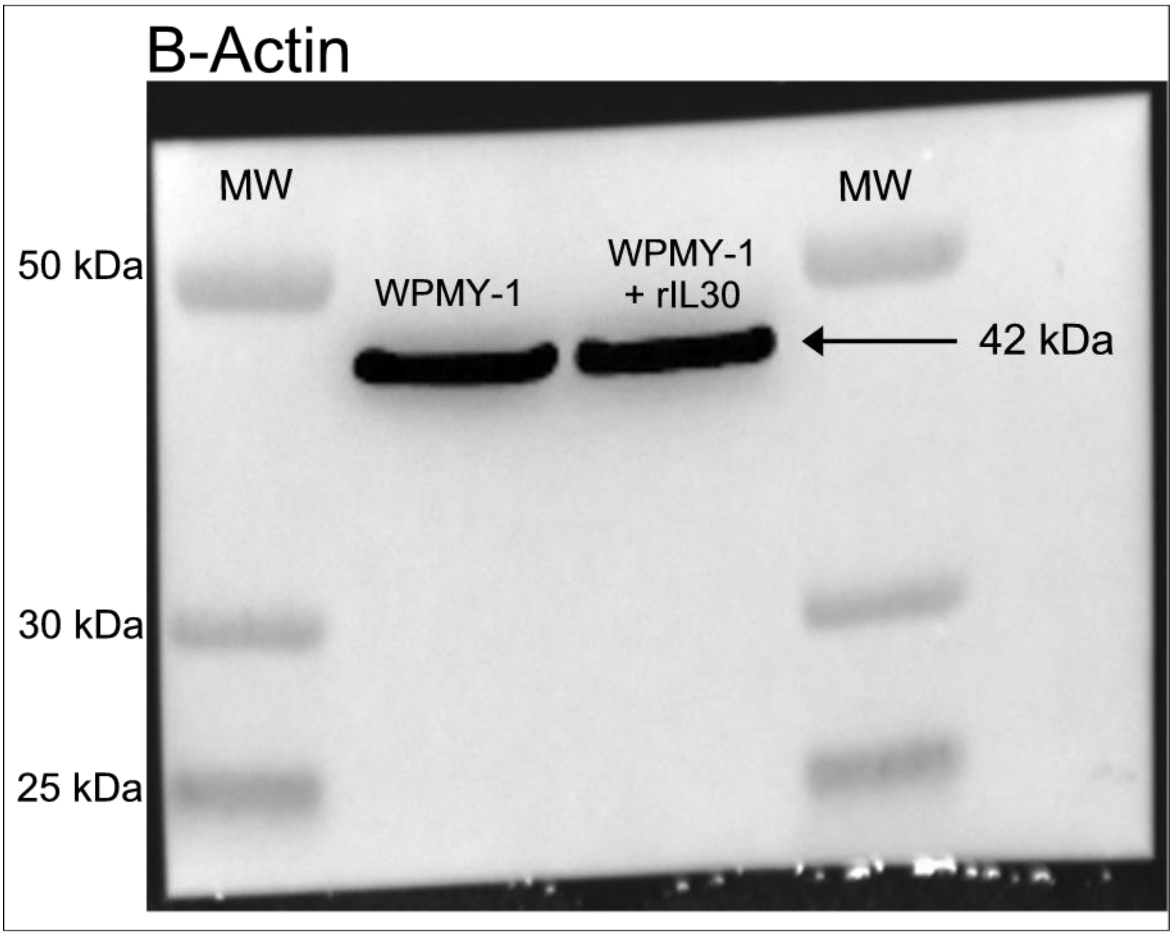

MW: molecular weight marker.

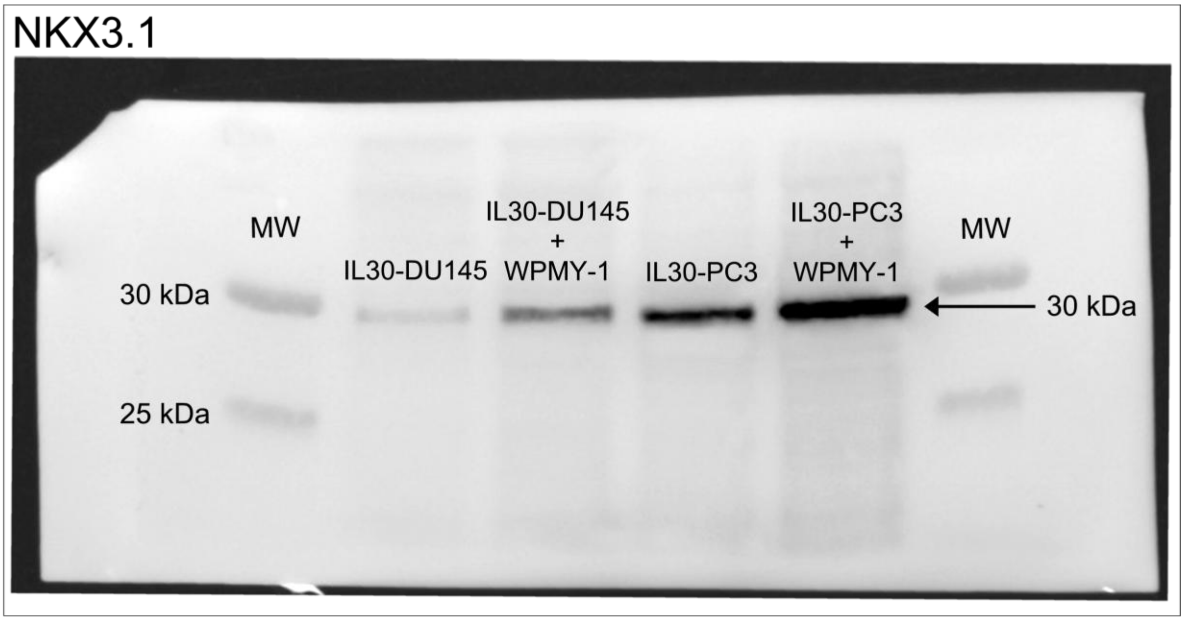

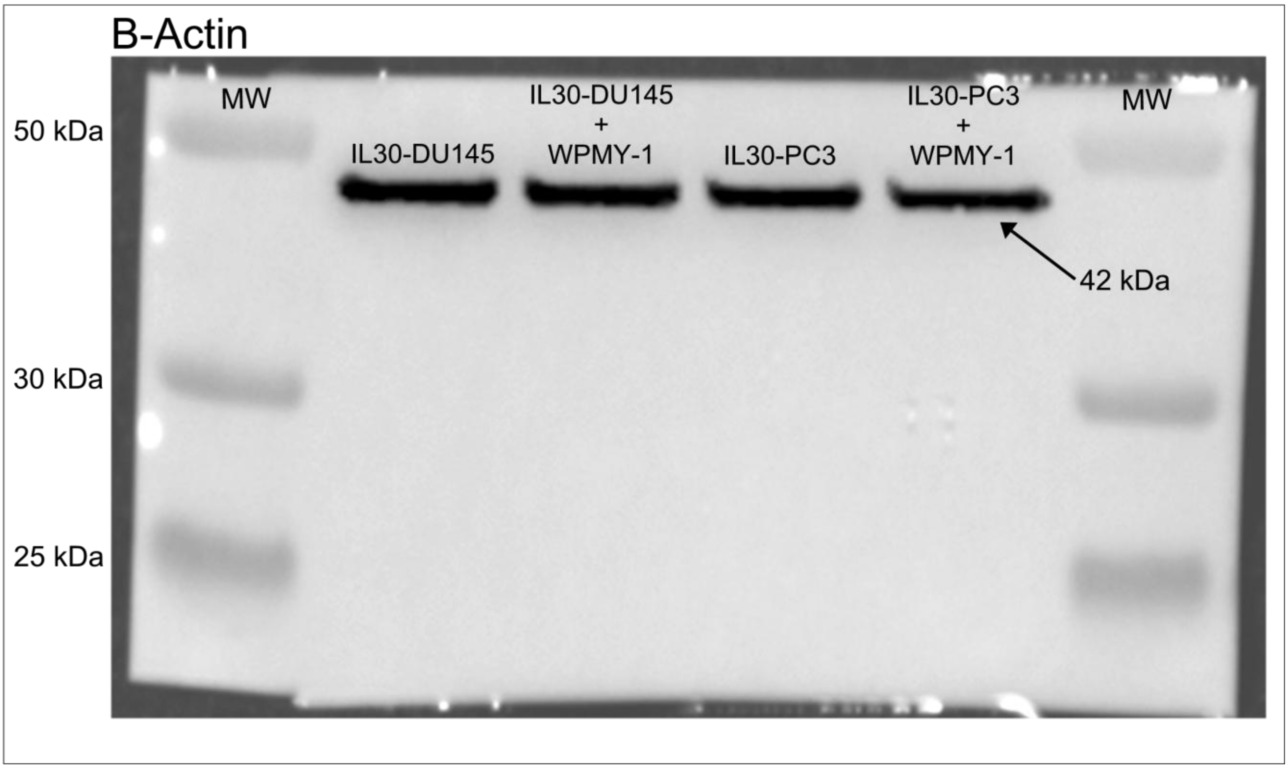

MW: molecular weight marker.

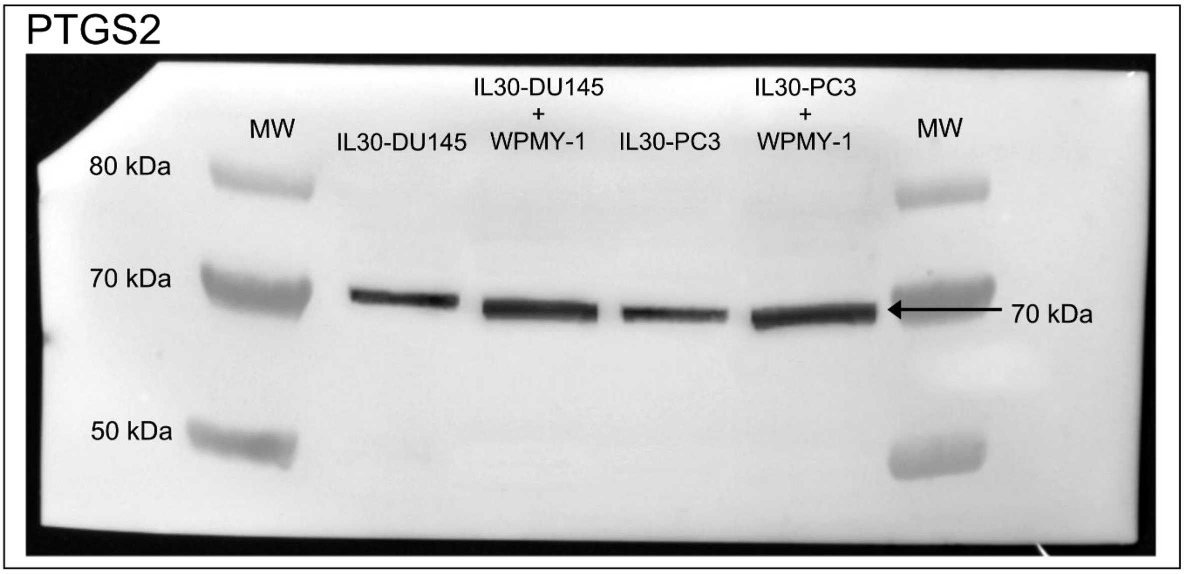

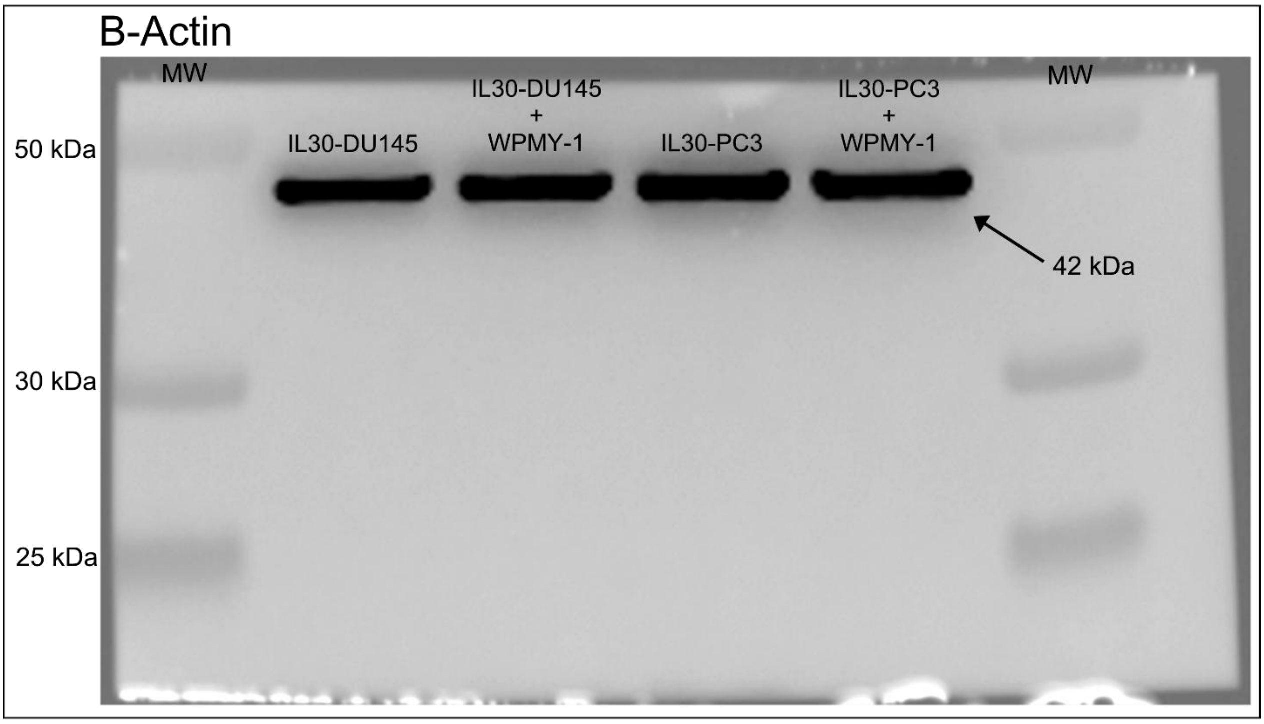

MW: molecular weight marker.

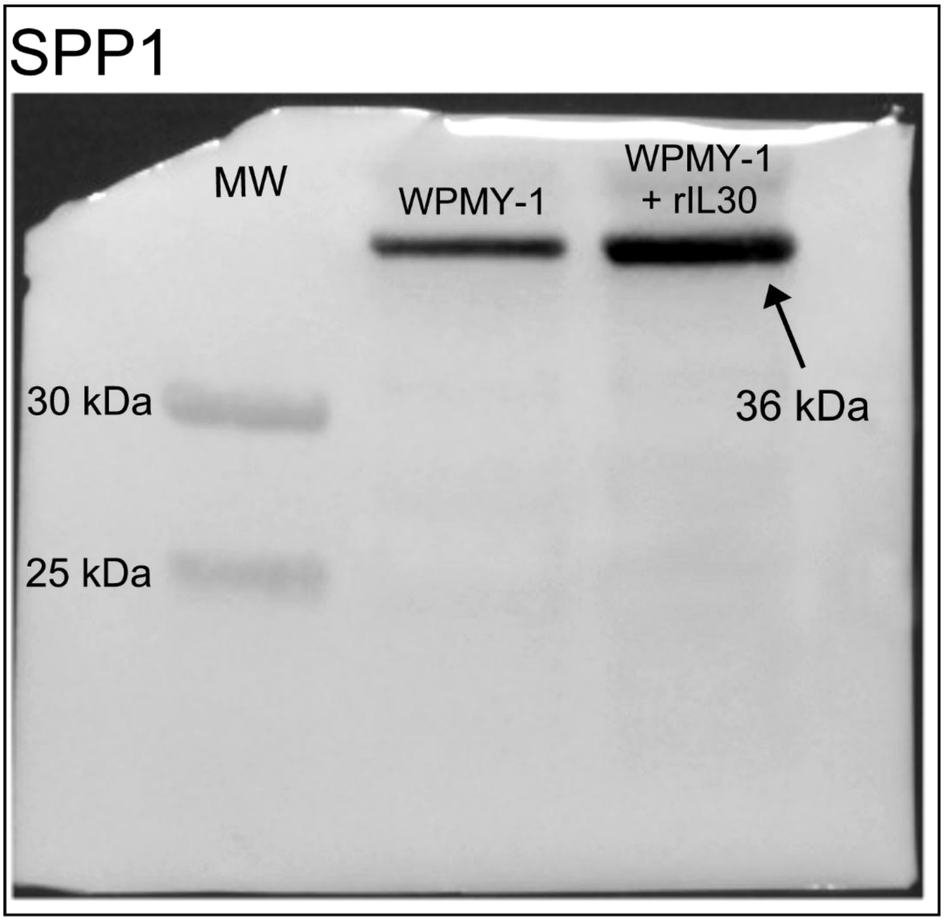

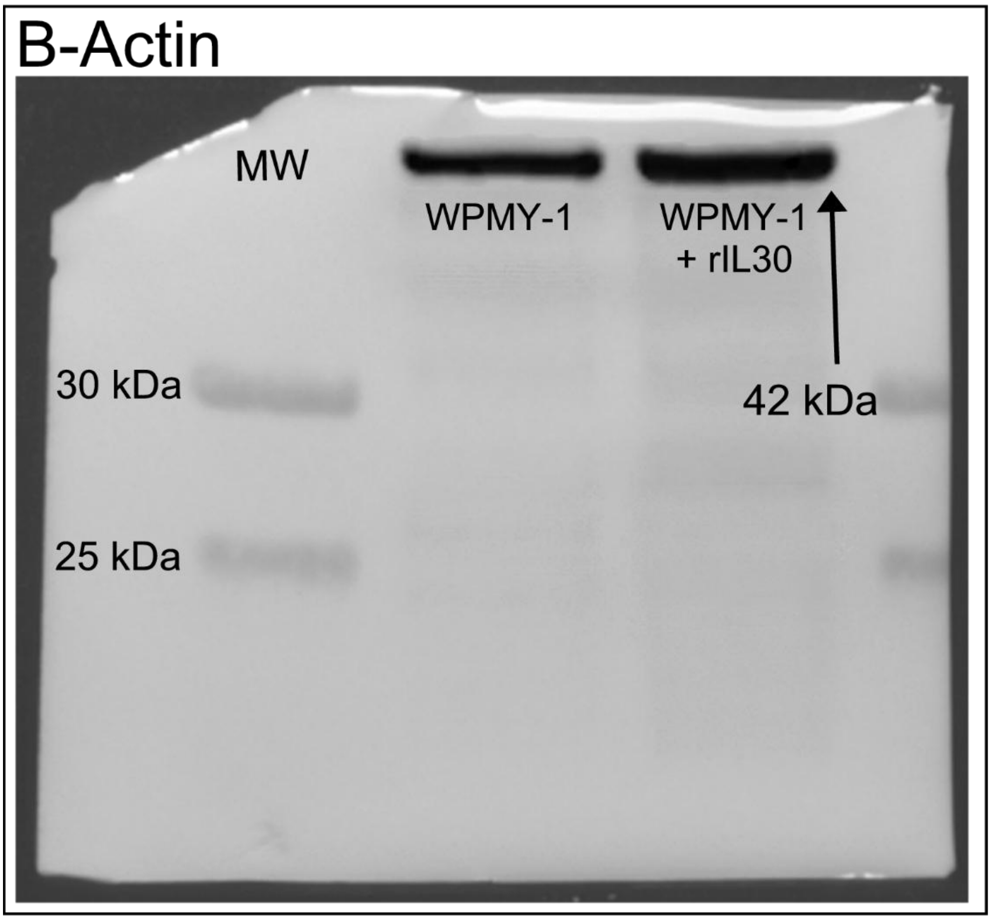

MW: molecular weight marker.

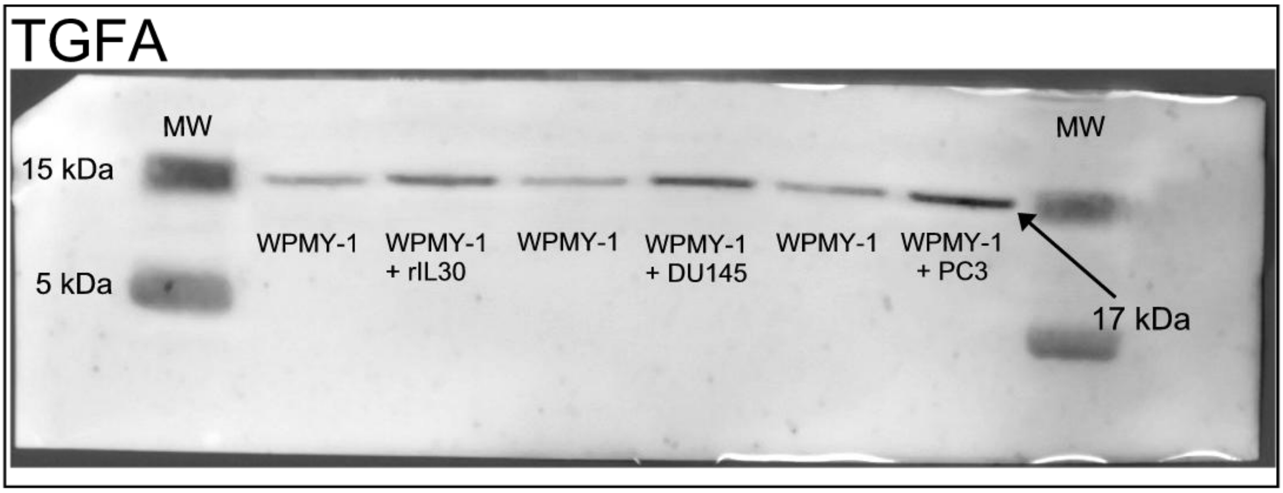

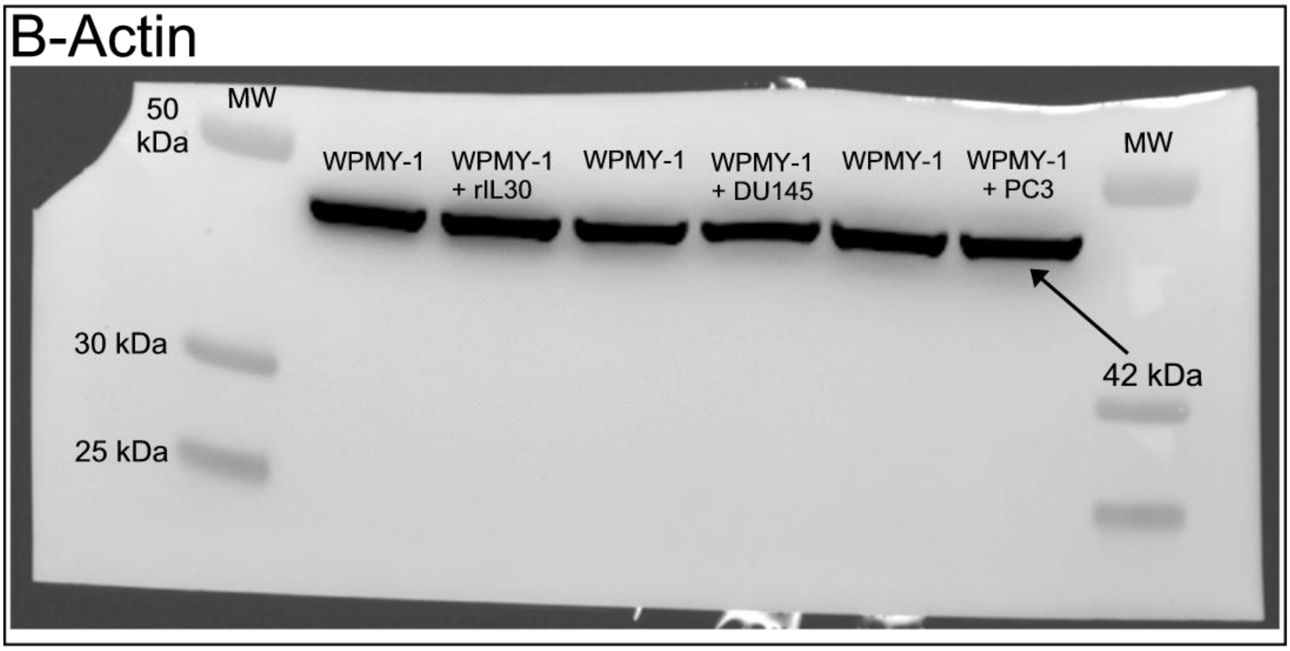

MW: molecular weight marker.

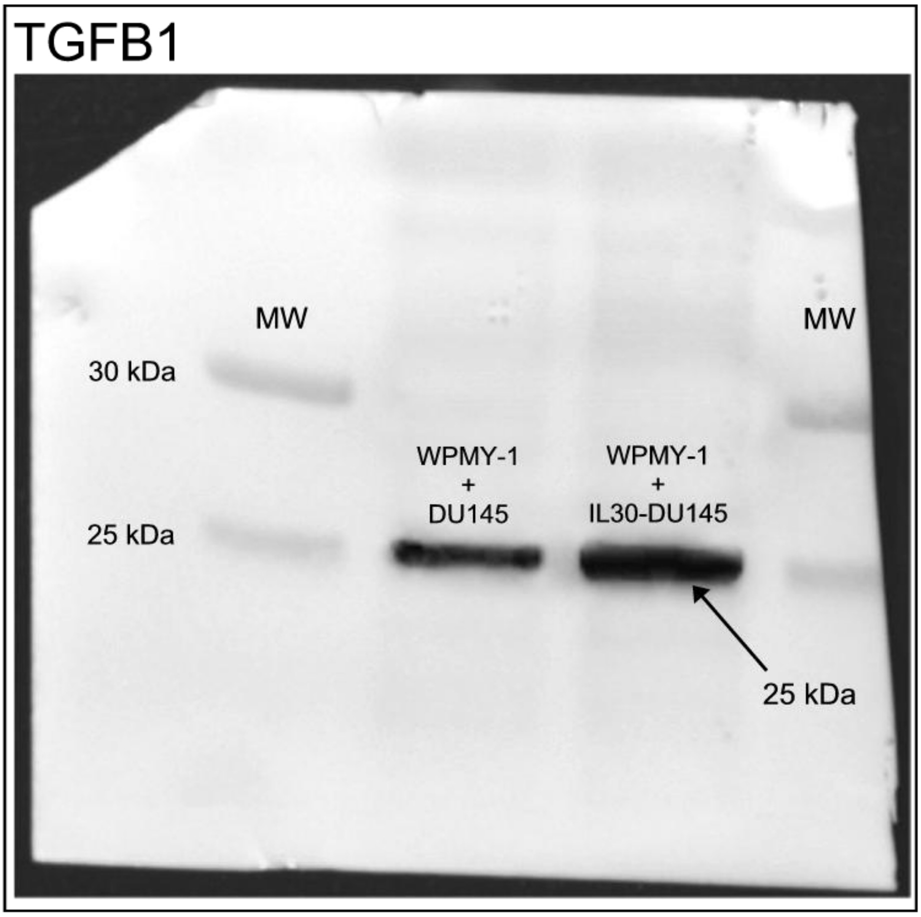

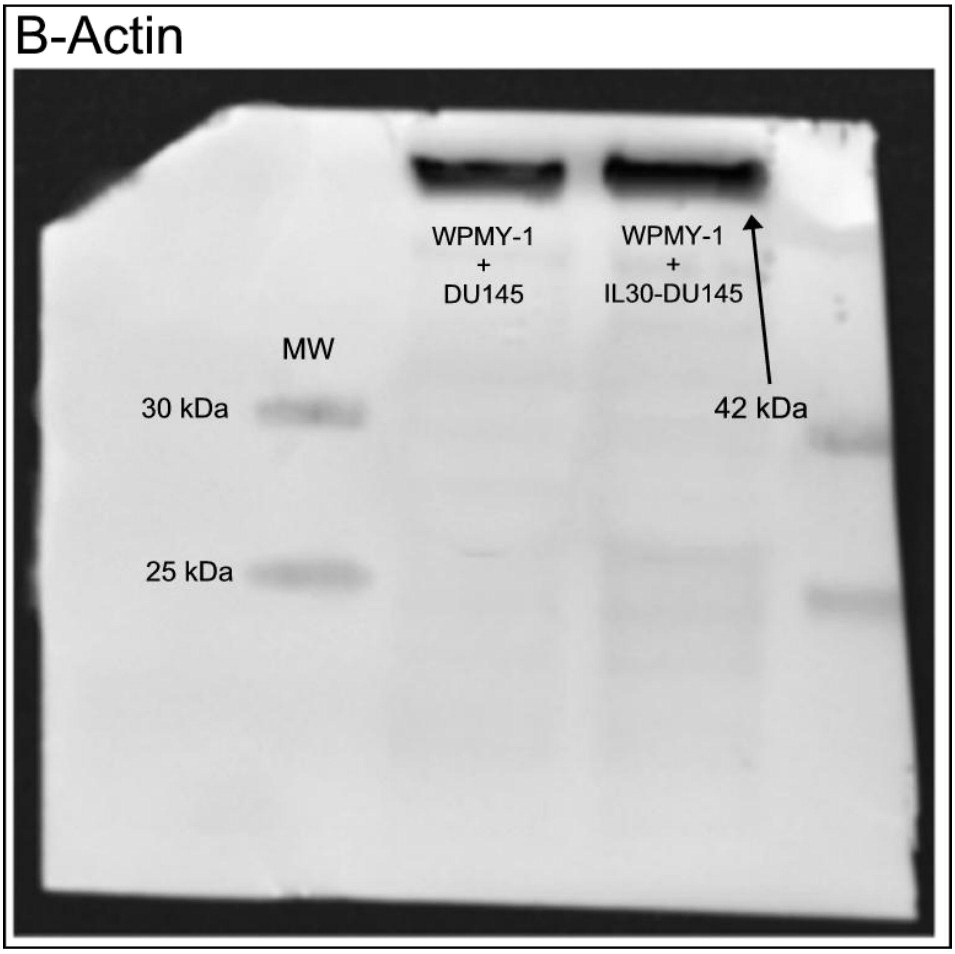

MW: molecular weight marker.

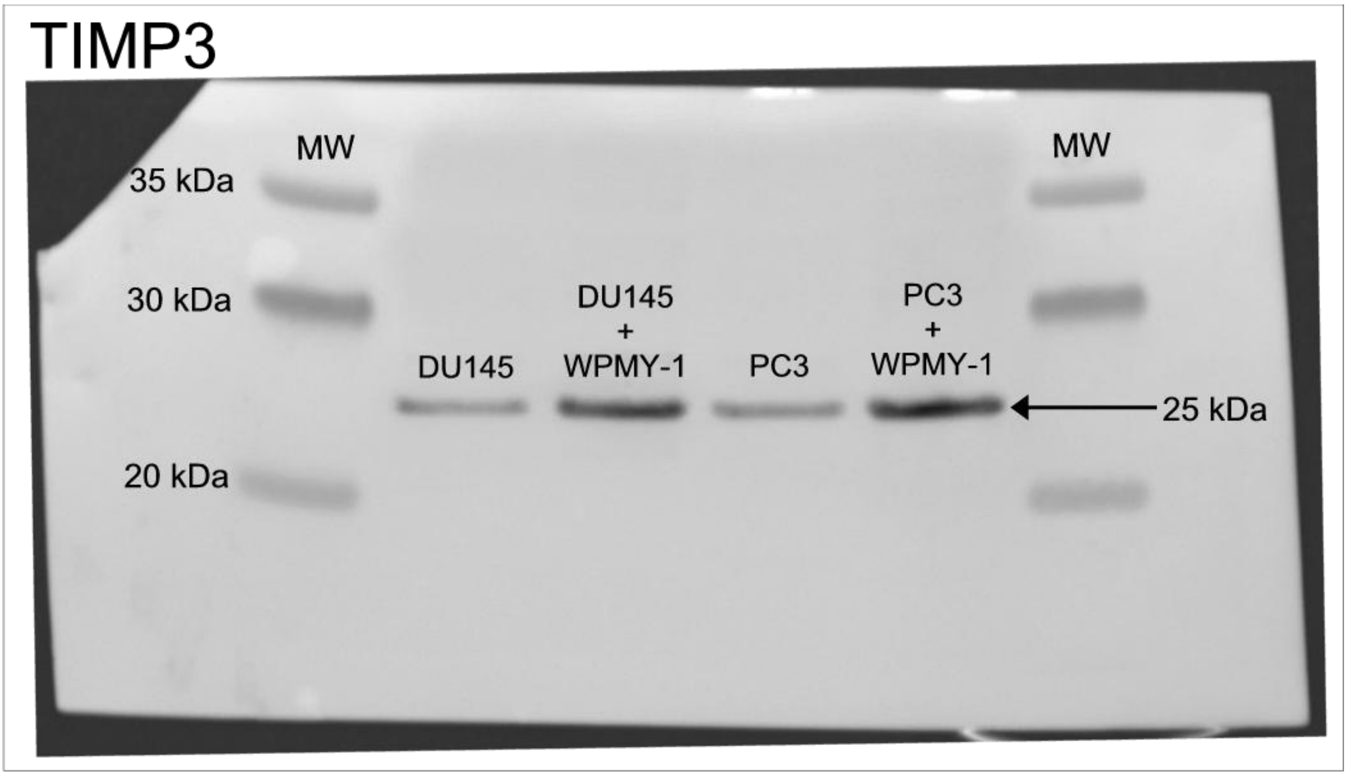

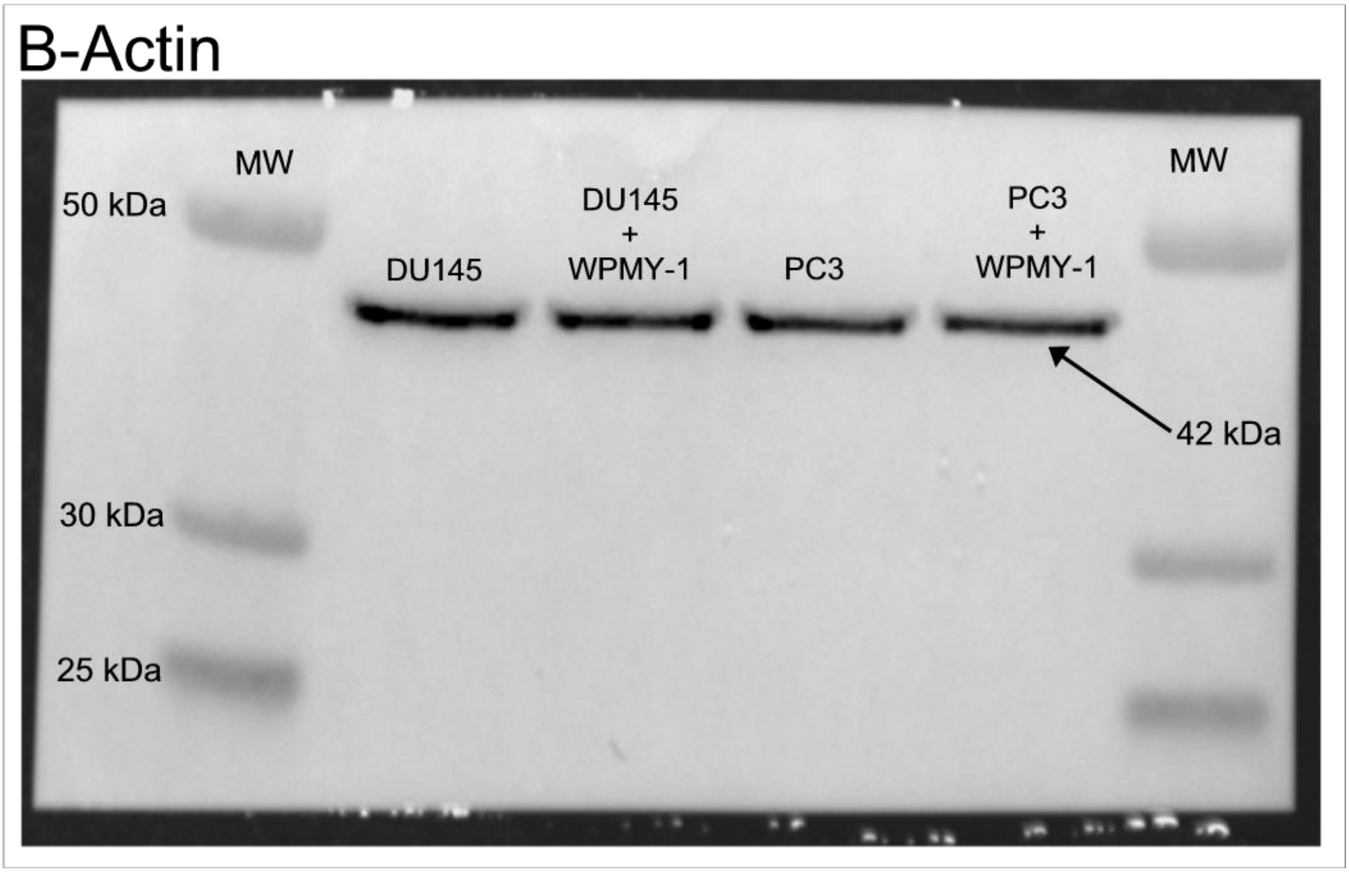

MW: molecular weight marker.

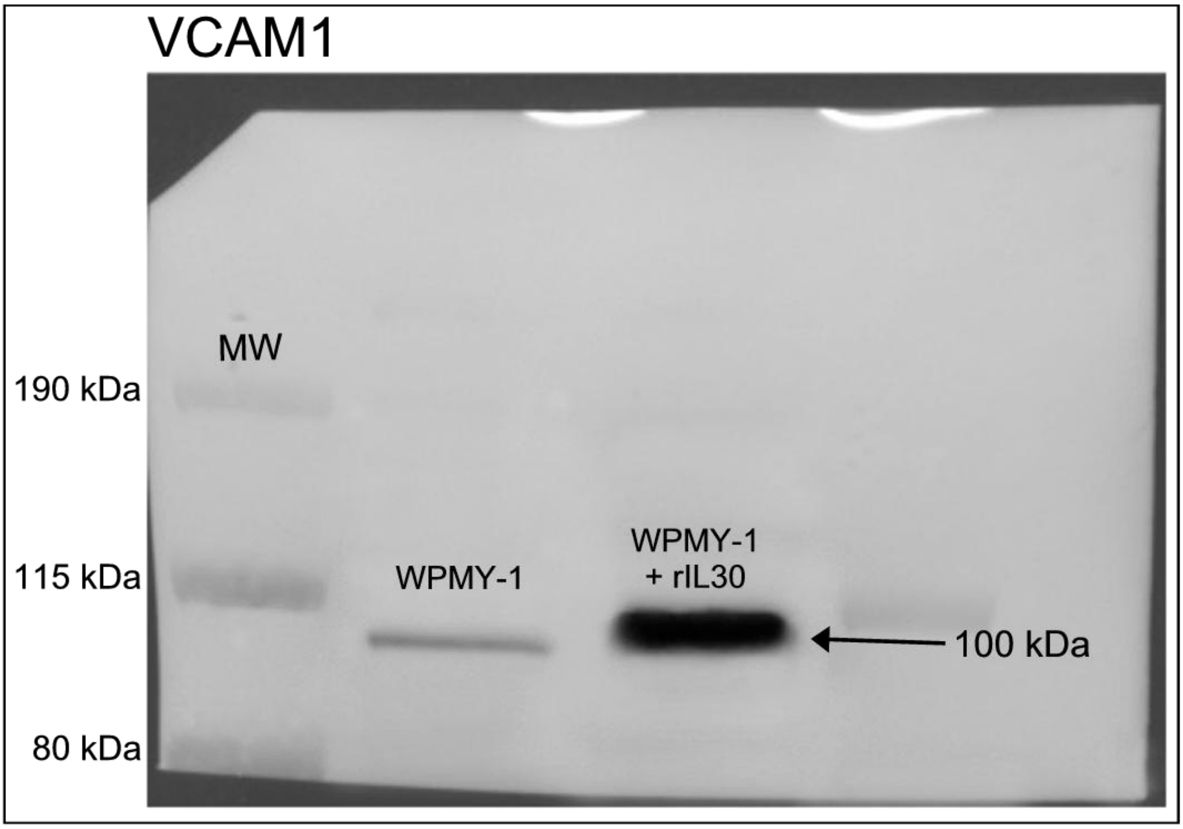

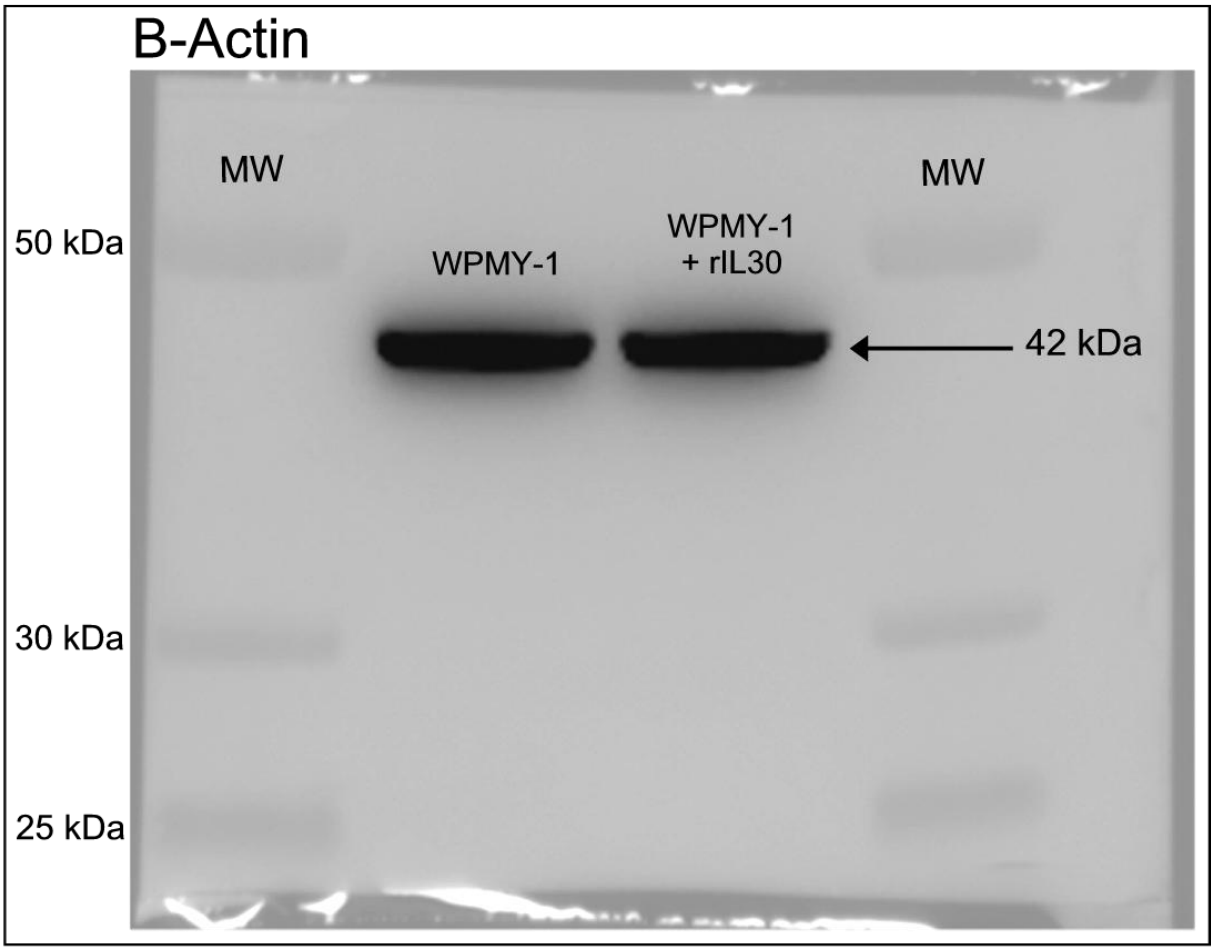

MW: molecular weight marker.

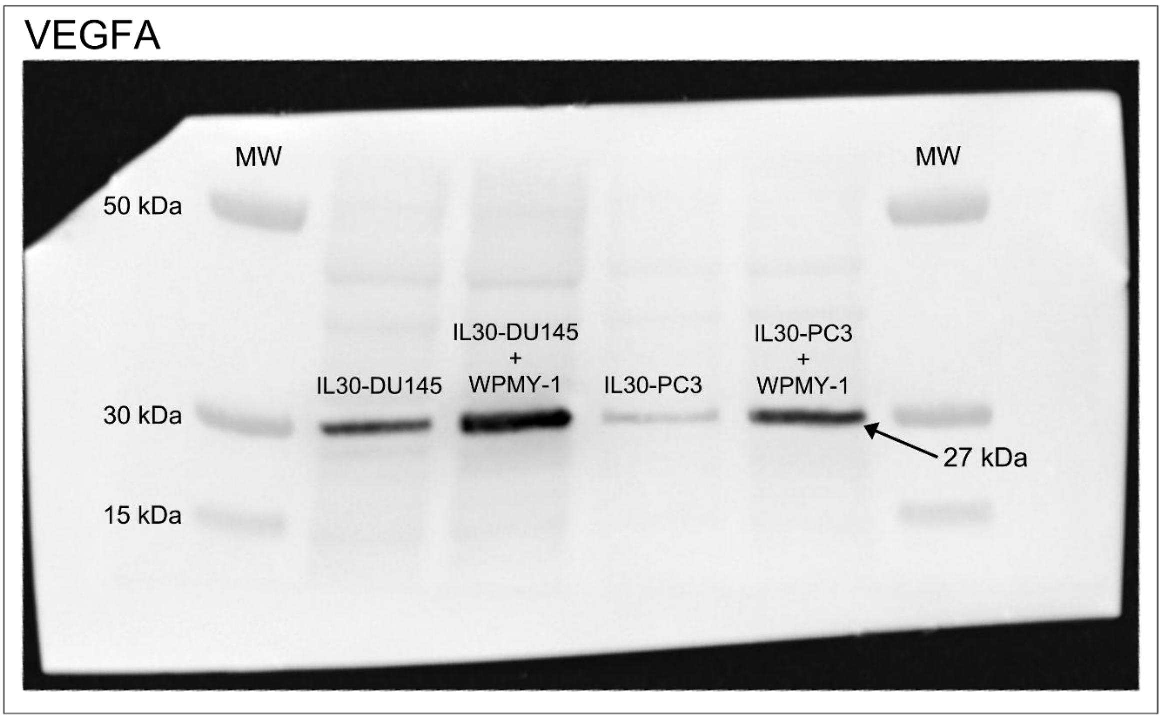

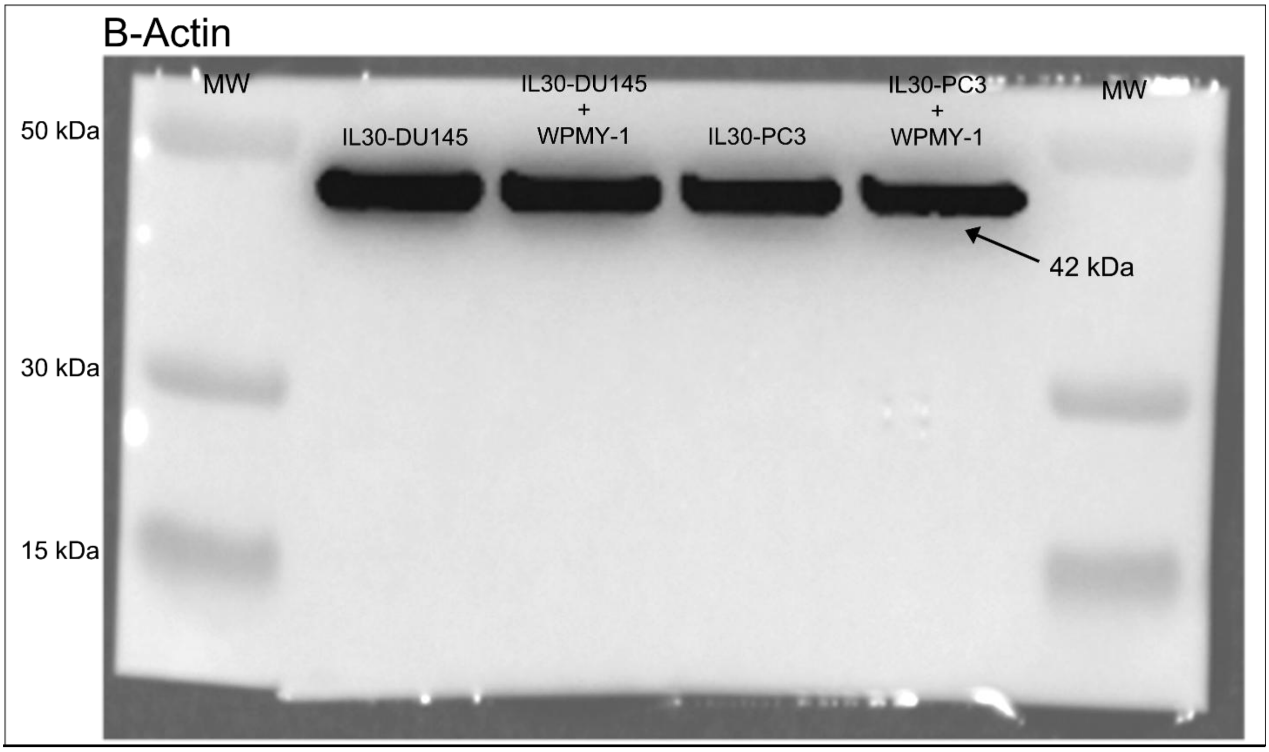

MW: molecular weight marker.
